# Supplementary material for: Pan-Cancer Analysis Reveals the Prognostic Potential of the THAP9/THAP9-AS1 Sense–Antisense Gene Pair in Human Cancers
Source: Noncoding RNA. 2022 Jul 8;8(4):51. doi: 10.3390/ncrna8040051 (PMC9326536; doi:10.3390/ncrna8040051)

(a) Predicted Transcription Start site for THAP9

General Information

Promoter ID:	THAP9_1
Promoter type:	region
Organism:	Homo sapiens (Human)
Gene Symbol:	THAP9
Description of the gene:	THAP domain containing 9
Sequence:	agcccgcctttcgtgacaaataaaggtcgtagccgcagagtcaacgggcGGAGCTAAAGT
Position in the genome:	Chromosome [NC_000004.12]; Strand [+]; Position [82900735]
Ensembl:	ENSG00000168152
RefSeq:	NM_001317776
NCBI Gene:	THAP9
GeneCards:	THAP9

(b) Predicted Transcription Start sites for THAP9-AS1

General Information

Promoter ID:	THAP9-AS1_1
Promoter type:	region
Organism:	Homo sapiens (Human)
Gene Symbol:	THAP9-AS1
Description of the gene:	THAP9 antisense RNA 1 [Source:HGNC Symbol;Acc:HGNC:44172]
Sequence:	ccacgttgtgtcccagcagccaatcggaagggcctgagcctgaagacgtGCATTTGACCG
Position in the genome:	Chromosome [NC_000004.12]; Strand [-]; Position [82900569]
Ensembl:	ENSG00000251022
RefSeq:	NR_034075
NCBI Gene:	THAP9-AS1

General Information

Promoter ID:	THAP9-AS1_2
Promoter type:	region
Organism:	Homo sapiens (Human)
Gene Symbol:	THAP9-AS1
Description of the gene:	THAP9 antisense RNA 1 [Source:HGNC Symbol;Acc:HGNC:44172]
Sequence:	gtcacacacgcggcgcgacccacagtgcggcccggccccgccccacgccACGCCACGGCC
Position in the genome:	Chromosome [NC_000004.12]; Strand [-]; Position [82900944]
Ensembl:	ENSG00000251022
RefSeq:	NR_034075
NCBI Gene:	THAP9-AS1

(c) Predicted CpG Islands for THAP9

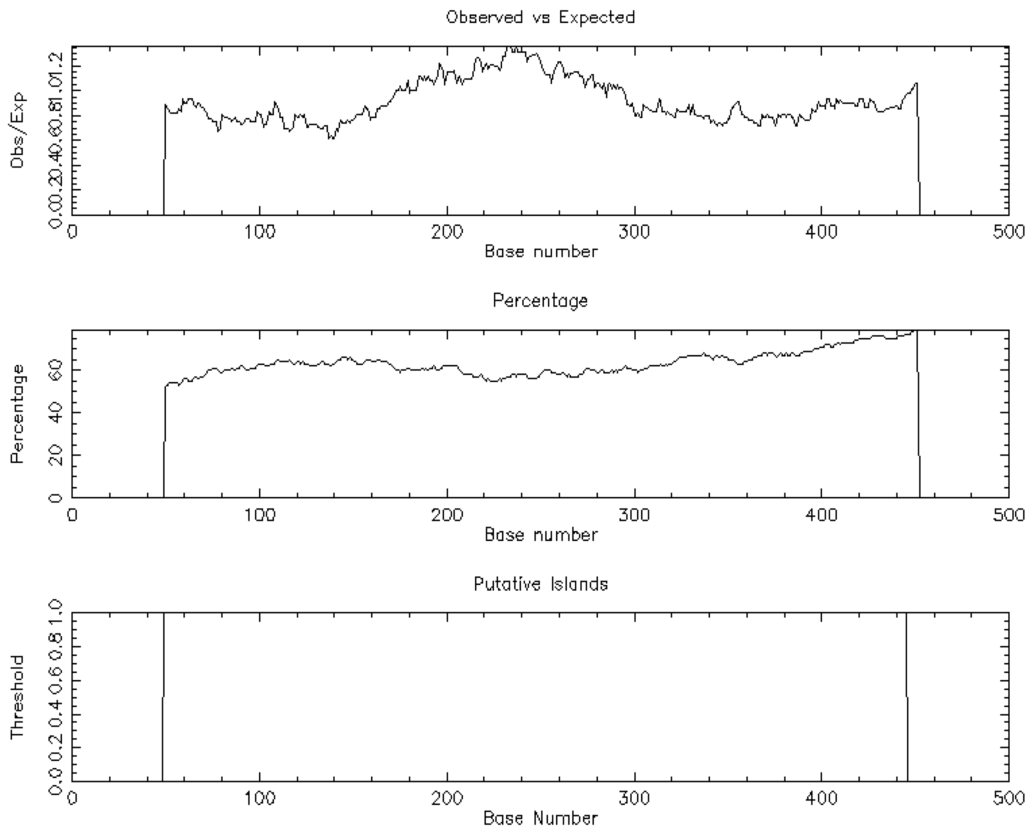

(d) Predicted CpG Islands for THAP9-AS1

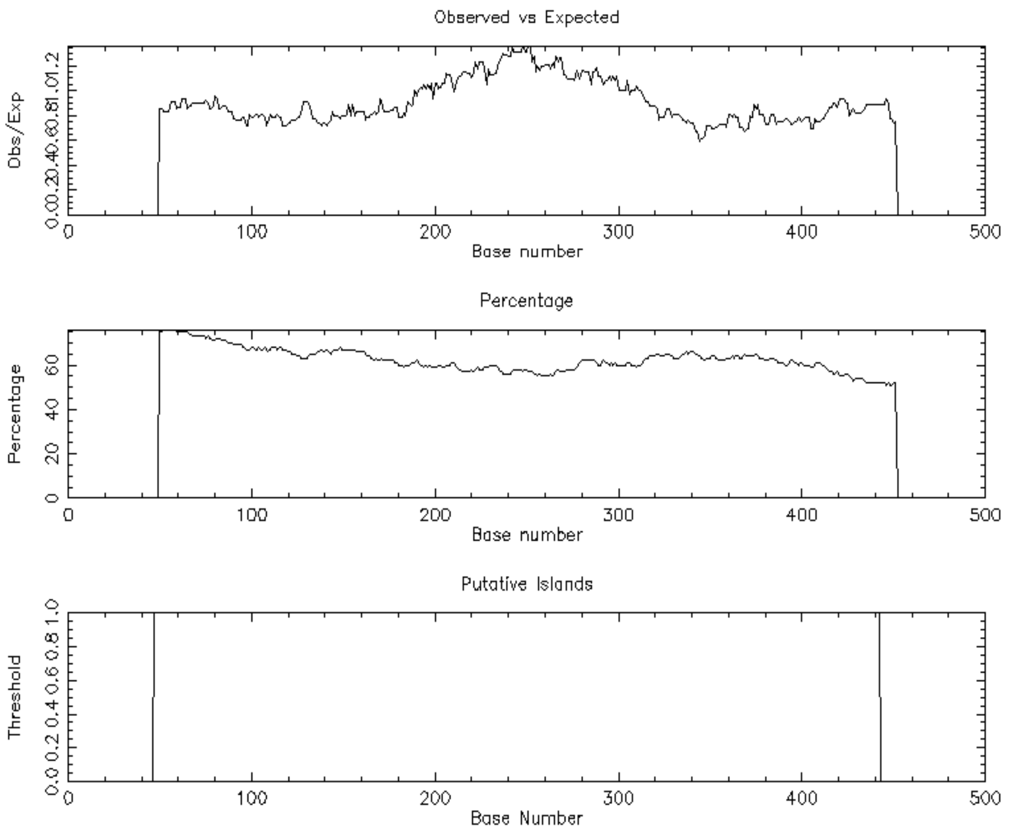

Supplement: Supplementary file 1 [file ncrna-08-00051-s001.zip › Supplementary Figure S1.pdf]
