# Supplementary material for: Pan-Cancer Analysis Reveals the Prognostic Potential of the THAP9/THAP9-AS1 Sense–Antisense Gene Pair in Human Cancers
Source: Noncoding RNA. 2022 Jul 8;8(4):51. doi: 10.3390/ncrna8040051 (PMC9326536; doi:10.3390/ncrna8040051)
Supplement: Supplementary file 1 [file ncrna-08-00051-s001.zip › Supplementary Figure S5.pdf]

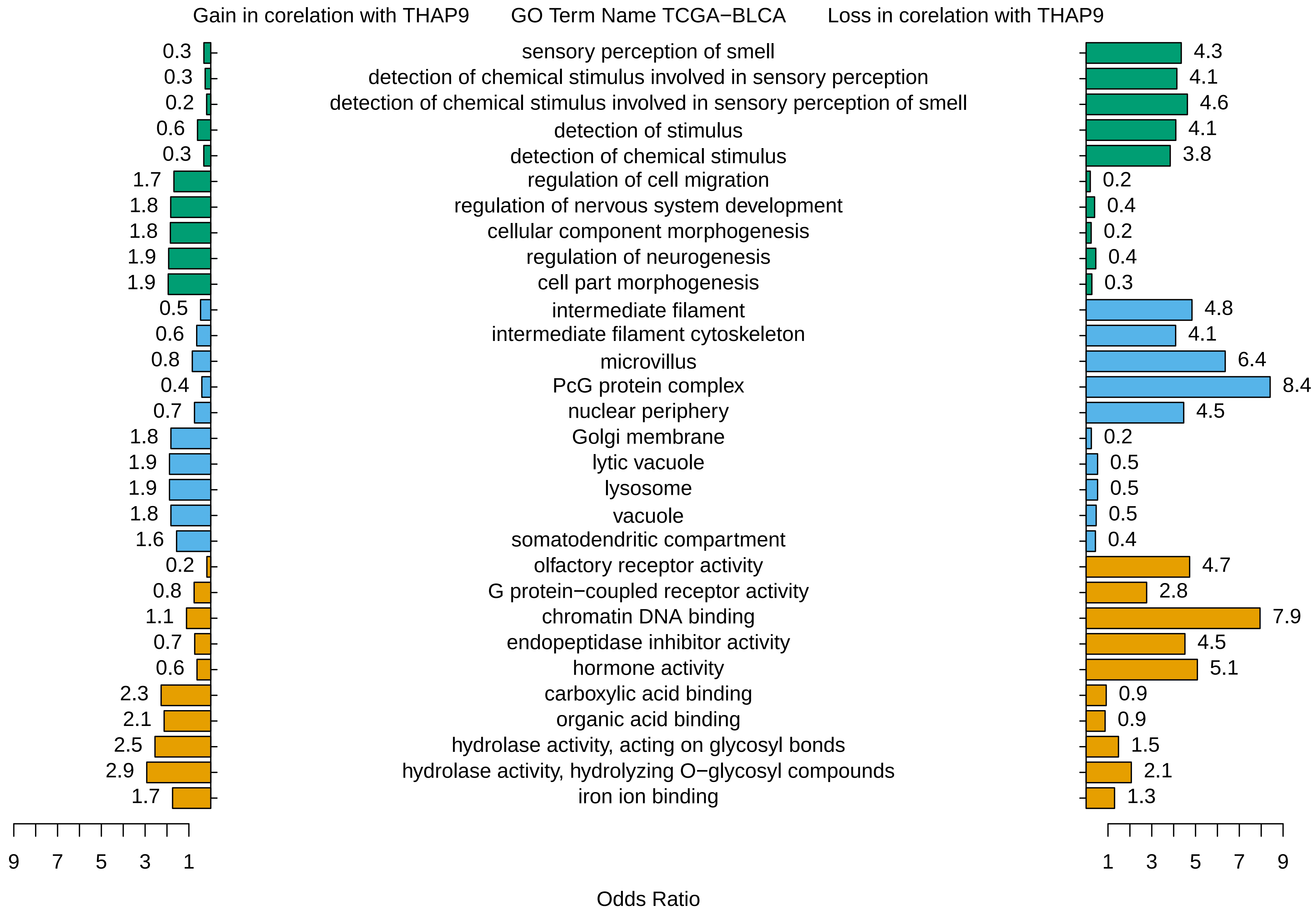

Gain in corelation with THAP9
GO Term Name TCGA−BLCA
Loss in corelation with THAP9
0.3 sensory perception of smell 4.3
0.3 detection of chemical stimulus involved in sensory perception 4.1
0.2 detection of chemical stimulus involved in sensory perception of smell 4.6
0.6 detection of stimulus 4.1
0.3 detection of chemical stimulus 3.8
1.7 regulation of cell migration 0.2
1.8 regulation of nervous system development 0.4
1.8 cellular component morphogenesis 0.2
1.9 regulation of neurogenesis 0.4
1.9 cell part morphogenesis 0.3
0.5 intermediate filament 4.8
0.6 intermediate filament cytoskeleton 4.1
0.8 microvillus 6.4
0.4 PcG protein complex 8.4
0.7 nuclear periphery 4.5
1.8 Golgi membrane 0.2
1.9 lytic vacuole 0.5
1.9 lysosome 0.5
1.8 vacuole 0.5
1.6 somatodendritic compartment 0.4
0.2 olfactory receptor activity 4.7
0.8 G protein−coupled receptor activity 2.8
1.1 chromatin DNA binding 7.9
0.7 endopeptidase inhibitor activity 4.5
0.6 hormone activity 5.1
2.3 carboxylic acid binding 0.9
2.1 organic acid binding 0.9
2.5 hydrolase activity, acting on glycosyl bonds 1.5
2.9 hydrolase activity, hydrolyzing O−glycosyl compounds 2.1
1.7 iron ion binding 1.3
9 7 5 3 1
1 3 5 7 9
Odds Ratio

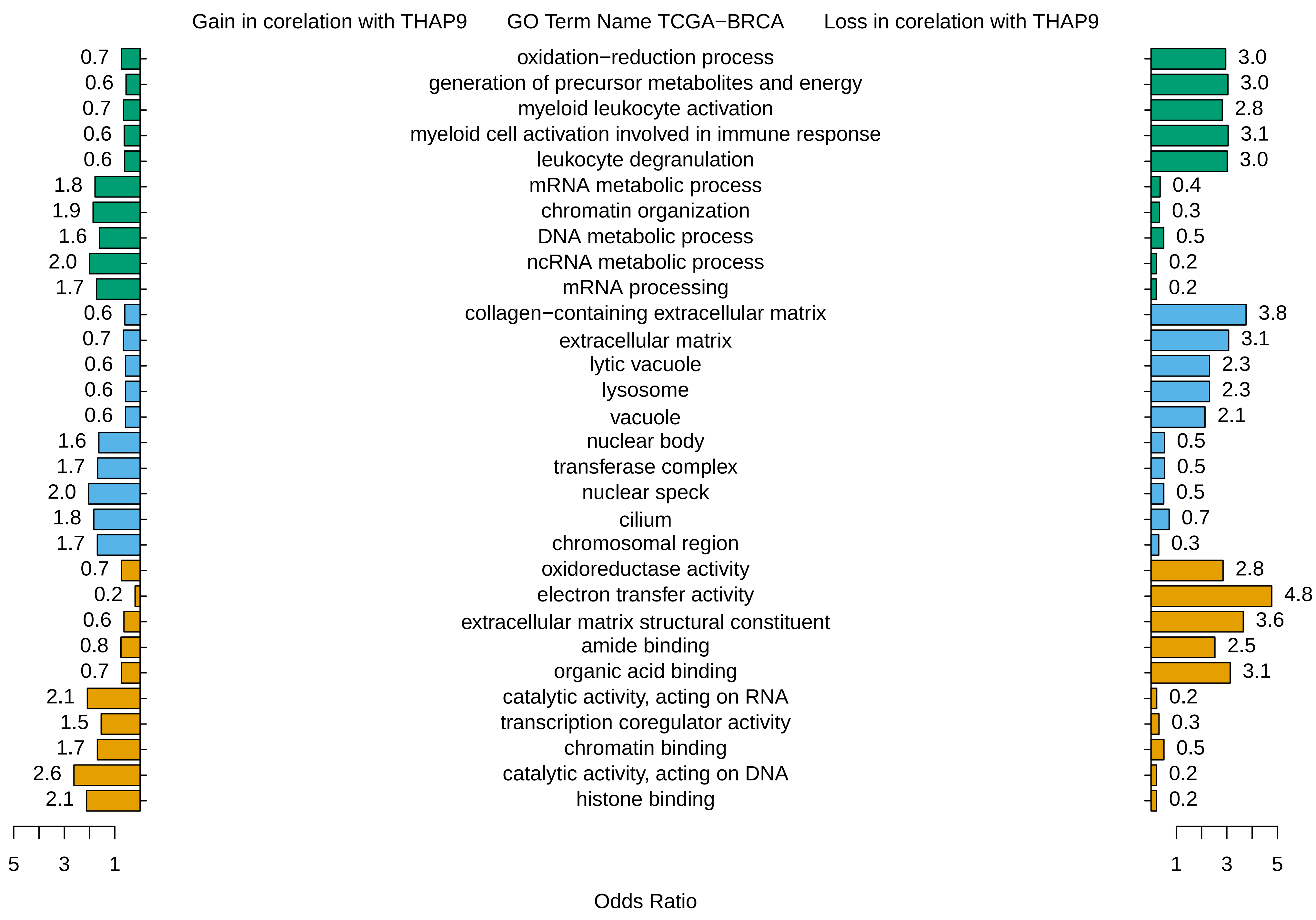
Gain in corelation with THAP9
GO Term Name TCGA−BRCA
Loss in corelation with THAP9
0.7 oxidation−reduction process 3.0
0.6 generation of precursor metabolites and energy 3.0
0.7 myeloid leukocyte activation 2.8
0.6 myeloid cell activation involved in immune response 3.1
0.6 leukocyte degranulation 3.0
1.8 mRNA metabolic process 0.4
1.9 chromatin organization 0.3
1.6 DNA metabolic process 0.5
2.0 ncRNA metabolic process 0.2
1.7 mRNA processing 0.2
0.6 collagen−containing extracellular matrix 3.8
0.7 extracellular matrix 3.1
0.6 lytic vacuole 2.3
0.6 lysosome 2.3
0.6 vacuole 2.1
1.6 nuclear body 0.5
1.7 transferase complex 0.5
2.0 nuclear speck 0.5
1.8 cilium 0.7
1.7 chromosomal region 0.3
0.7 oxidoreductase activity 2.8
0.2 electron transfer activity 4.8
0.6 extracellular matrix structural constituent 3.6
0.8 amide binding 2.5
0.7 organic acid binding 3.1
2.1 catalytic activity, acting on RNA 0.2
1.5 transcription coregulator activity 0.3
1.7 chromatin binding 0.5
2.6 catalytic activity, acting on DNA 0.2
2.1 histone binding 0.2
5 3 1
1 3 5
Odds Ratio

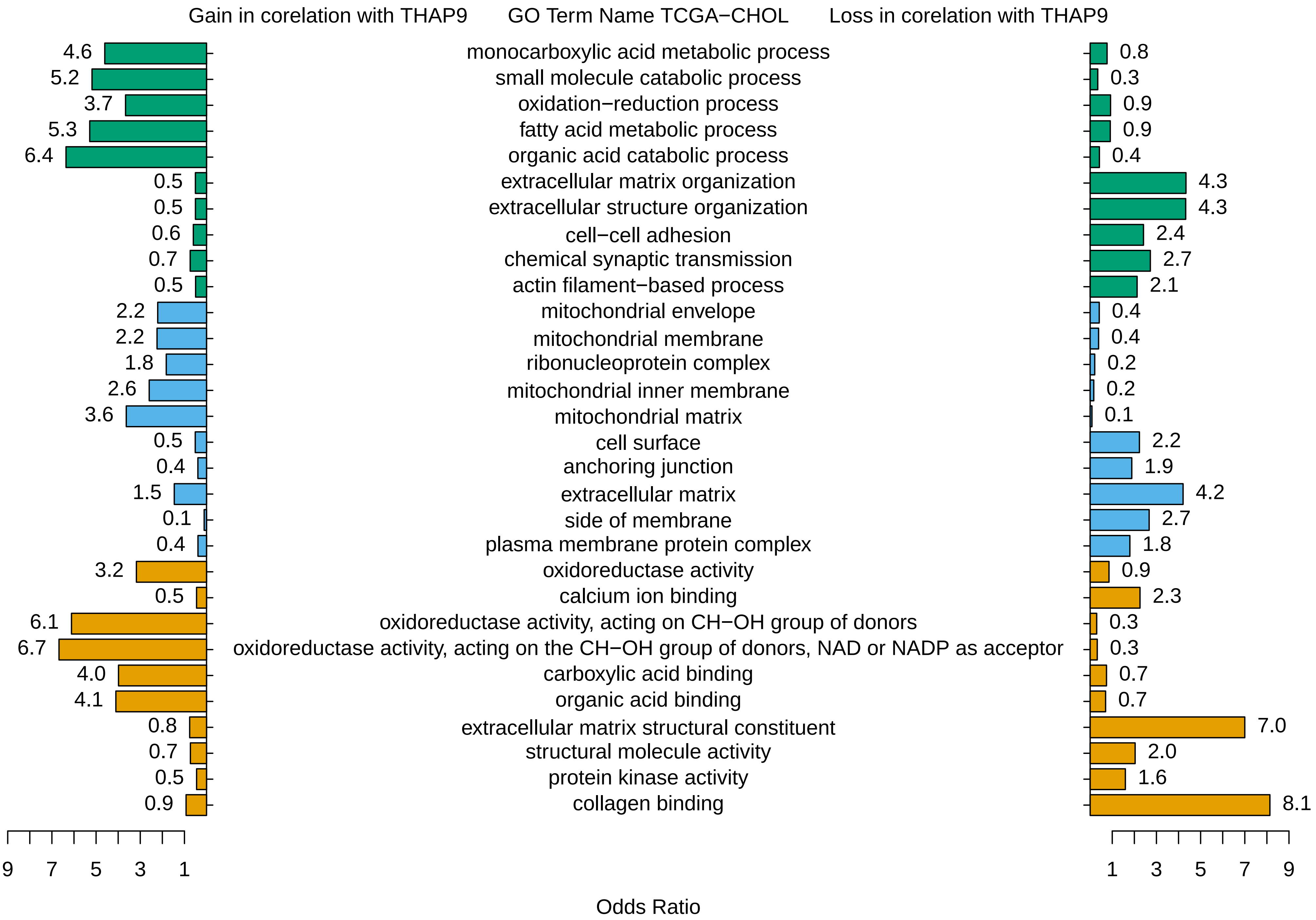

Gain in corelation with THAP9
GO Term Name TCGA−CHOL
Loss in corelation with THAP9
4.6 monocarboxylic acid metabolic process 0.8
5.2 small molecule catabolic process 0.3
3.7 oxidation−reduction process 0.9
5.3 fatty acid metabolic process 0.9
6.4 organic acid catabolic process 0.4
0.5 extracellular matrix organization 4.3
0.5 extracellular structure organization 4.3
0.6 cell−cell adhesion 2.4
0.7 chemical synaptic transmission 2.7
0.5 actin filament−based process 2.1
2.2 mitochondrial envelope 0.4
2.2 mitochondrial membrane 0.4
1.8 ribonucleoprotein complex 0.2
2.6 mitochondrial inner membrane 0.2
3.6 mitochondrial matrix 0.1
0.5 cell surface 2.2
0.4 anchoring junction 1.9
1.5 extracellular matrix 4.2
0.1 side of membrane 2.7
0.4 plasma membrane protein complex 1.8
3.2 oxidoreductase activity 0.9
0.5 calcium ion binding 2.3
6.1 oxidoreductase activity, acting on CH−OH group of donors 0.3
6.7 oxidoreductase activity, acting on the CH−OH group of donors, NAD or NADP as acceptor 0.3
4.0 carboxylic acid binding 0.7
4.1 organic acid binding 0.7
0.8 extracellular matrix structural constituent 7.0
0.7 structural molecule activity 2.0
0.5 protein kinase activity 1.6
0.9 collagen binding 8.1
9 7 5 3 1
1 3 5 7 9
Odds Ratio

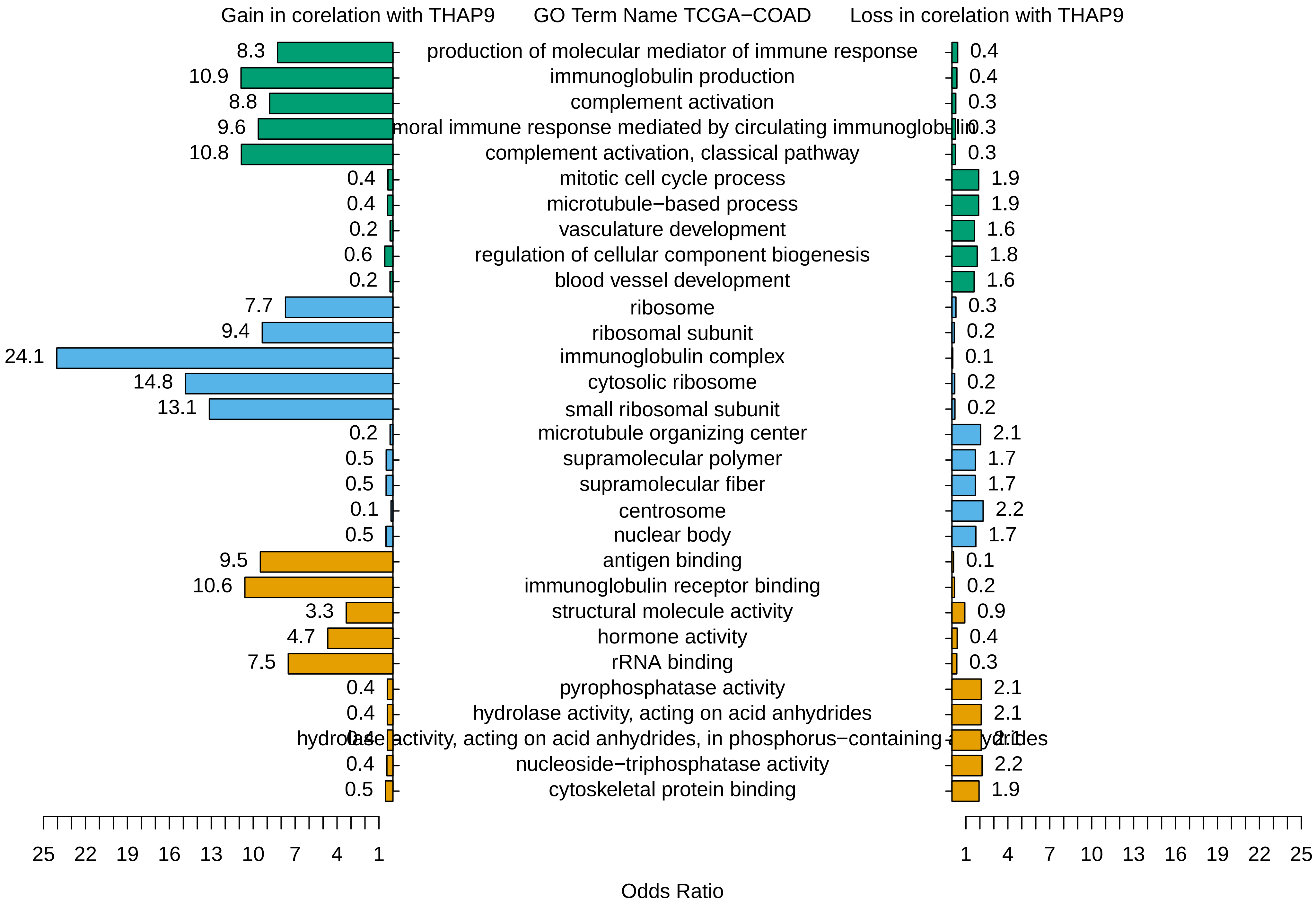
Gain in corelation with THAP9
GO Term Name TCGA−COAD
Loss in corelation with THAP9
production of molecular mediator of immune response
immunoglobulin production
complement activation
humoral immune response mediated by circulating immunoglobulin
complement activation, classical pathway
mitotic cell cycle process
microtubule−based process
vasculature development
regulation of cellular component biogenesis
blood vessel development
ribosome
ribosomal subunit
immunoglobulin complex
cytosolic ribosome
small ribosomal subunit
microtubule organizing center
supramolecular polymer
supramolecular fiber
centrosome
nuclear body
antigen binding
immunoglobulin receptor binding
structural molecule activity
hormone activity
rRNA binding
pyrophosphatase activity
hydrolase activity, acting on acid anhydrides
hydrolase activity, acting on acid anhydrides, in phosphorus−containing anhydrides
nucleoside−triphosphatase activity
cytoskeletal protein binding
8.3
10.9
8.8
9.6
10.8
0.4
0.4
0.2
0.6
0.2
7.7
9.4
24.1
14.8
13.1
0.2
0.5
0.5
0.1
0.5
9.5
10.6
3.3
4.7
7.5
0.4
0.4
0.4
0.4
0.5
0.4
0.4
0.3
0.3
0.3
1.9
1.9
1.6
1.8
1.6
0.3
0.2
0.1
0.2
0.2
2.1
1.7
1.7
2.2
1.7
0.1
0.2
0.9
0.4
0.3
2.1
2.1
2.1
2.2
1.9
25 22 19 16 13 10 7 4 1
1 4 7 10 13 16 19 22 25
Odds Ratio

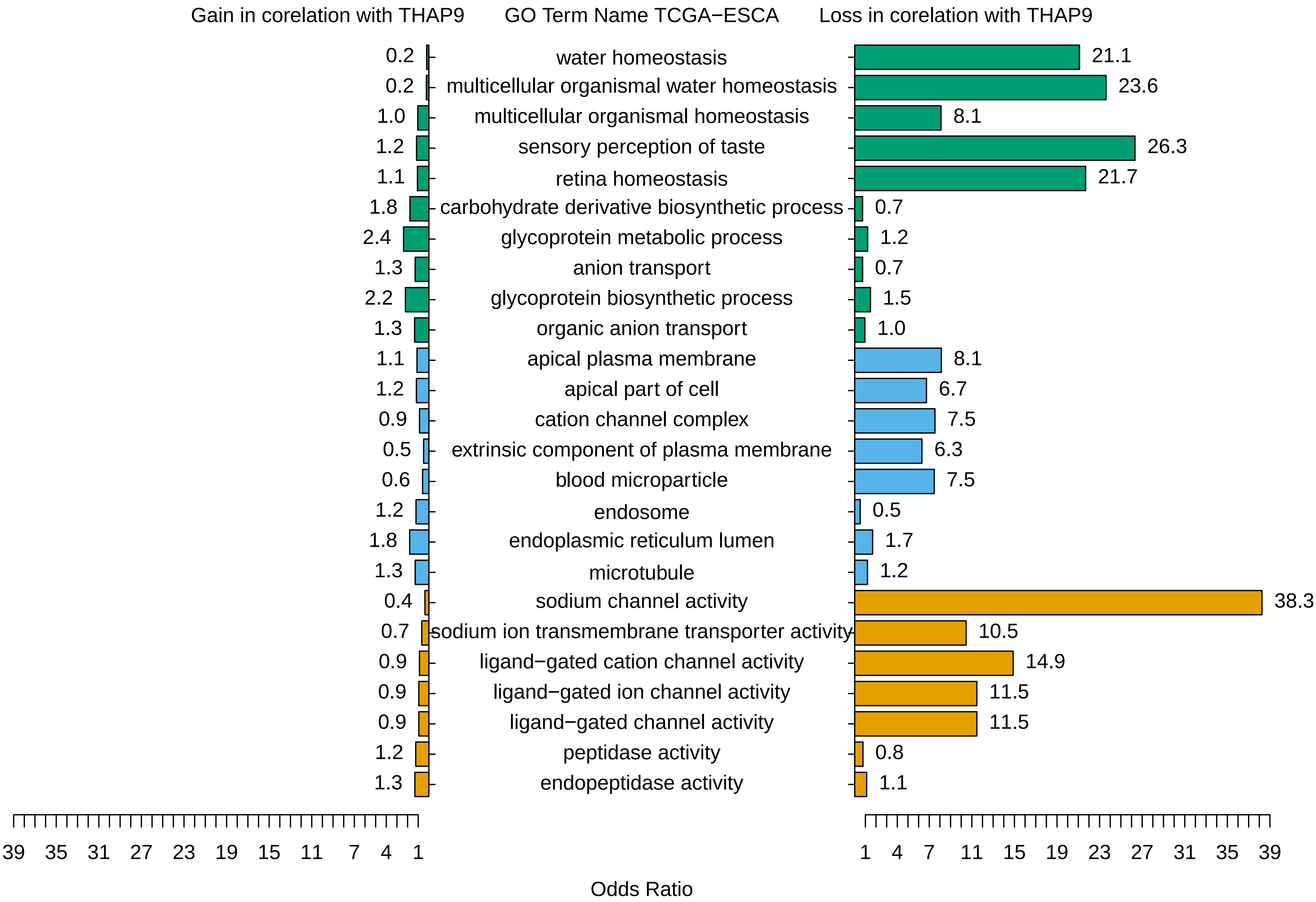

Gain in corelation with THAP9
GO Term Name TCGA−ESCA
Loss in corelation with THAP9
0.2 water homeostasis 21.1
0.2 multicellular organismal water homeostasis 23.6
1.0 multicellular organismal homeostasis 8.1
1.2 sensory perception of taste 26.3
1.1 retina homeostasis 21.7
1.8 carbohydrate derivative biosynthetic process 0.7
2.4 glycoprotein metabolic process 1.2
1.3 anion transport 0.7
2.2 glycoprotein biosynthetic process 1.5
1.3 organic anion transport 1.0
1.1 apical plasma membrane 8.1
1.2 apical part of cell 6.7
0.9 cation channel complex 7.5
0.5 extrinsic component of plasma membrane 6.3
0.6 blood microparticle 7.5
1.2 endosome 0.5
1.8 endoplasmic reticulum lumen 1.7
1.3 microtubule 1.2
0.4 sodium channel activity 38.3
0.7 sodium ion transmembrane transporter activity 10.5
0.9 ligand−gated cation channel activity 14.9
0.9 ligand−gated ion channel activity 11.5
0.9 ligand−gated channel activity 11.5
1.2 peptidase activity 0.8
1.3 endopeptidase activity 1.1
39 35 31 27 23 19 15 11 7 4 1
1 4 7 11 15 19 23 27 31 35 39
Odds Ratio

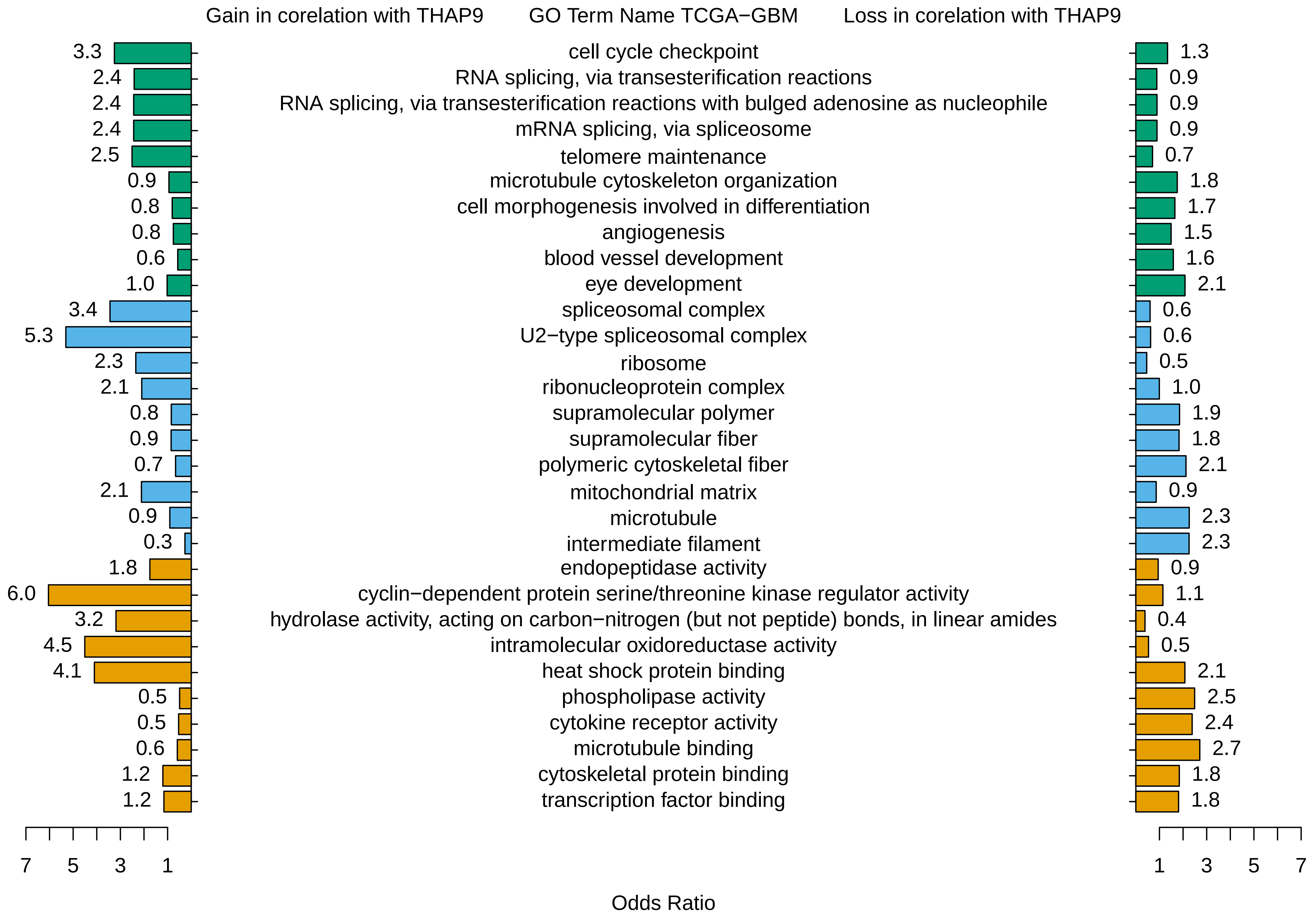

Gain in corelation with THAP9
GO Term Name TCGA−GBM
Loss in corelation with THAP9
3.3 cell cycle checkpoint 1.3
2.4 RNA splicing, via transesterification reactions 0.9
2.4 RNA splicing, via transesterification reactions with bulged adenosine as nucleophile 0.9
2.4 mRNA splicing, via spliceosome 0.9
2.5 telomere maintenance 0.7
0.9 microtubule cytoskeleton organization 1.8
0.8 cell morphogenesis involved in differentiation 1.7
0.8 angiogenesis 1.5
0.6 blood vessel development 1.6
1.0 eye development 2.1
3.4 spliceosomal complex 0.6
5.3 U2−type spliceosomal complex 0.6
2.3 ribosome 0.5
2.1 ribonucleoprotein complex 1.0
0.8 supramolecular polymer 1.9
0.9 supramolecular fiber 1.8
0.7 polymeric cytoskeletal fiber 2.1
2.1 mitochondrial matrix 0.9
0.9 microtubule 2.3
0.3 intermediate filament 2.3
1.8 endopeptidase activity 0.9
6.0 cyclin−dependent protein serine/threonine kinase regulator activity 1.1
3.2 hydrolase activity, acting on carbon−nitrogen (but not peptide) bonds, in linear amides 0.4
4.5 intramolecular oxidoreductase activity 0.5
4.1 heat shock protein binding 2.1
0.5 phospholipase activity 2.5
0.5 cytokine receptor activity 2.4
0.6 microtubule binding 2.7
1.2 cytoskeletal protein binding 1.8
1.2 transcription factor binding 1.8
7 5 3 1
1 3 5 7
Odds Ratio

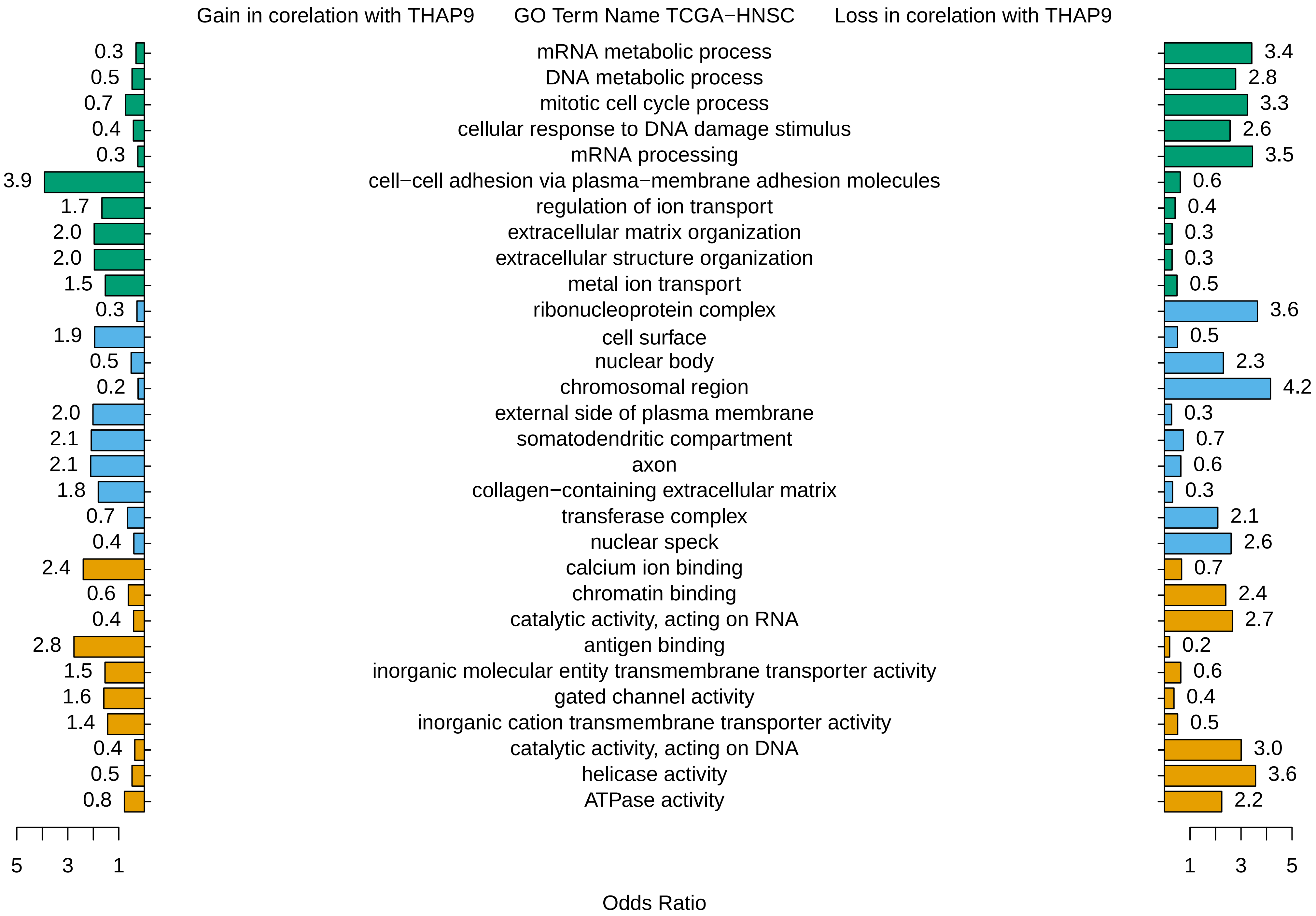

Gain in corelation with THAP9
GO Term Name TCGA−HNSC
Loss in corelation with THAP9
0.3 mRNA metabolic process 3.4
0.5 DNA metabolic process 2.8
0.7 mitotic cell cycle process 3.3
0.4 cellular response to DNA damage stimulus 2.6
0.3 mRNA processing 3.5
3.9 cell−cell adhesion via plasma−membrane adhesion molecules 0.6
1.7 regulation of ion transport 0.4
2.0 extracellular matrix organization 0.3
2.0 extracellular structure organization 0.3
1.5 metal ion transport 0.5
0.3 ribonucleoprotein complex 3.6
1.9 cell surface 0.5
0.5 nuclear body 2.3
0.2 chromosomal region 4.2
2.0 external side of plasma membrane 0.3
2.1 somatodendritic compartment 0.7
2.1 axon 0.6
1.8 collagen−containing extracellular matrix 0.3
0.7 transferase complex 2.1
0.4 nuclear speck 2.6
2.4 calcium ion binding 0.7
0.6 chromatin binding 2.4
0.4 catalytic activity, acting on RNA 2.7
2.8 antigen binding 0.2
1.5 inorganic molecular entity transmembrane transporter activity 0.6
1.6 gated channel activity 0.4
1.4 inorganic cation transmembrane transporter activity 0.5
0.4 catalytic activity, acting on DNA 3.0
0.5 helicase activity 3.6
0.8 ATPase activity 2.2
5 3 1
1 3 5
Odds Ratio

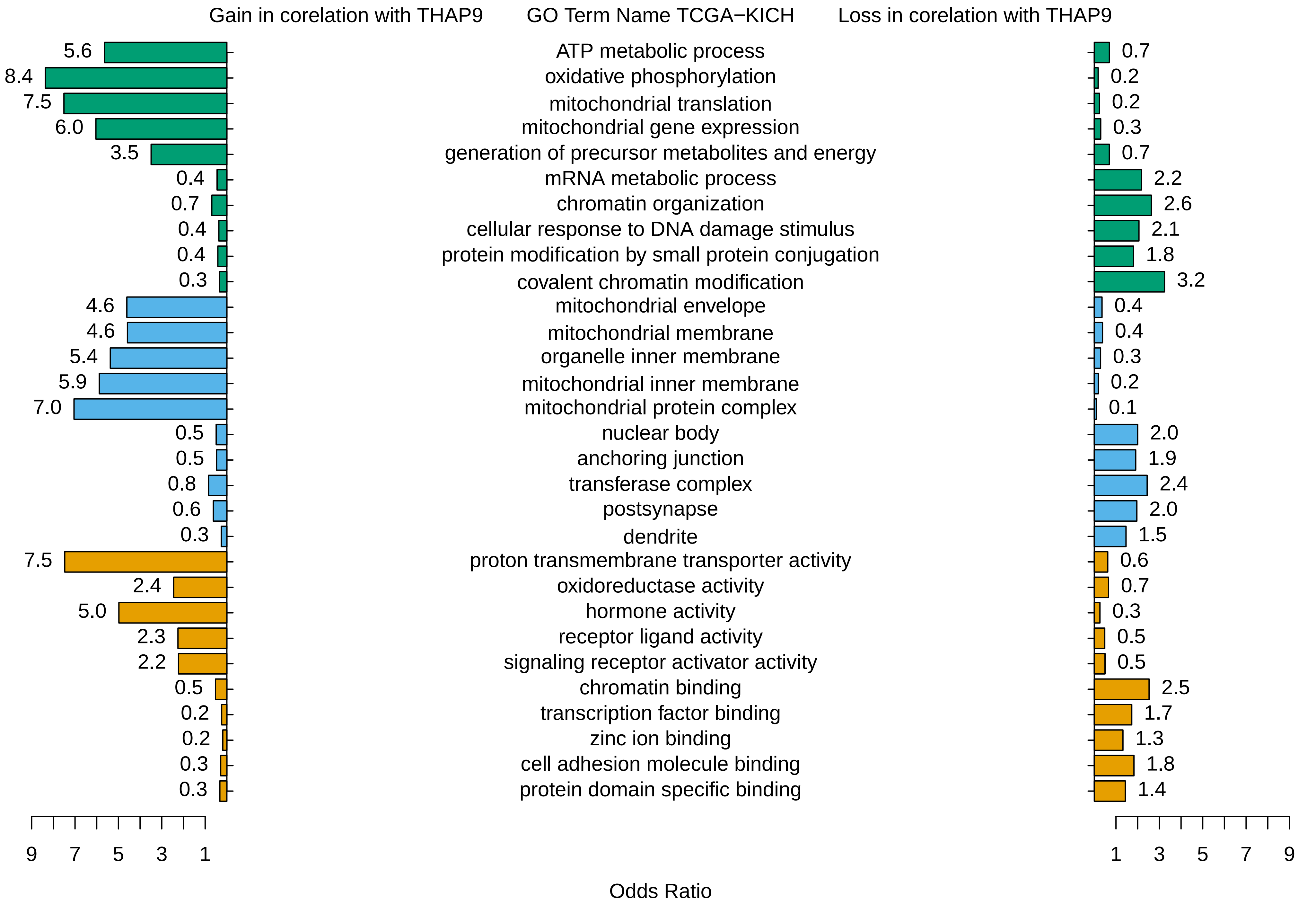
Gain in corelation with THAP9
GO Term Name TCGA−KICH
Loss in corelation with THAP9
5.6 ATP metabolic process 0.7
8.4 oxidative phosphorylation 0.2
7.5 mitochondrial translation 0.2
6.0 mitochondrial gene expression 0.3
3.5 generation of precursor metabolites and energy 0.7
0.4 mRNA metabolic process 2.2
0.7 chromatin organization 2.6
0.4 cellular response to DNA damage stimulus 2.1
0.4 protein modification by small protein conjugation 1.8
0.3 covalent chromatin modification 3.2
4.6 mitochondrial envelope 0.4
4.6 mitochondrial membrane 0.4
5.4 organelle inner membrane 0.3
5.9 mitochondrial inner membrane 0.2
7.0 mitochondrial protein complex 0.1
0.5 nuclear body 2.0
0.5 anchoring junction 1.9
0.8 transferase complex 2.4
0.6 postsynapse 2.0
0.3 dendrite 1.5
7.5 proton transmembrane transporter activity 0.6
2.4 oxidoreductase activity 0.7
5.0 hormone activity 0.3
2.3 receptor ligand activity 0.5
2.2 signaling receptor activator activity 0.5
0.5 chromatin binding 2.5
0.2 transcription factor binding 1.7
0.2 zinc ion binding 1.3
0.3 cell adhesion molecule binding 1.8
0.3 protein domain specific binding 1.4
9 7 5 3 1
1 3 5 7 9
Odds Ratio

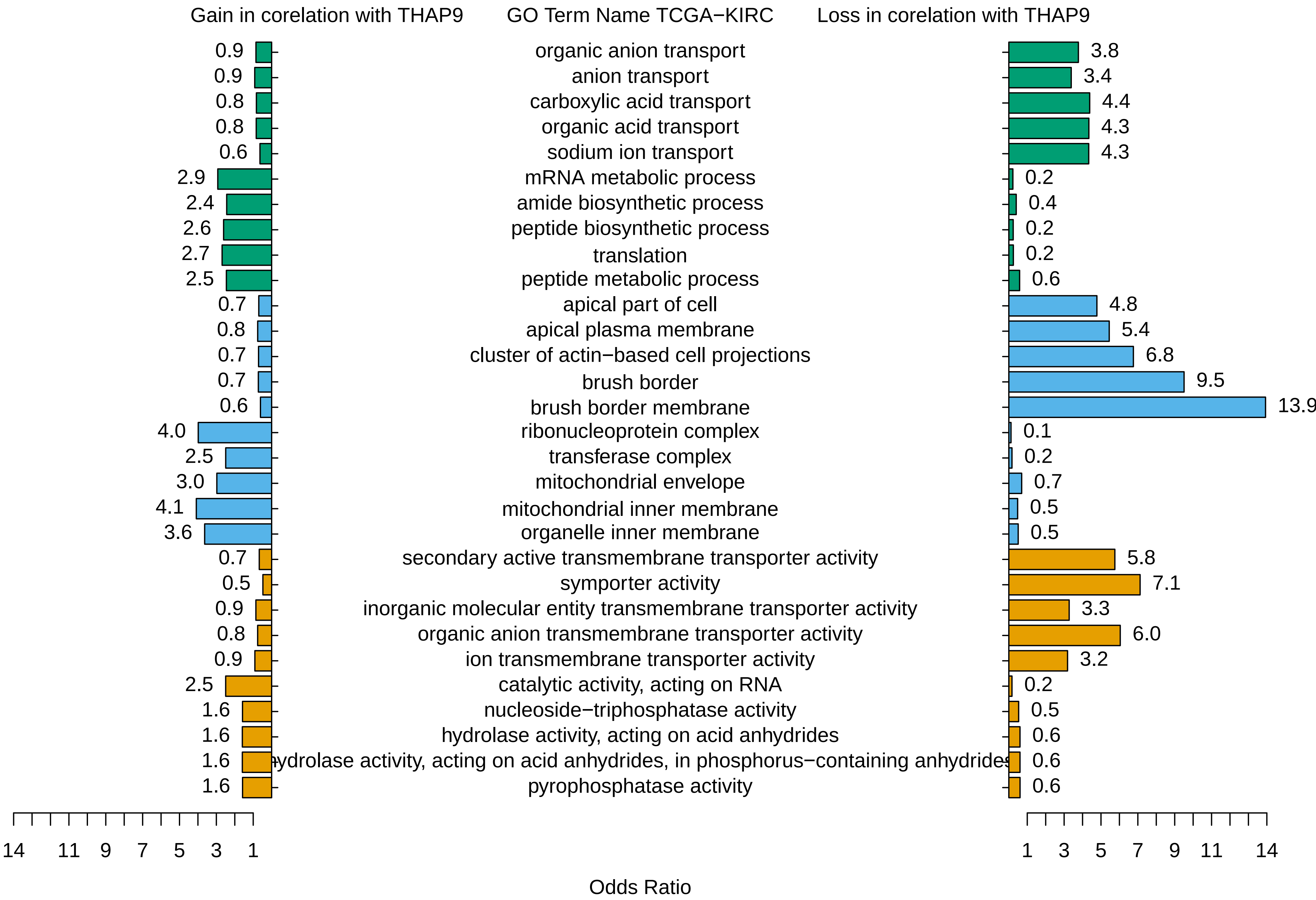

Gain in corelation with THAP9
GO Term Name TCGA−KIRC
Loss in corelation with THAP9
0.9 organic anion transport 3.8
0.9 anion transport 3.4
0.8 carboxylic acid transport 4.4
0.8 organic acid transport 4.3
0.6 sodium ion transport 4.3
2.9 mRNA metabolic process 0.2
2.4 amide biosynthetic process 0.4
2.6 peptide biosynthetic process 0.2
2.7 translation 0.2
2.5 peptide metabolic process 0.6
0.7 apical part of cell 4.8
0.8 apical plasma membrane 5.4
0.7 cluster of actin−based cell projections 6.8
0.7 brush border 9.5
0.6 brush border membrane 13.9
4.0 ribonucleoprotein complex 0.1
2.5 transferase complex 0.2
3.0 mitochondrial envelope 0.7
4.1 mitochondrial inner membrane 0.5
3.6 organelle inner membrane 0.5
0.7 secondary active transmembrane transporter activity 5.8
0.5 symporter activity 7.1
0.9 inorganic molecular entity transmembrane transporter activity 3.3
0.8 organic anion transmembrane transporter activity 6.0
0.9 ion transmembrane transporter activity 3.2
2.5 catalytic activity, acting on RNA 0.2
1.6 nucleoside−triphosphatase activity 0.5
1.6 hydrolase activity, acting on acid anhydrides 0.6
1.6 ydrolase activity, acting on acid anhydrides, in phosphorus−containing anhydride 0.6
1.6 pyrophosphatase activity 0.6
14 11 9 7 5 3 1
1 3 5 7 9 11 14
Odds Ratio

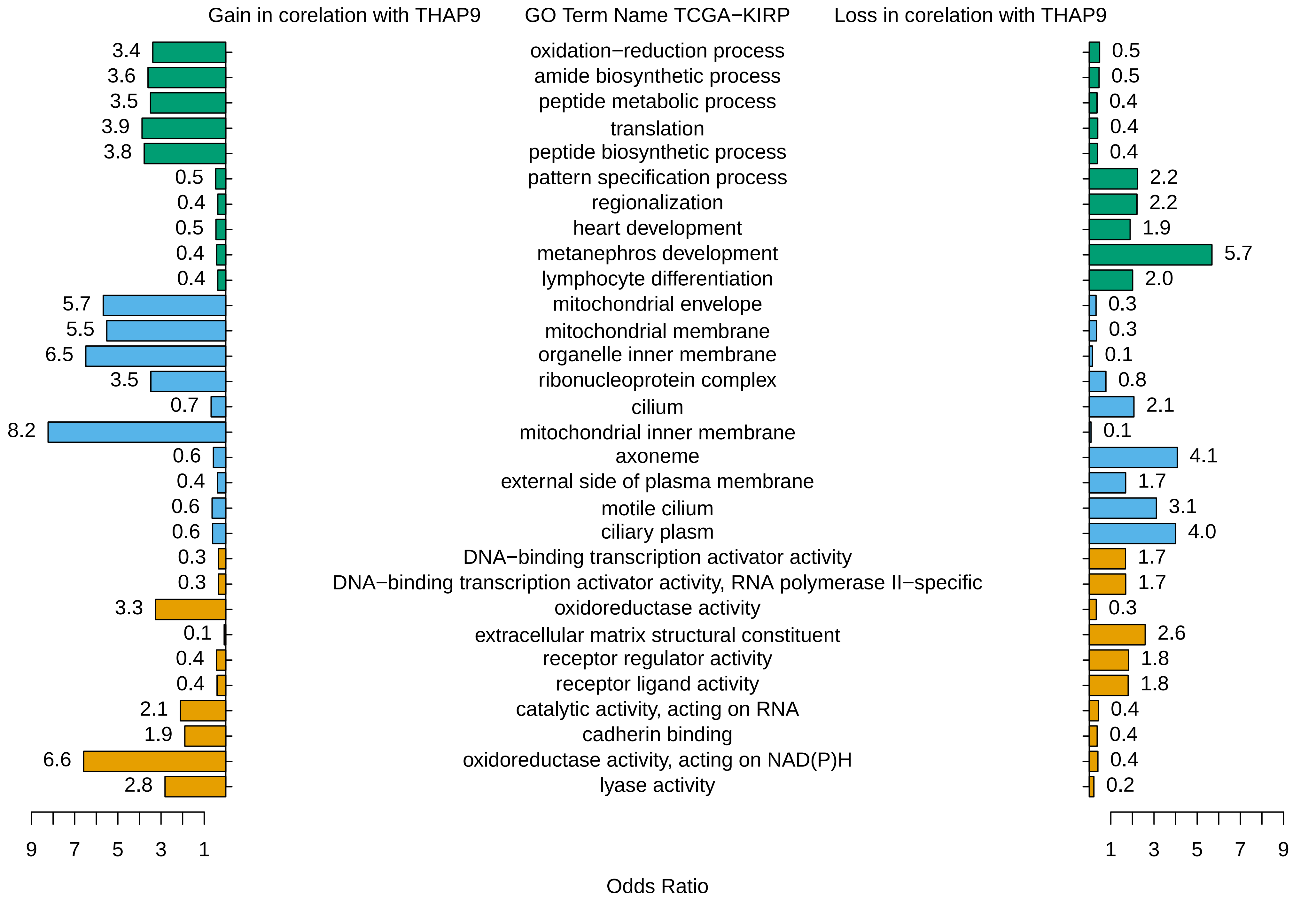
Gain in corelation with THAP9
GO Term Name TCGA−KIRP
Loss in corelation with THAP9
3.4 oxidation−reduction process 0.5
3.6 amide biosynthetic process 0.5
3.5 peptide metabolic process 0.4
3.9 translation 0.4
3.8 peptide biosynthetic process 0.4
0.5 pattern specification process 2.2
0.4 regionalization 2.2
0.5 heart development 1.9
0.4 metanephros development 5.7
0.4 lymphocyte differentiation 2.0
5.7 mitochondrial envelope 0.3
5.5 mitochondrial membrane 0.3
6.5 organelle inner membrane 0.1
3.5 ribonucleoprotein complex 0.8
0.7 cilium 2.1
8.2 mitochondrial inner membrane 0.1
0.6 axoneme 4.1
0.4 external side of plasma membrane 1.7
0.6 motile cilium 3.1
0.6 ciliary plasm 4.0
0.3 DNA−binding transcription activator activity 1.7
0.3 DNA−binding transcription activator activity, RNA polymerase II−specific 1.7
3.3 oxidoreductase activity 0.3
0.1 extracellular matrix structural constituent 2.6
0.4 receptor regulator activity 1.8
0.4 receptor ligand activity 1.8
2.1 catalytic activity, acting on RNA 0.4
1.9 cadherin binding 0.4
6.6 oxidoreductase activity, acting on NAD(P)H 0.4
2.8 lyase activity 0.2
9 7 5 3 1
1 3 5 7 9
Odds Ratio

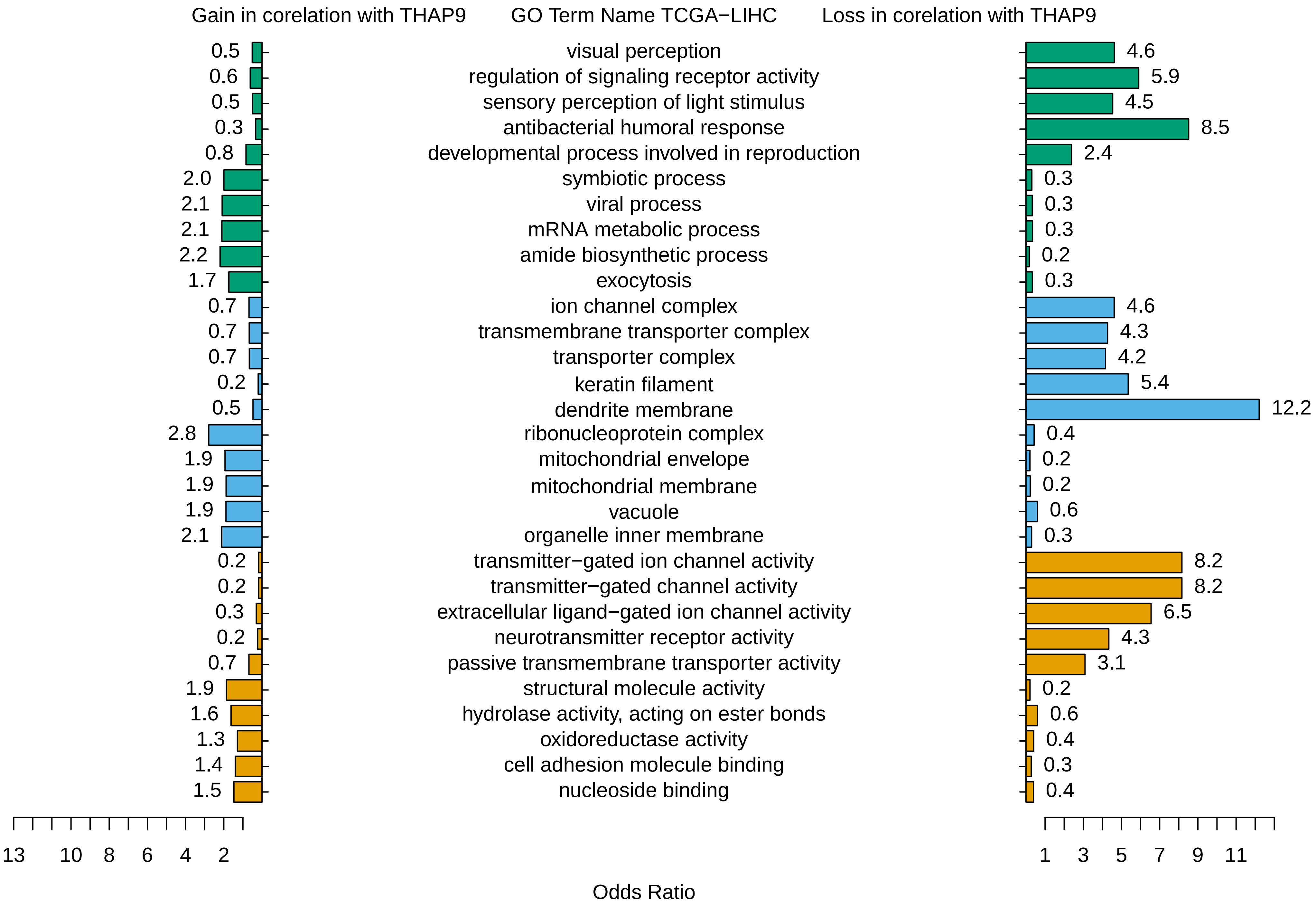
Gain in corelation with THAP9
GO Term Name TCGA−LIHC
Loss in corelation with THAP9
0.5 visual perception 4.6
0.6 regulation of signaling receptor activity 5.9
0.5 sensory perception of light stimulus 4.5
0.3 antibacterial humoral response 8.5
0.8 developmental process involved in reproduction 2.4
2.0 symbiotic process 0.3
2.1 viral process 0.3
2.1 mRNA metabolic process 0.3
2.2 amide biosynthetic process 0.2
1.7 exocytosis 0.3
0.7 ion channel complex 4.6
0.7 transmembrane transporter complex 4.3
0.7 transporter complex 4.2
0.2 keratin filament 5.4
0.5 dendrite membrane 12.2
2.8 ribonucleoprotein complex 0.4
1.9 mitochondrial envelope 0.2
1.9 mitochondrial membrane 0.2
1.9 vacuole 0.6
2.1 organelle inner membrane 0.3
0.2 transmitter−gated ion channel activity 8.2
0.2 transmitter−gated channel activity 8.2
0.3 extracellular ligand−gated ion channel activity 6.5
0.2 neurotransmitter receptor activity 4.3
0.7 passive transmembrane transporter activity 3.1
1.9 structural molecule activity 0.2
1.6 hydrolase activity, acting on ester bonds 0.6
1.3 oxidoreductase activity 0.4
1.4 cell adhesion molecule binding 0.3
1.5 nucleoside binding 0.4
13 10 8 6 4 2
1 3 5 7 9 11
Odds Ratio

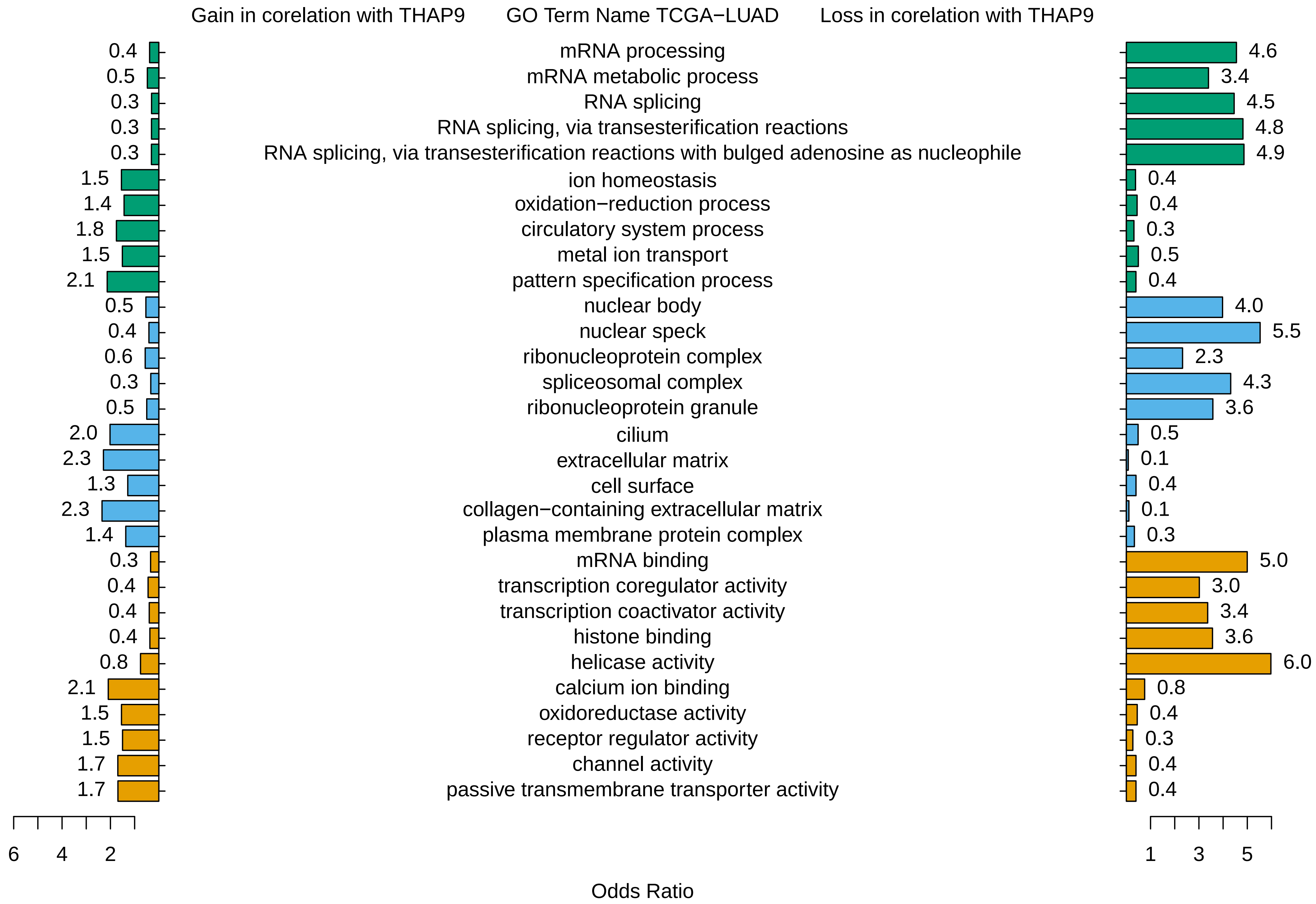
Gain in corelation with THAP9
GO Term Name TCGA−LUAD
Loss in corelation with THAP9
0.4 mRNA processing 4.6
0.5 mRNA metabolic process 3.4
0.3 RNA splicing 4.5
0.3 RNA splicing, via transesterification reactions 4.8
0.3 RNA splicing, via transesterification reactions with bulged adenosine as nucleophile 4.9
1.5 ion homeostasis 0.4
1.4 oxidation−reduction process 0.4
1.8 circulatory system process 0.3
1.5 metal ion transport 0.5
2.1 pattern specification process 0.4
0.5 nuclear body 4.0
0.4 nuclear speck 5.5
0.6 ribonucleoprotein complex 2.3
0.3 spliceosomal complex 4.3
0.5 ribonucleoprotein granule 3.6
2.0 cilium 0.5
2.3 extracellular matrix 0.1
1.3 cell surface 0.4
2.3 collagen−containing extracellular matrix 0.1
1.4 plasma membrane protein complex 0.3
0.3 mRNA binding 5.0
0.4 transcription coregulator activity 3.0
0.4 transcription coactivator activity 3.4
0.4 histone binding 3.6
0.8 helicase activity 6.0
2.1 calcium ion binding 0.8
1.5 oxidoreductase activity 0.4
1.5 receptor regulator activity 0.3
1.7 channel activity 0.4
1.7 passive transmembrane transporter activity 0.4
6 4 2
1 3 5
Odds Ratio

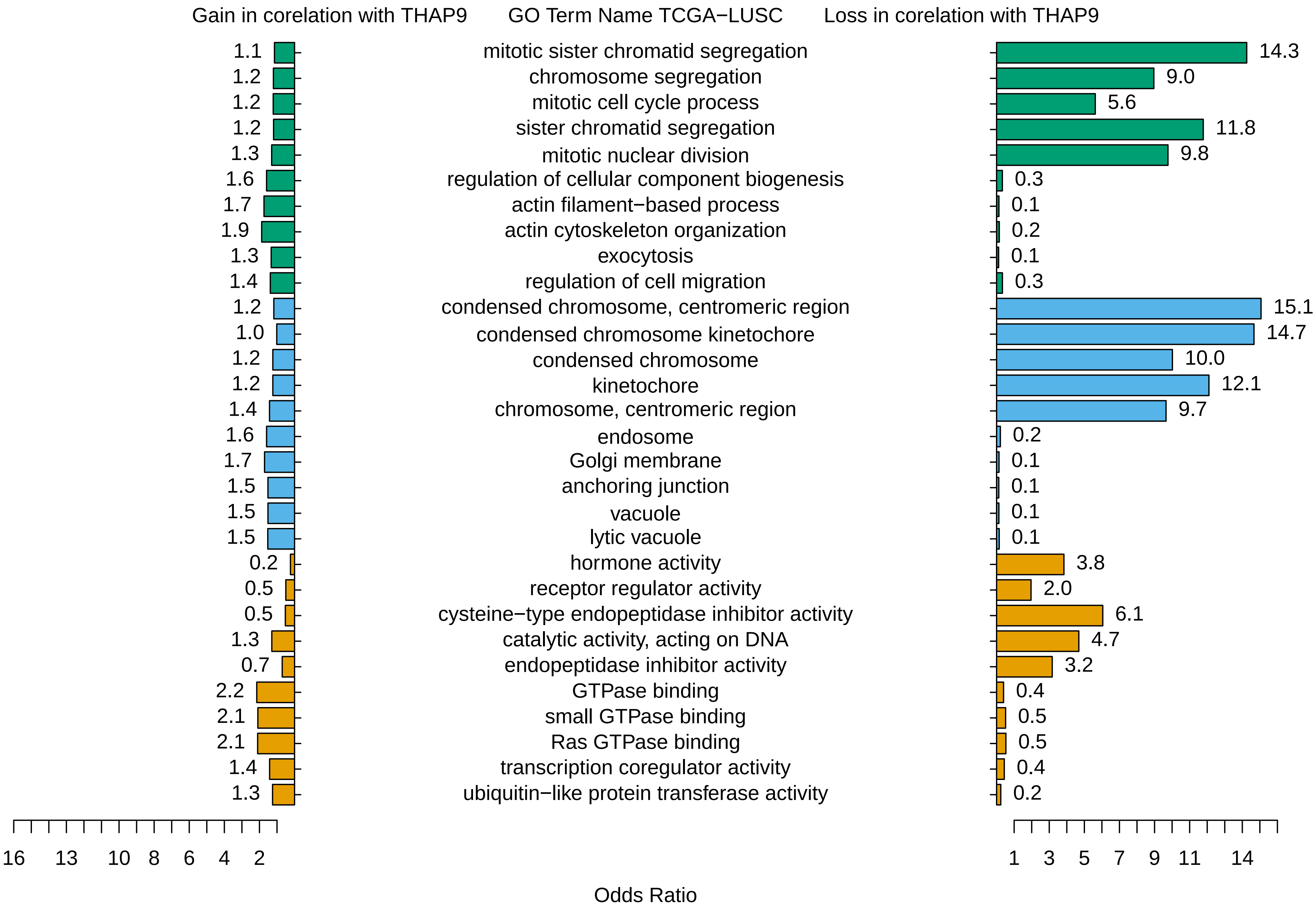

Gain in corelation with THAP9
GO Term Name TCGA−LUSC
Loss in corelation with THAP9
1.1 mitotic sister chromatid segregation 14.3
1.2 chromosome segregation 9.0
1.2 mitotic cell cycle process 5.6
1.2 sister chromatid segregation 11.8
1.3 mitotic nuclear division 9.8
1.6 regulation of cellular component biogenesis 0.3
1.7 actin filament−based process 0.1
1.9 actin cytoskeleton organization 0.2
1.3 exocytosis 0.1
1.4 regulation of cell migration 0.3
1.2 condensed chromosome, centromeric region 15.1
1.0 condensed chromosome kinetochore 14.7
1.2 condensed chromosome 10.0
1.2 kinetochore 12.1
1.4 chromosome, centromeric region 9.7
1.6 endosome 0.2
1.7 Golgi membrane 0.1
1.5 anchoring junction 0.1
1.5 vacuole 0.1
1.5 lytic vacuole 0.1
0.2 hormone activity 3.8
0.5 receptor regulator activity 2.0
0.5 cysteine−type endopeptidase inhibitor activity 6.1
1.3 catalytic activity, acting on DNA 4.7
0.7 endopeptidase inhibitor activity 3.2
2.2 GTPase binding 0.4
2.1 small GTPase binding 0.5
2.1 Ras GTPase binding 0.5
1.4 transcription coregulator activity 0.4
1.3 ubiquitin−like protein transferase activity 0.2
16 13 10 8 6 4 2
1 3 5 7 9 11 14
Odds Ratio

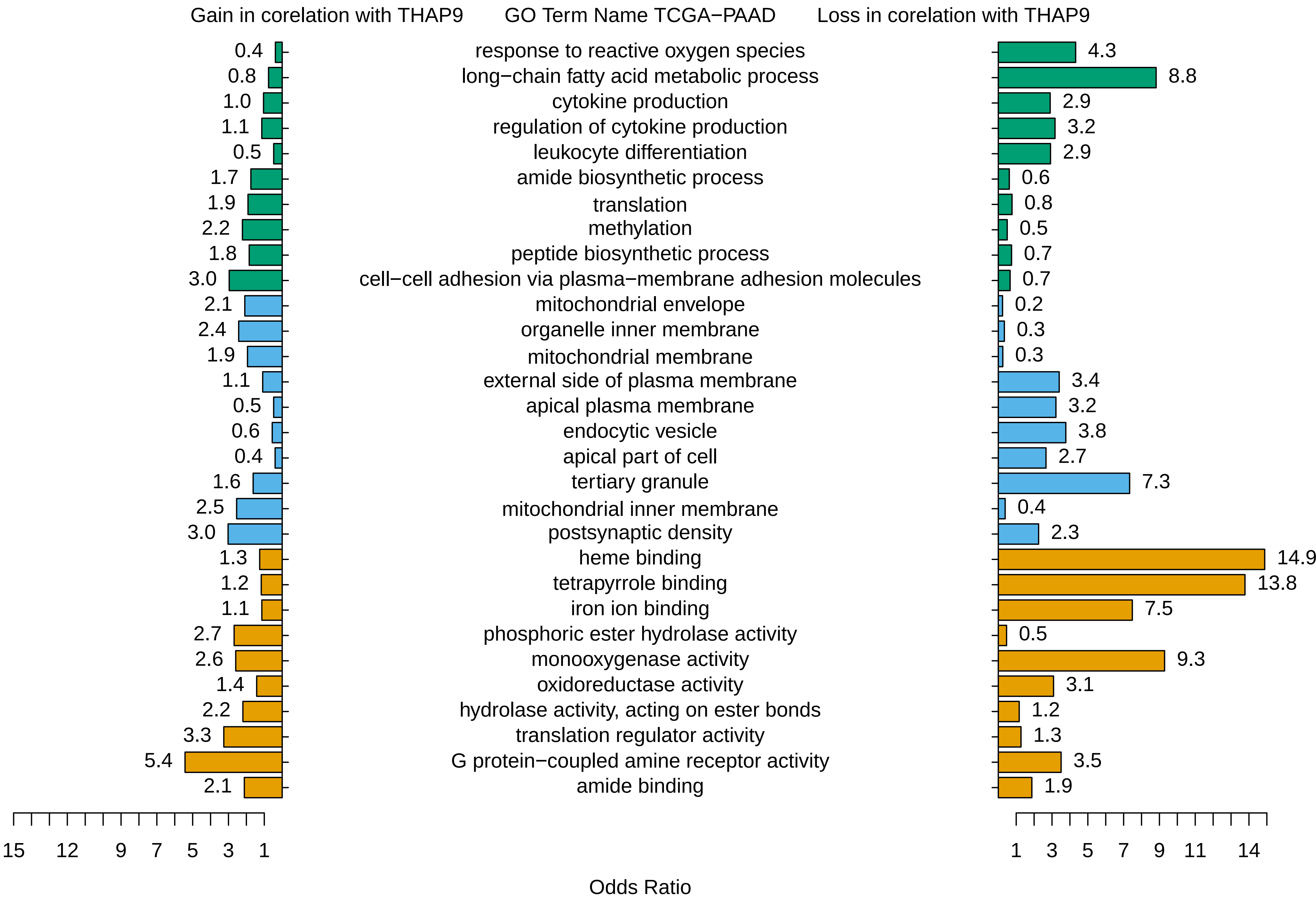

Gain in corelation with THAP9
GO Term Name TCGA−PAAD
Loss in corelation with THAP9
0.4 response to reactive oxygen species 4.3
0.8 long−chain fatty acid metabolic process 8.8
1.0 cytokine production 2.9
1.1 regulation of cytokine production 3.2
0.5 leukocyte differentiation 2.9
1.7 amide biosynthetic process 0.6
1.9 translation 0.8
2.2 methylation 0.5
1.8 peptide biosynthetic process 0.7
3.0 cell−cell adhesion via plasma−membrane adhesion molecules 0.7
2.1 mitochondrial envelope 0.2
2.4 organelle inner membrane 0.3
1.9 mitochondrial membrane 0.3
1.1 external side of plasma membrane 3.4
0.5 apical plasma membrane 3.2
0.6 endocytic vesicle 3.8
0.4 apical part of cell 2.7
1.6 tertiary granule 7.3
2.5 mitochondrial inner membrane 0.4
3.0 postsynaptic density 2.3
1.3 heme binding 14.9
1.2 tetrapyrrole binding 13.8
1.1 iron ion binding 7.5
2.7 phosphoric ester hydrolase activity 0.5
2.6 monooxygenase activity 9.3
1.4 oxidoreductase activity 3.1
2.2 hydrolase activity, acting on ester bonds 1.2
3.3 translation regulator activity 1.3
5.4 G protein−coupled amine receptor activity 3.5
2.1 amide binding 1.9
15 12 9 7 5 3 1
1 3 5 7 9 11 14
Odds Ratio

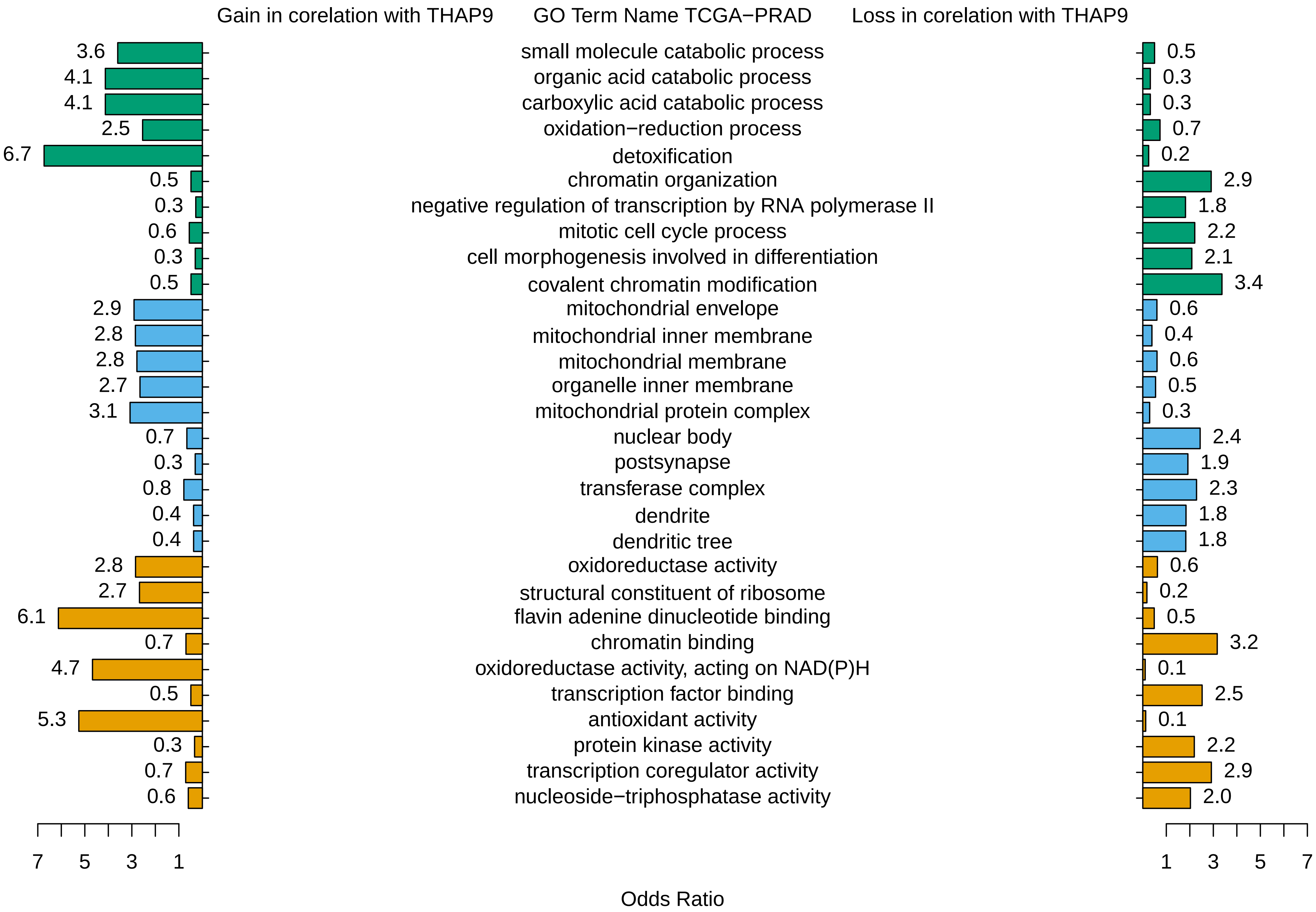

Gain in corelation with THAP9
GO Term Name TCGA−PRAD
Loss in corelation with THAP9
3.6 small molecule catabolic process 0.5
4.1 organic acid catabolic process 0.3
4.1 carboxylic acid catabolic process 0.3
2.5 oxidation−reduction process 0.7
6.7 detoxification 0.2
0.5 chromatin organization 2.9
0.3 negative regulation of transcription by RNA polymerase II 1.8
0.6 mitotic cell cycle process 2.2
0.3 cell morphogenesis involved in differentiation 2.1
0.5 covalent chromatin modification 3.4
2.9 mitochondrial envelope 0.6
2.8 mitochondrial inner membrane 0.4
2.8 mitochondrial membrane 0.6
2.7 organelle inner membrane 0.5
3.1 mitochondrial protein complex 0.3
0.7 nuclear body 2.4
0.3 postsynapse 1.9
0.8 transferase complex 2.3
0.4 dendrite 1.8
0.4 dendritic tree 1.8
2.8 oxidoreductase activity 0.6
2.7 structural constituent of ribosome 0.2
6.1 flavin adenine dinucleotide binding 0.5
0.7 chromatin binding 3.2
4.7 oxidoreductase activity, acting on NAD(P)H 0.1
0.5 transcription factor binding 2.5
5.3 antioxidant activity 0.1
0.3 protein kinase activity 2.2
0.7 transcription coregulator activity 2.9
0.6 nucleoside−triphosphatase activity 2.0
7 5 3 1
1 3 5 7
Odds Ratio

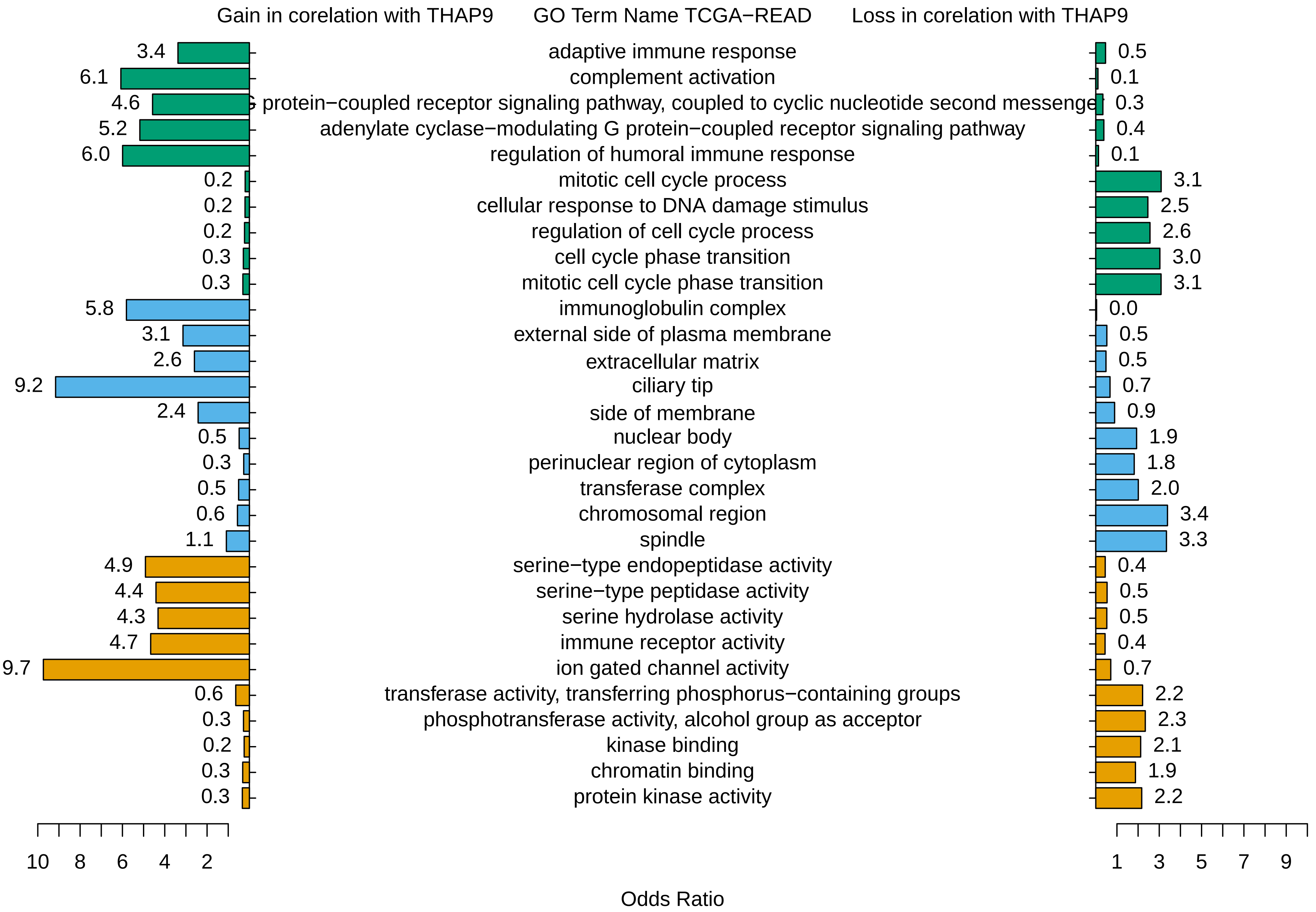
Gain in corelation with THAP9
GO Term Name TCGA−READ
Loss in corelation with THAP9
adaptive immune response
complement activation
G protein−coupled receptor signaling pathway, coupled to cyclic nucleotide second messenger
adenylate cyclase−modulating G protein−coupled receptor signaling pathway
regulation of humoral immune response
mitotic cell cycle process
cellular response to DNA damage stimulus
regulation of cell cycle process
cell cycle phase transition
mitotic cell cycle phase transition
immunoglobulin complex
external side of plasma membrane
extracellular matrix
ciliary tip
side of membrane
nuclear body
perinuclear region of cytoplasm
transferase complex
chromosomal region
spindle
serine−type endopeptidase activity
serine−type peptidase activity
serine hydrolase activity
immune receptor activity
ion gated channel activity
transferase activity, transferring phosphorus−containing groups
phosphotransferase activity, alcohol group as acceptor
kinase binding
chromatin binding
protein kinase activity
Odds Ratio

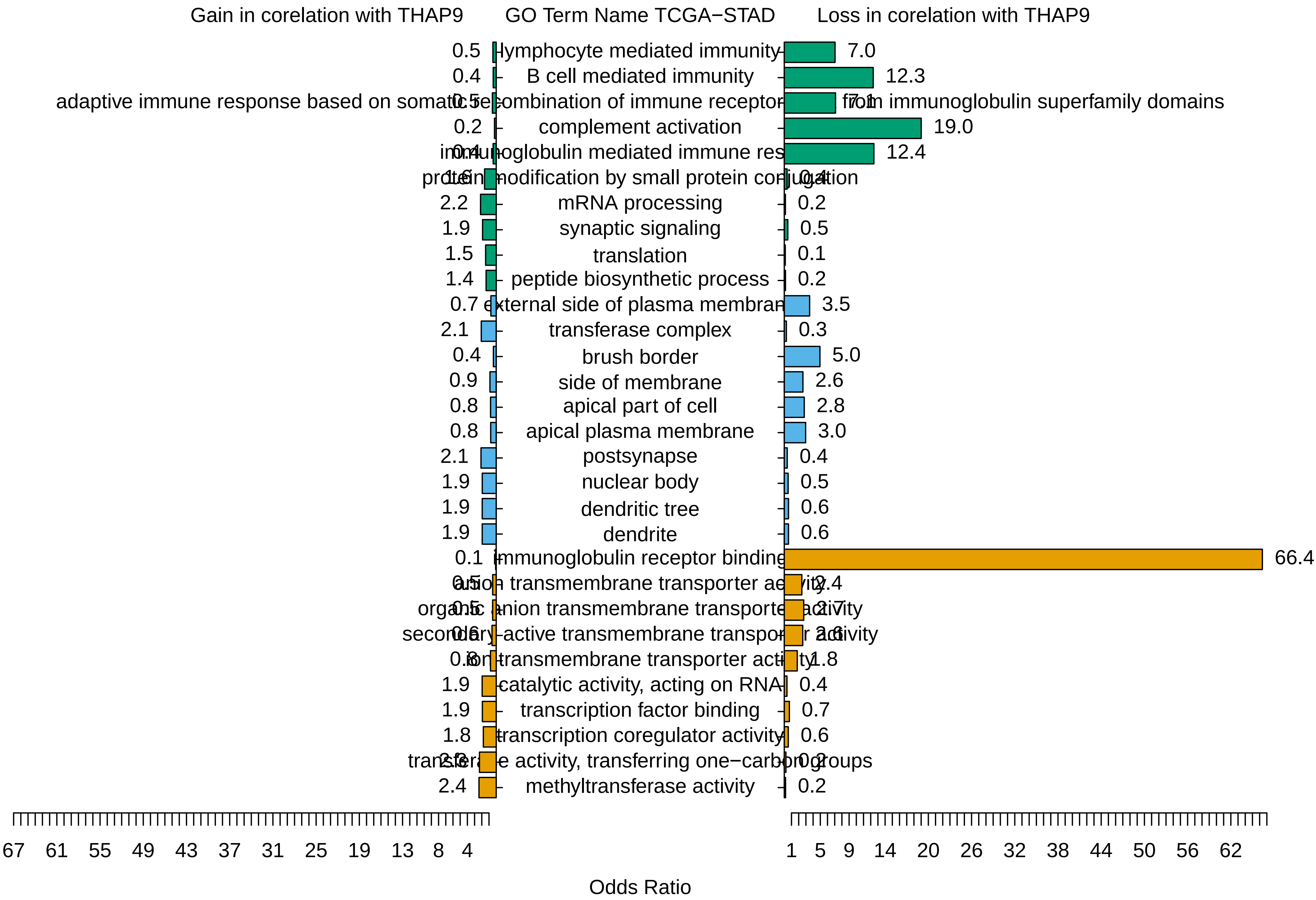

Gain in corelation with THAP9
GO Term Name TCGA−STAD
Loss in corelation with THAP9
lymphocyte mediated immunity
B cell mediated immunity
adaptive immune response based on somatic recombination of immune receptors built from immunoglobulin superfamily domains
complement activation
immunoglobulin mediated immune response
protein modification by small protein conjugation
mRNA processing
synaptic signaling
translation
peptide biosynthetic process
external side of plasma membrane
transferase complex
brush border
side of membrane
apical part of cell
apical plasma membrane
postsynapse
nuclear body
dendritic tree
dendrite
immunoglobulin receptor binding
anion transmembrane transporter activity
organic anion transmembrane transporter activity
secondary active transmembrane transporter activity
ion transmembrane transporter activity
catalytic activity, acting on RNA
transcription factor binding
transcription coregulator activity
transferase activity, transferring one−carbon groups
methyltransferase activity
0.5 0.4 0.5 0.2 0.4 1.6 2.2 1.9 1.5 1.4 0.7 2.1 0.4 0.9 0.8 0.8 2.1 1.9 1.9 1.9 0.1 0.5 0.5 0.6 0.8 1.9 1.9 1.8 2.3 2.4
7.0 12.3 7.1 19.0 12.4 0.4 0.2 0.5 0.1 0.2 3.5 0.3 5.0 2.6 2.8 3.0 0.4 0.5 0.6 0.6 66.4 2.4 2.7 2.6 1.8 0.4 0.7 0.6 0.2 0.2
67 61 55 49 43 37 31 25 19 13 8 4
1 5 9 14 20 26 32 38 44 50 56 62
Odds Ratio

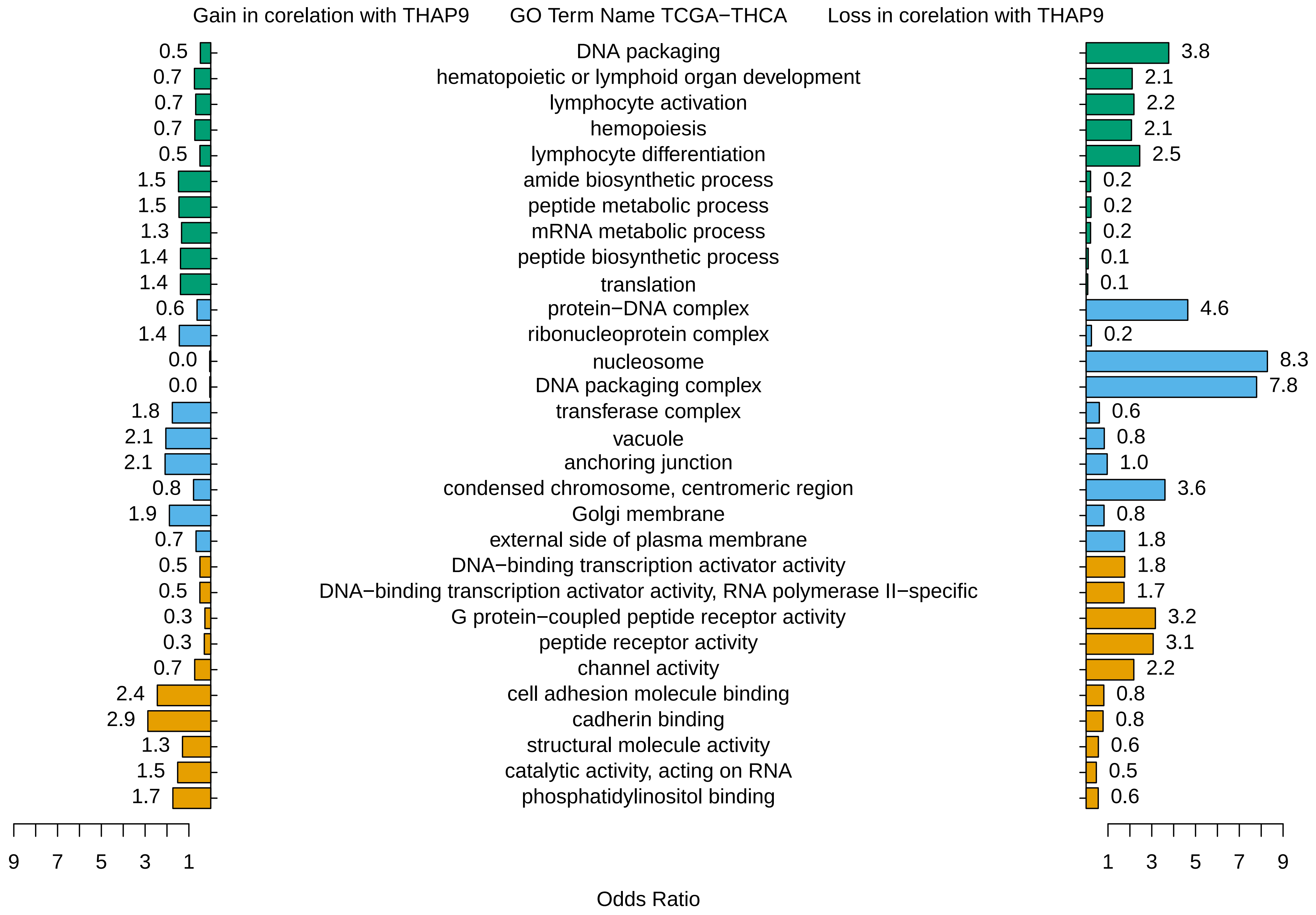
Gain in corelation with THAP9
GO Term Name TCGA−THCA
Loss in corelation with THAP9
0.5 DNA packaging 3.8
0.7 hematopoietic or lymphoid organ development 2.1
0.7 lymphocyte activation 2.2
0.7 hemopoiesis 2.1
0.5 lymphocyte differentiation 2.5
1.5 amide biosynthetic process 0.2
1.5 peptide metabolic process 0.2
1.3 mRNA metabolic process 0.2
1.4 peptide biosynthetic process 0.1
1.4 translation 0.1
0.6 protein−DNA complex 4.6
1.4 ribonucleoprotein complex 0.2
0.0 nucleosome 8.3
0.0 DNA packaging complex 7.8
1.8 transferase complex 0.6
2.1 vacuole 0.8
2.1 anchoring junction 1.0
0.8 condensed chromosome, centromeric region 3.6
1.9 Golgi membrane 0.8
0.7 external side of plasma membrane 1.8
0.5 DNA−binding transcription activator activity 1.8
0.5 DNA−binding transcription activator activity, RNA polymerase II−specific 1.7
0.3 G protein−coupled peptide receptor activity 3.2
0.3 peptide receptor activity 3.1
0.7 channel activity 2.2
2.4 cell adhesion molecule binding 0.8
2.9 cadherin binding 0.8
1.3 structural molecule activity 0.6
1.5 catalytic activity, acting on RNA 0.5
1.7 phosphatidylinositol binding 0.6
9 7 5 3 1
1 3 5 7 9
Odds Ratio

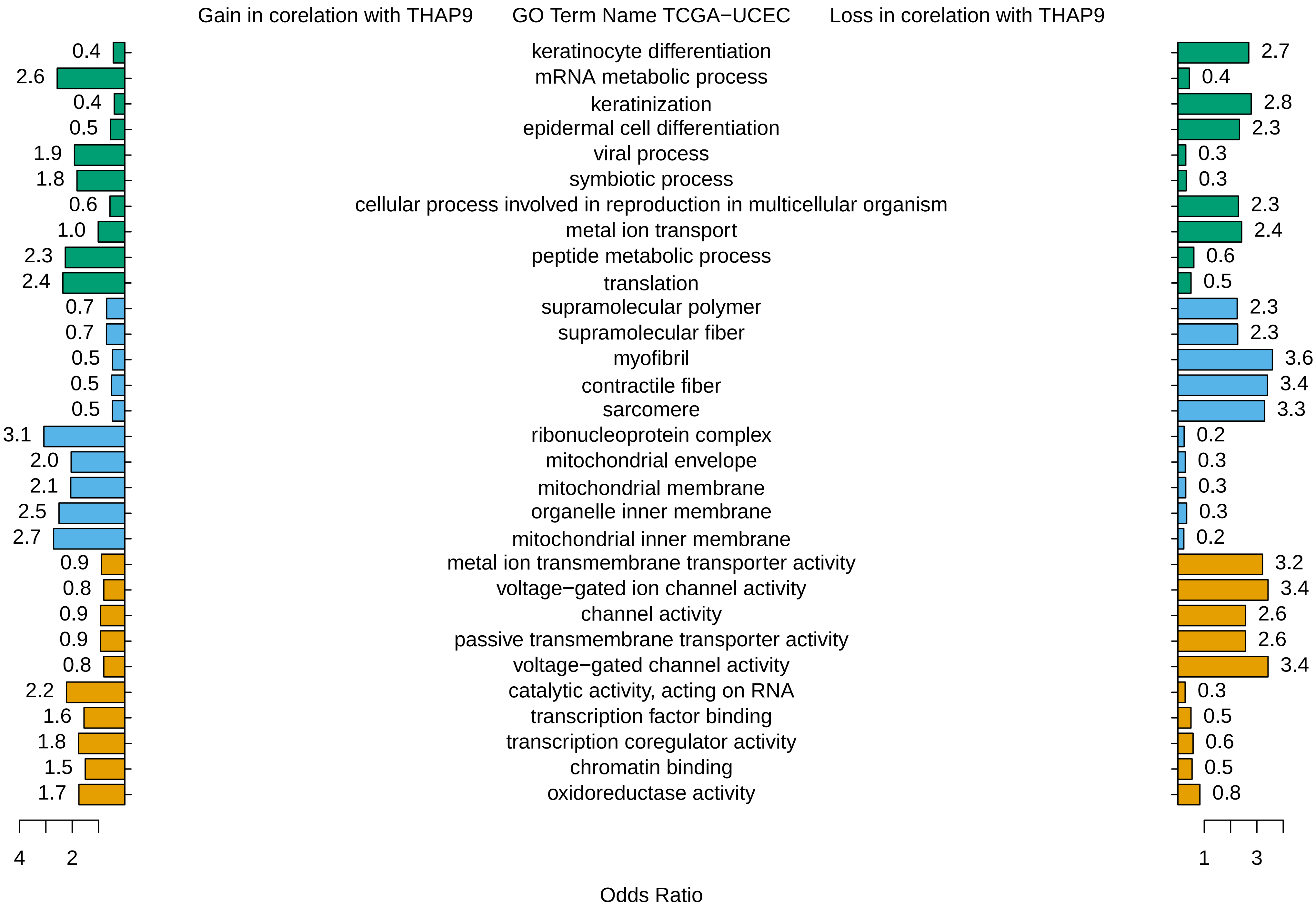

Gain in corelation with THAP9
GO Term Name TCGA−UCEC
Loss in corelation with THAP9
0.4 keratinocyte differentiation 2.7
2.6 mRNA metabolic process 0.4
0.4 keratinization 2.8
0.5 epidermal cell differentiation 2.3
1.9 viral process 0.3
1.8 symbiotic process 0.3
0.6 cellular process involved in reproduction in multicellular organism 2.3
1.0 metal ion transport 2.4
2.3 peptide metabolic process 0.6
2.4 translation 0.5
0.7 supramolecular polymer 2.3
0.7 supramolecular fiber 2.3
0.5 myofibril 3.6
0.5 contractile fiber 3.4
0.5 sarcomere 3.3
3.1 ribonucleoprotein complex 0.2
2.0 mitochondrial envelope 0.3
2.1 mitochondrial membrane 0.3
2.5 organelle inner membrane 0.3
2.7 mitochondrial inner membrane 0.2
0.9 metal ion transmembrane transporter activity 3.2
0.8 voltage−gated ion channel activity 3.4
0.9 channel activity 2.6
0.9 passive transmembrane transporter activity 2.6
0.8 voltage−gated channel activity 3.4
2.2 catalytic activity, acting on RNA 0.3
1.6 transcription factor binding 0.5
1.8 transcription coregulator activity 0.6
1.5 chromatin binding 0.5
1.7 oxidoreductase activity 0.8
4
2
1
3
Odds Ratio
