# Supplementary material for: Pan-Cancer Analysis Reveals the Prognostic Potential of the THAP9/THAP9-AS1 Sense–Antisense Gene Pair in Human Cancers
Source: Noncoding RNA. 2022 Jul 8;8(4):51. doi: 10.3390/ncrna8040051 (PMC9326536; doi:10.3390/ncrna8040051)
Supplement: Supplementary file 1 [file ncrna-08-00051-s001.zip › Supplementary Figure S6.pdf]

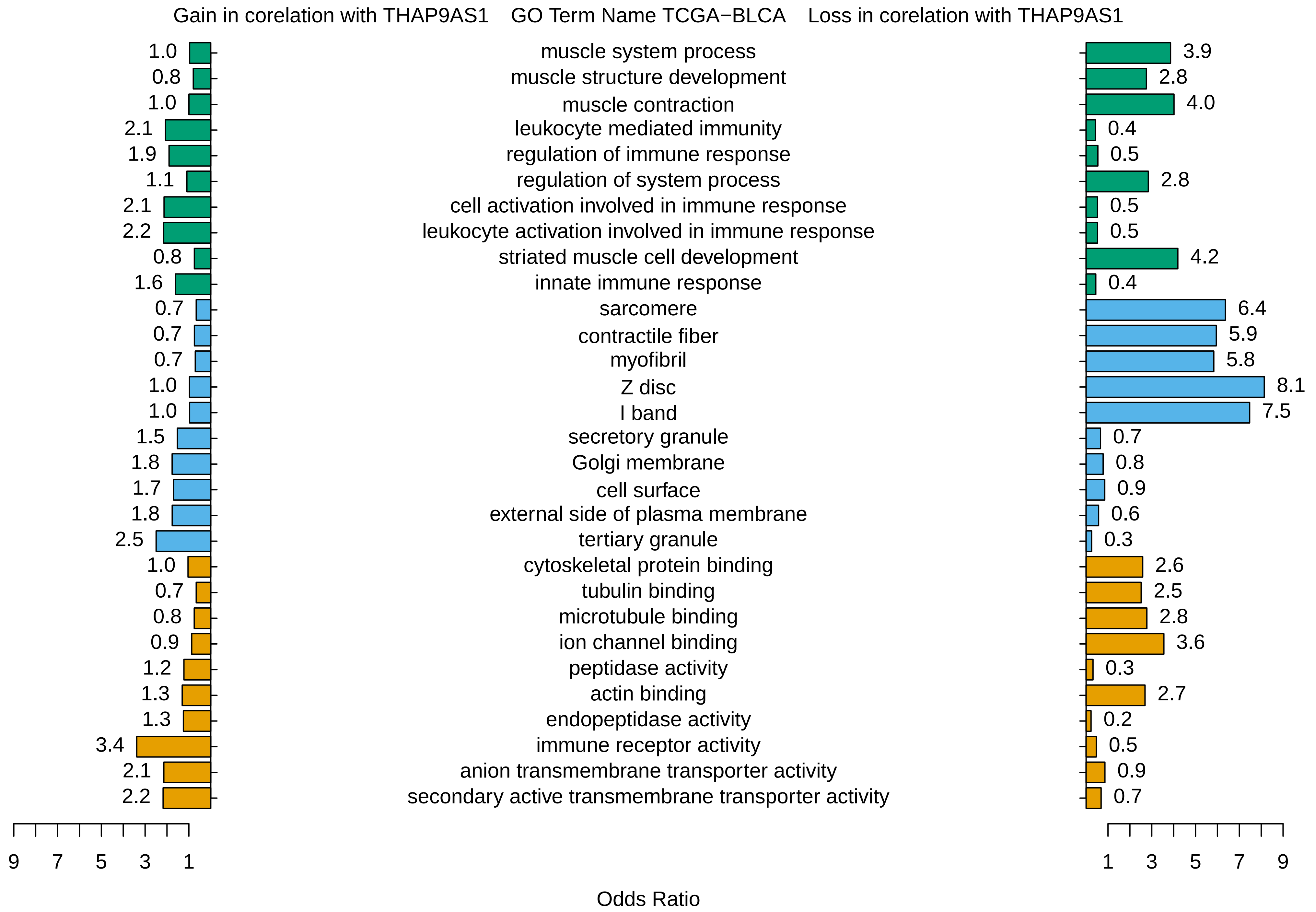

Gain in corelation with THAP9AS1
GO Term Name TCGA−BLCA
Loss in corelation with THAP9AS1
muscle system process 1.0 3.9
muscle structure development 0.8 2.8
muscle contraction 1.0 4.0
leukocyte mediated immunity 2.1 0.4
regulation of immune response 1.9 0.5
regulation of system process 1.1 2.8
cell activation involved in immune response 2.1 0.5
leukocyte activation involved in immune response 2.2 0.5
striated muscle cell development 0.8 4.2
innate immune response 1.6 0.4
sarcomere 0.7 6.4
contractile fiber 0.7 5.9
myofibril 0.7 5.8
Z disc 1.0 8.1
I band 1.0 7.5
secretory granule 1.5 0.7
Golgi membrane 1.8 0.8
cell surface 1.7 0.9
external side of plasma membrane 1.8 0.6
tertiary granule 2.5 0.3
cytoskeletal protein binding 1.0 2.6
tubulin binding 0.7 2.5
microtubule binding 0.8 2.8
ion channel binding 0.9 3.6
peptidase activity 1.2 0.3
actin binding 1.3 2.7
endopeptidase activity 1.3 0.2
immune receptor activity 3.4 0.5
anion transmembrane transporter activity 2.1 0.9
secondary active transmembrane transporter activity 2.2 0.7
9 7 5 3 1
1 3 5 7 9
Odds Ratio

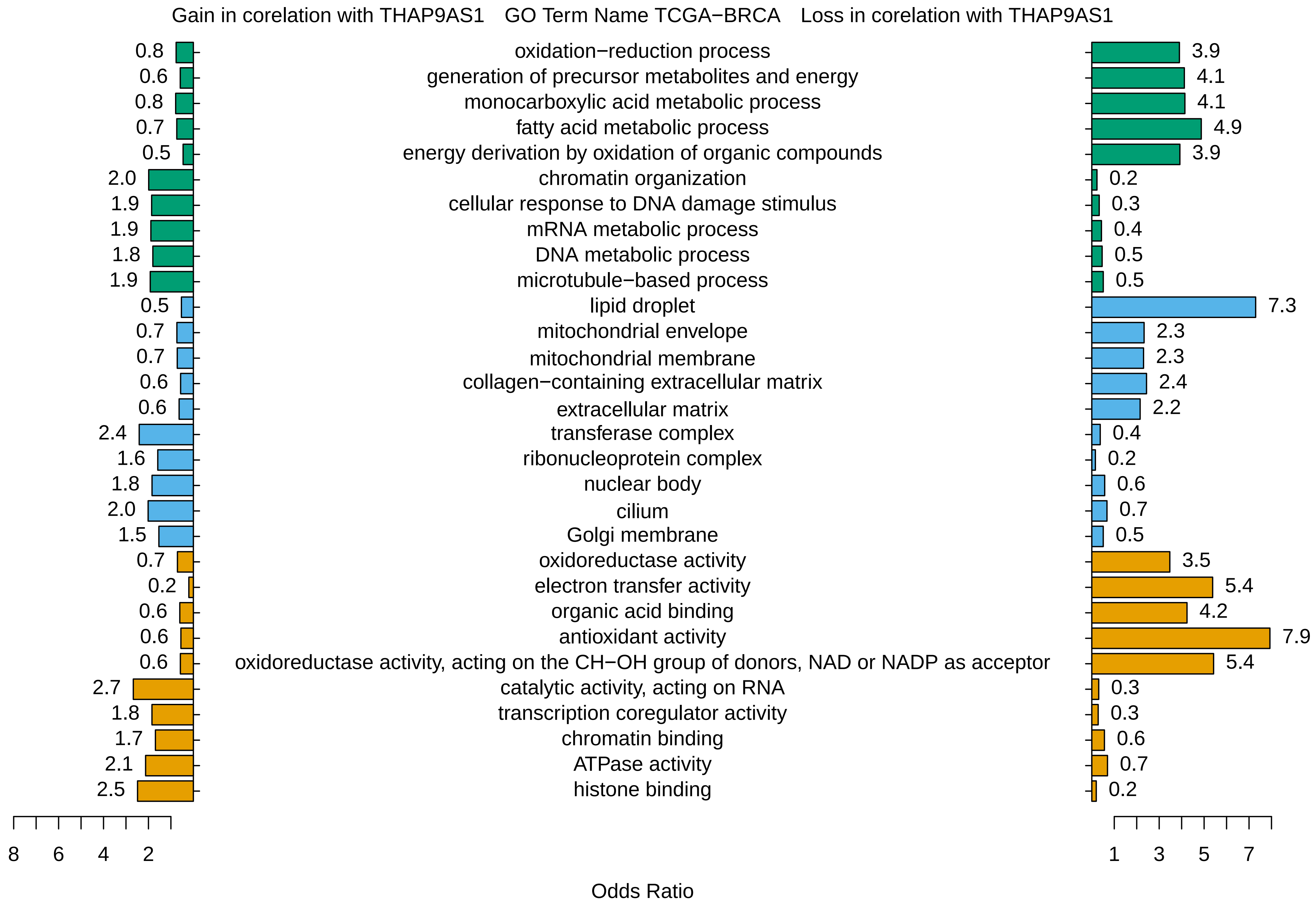
Gain in corelation with THAP9AS1
GO Term Name TCGA−BRCA
Loss in corelation with THAP9AS1
0.8 oxidation−reduction process 3.9
0.6 generation of precursor metabolites and energy 4.1
0.8 monocarboxylic acid metabolic process 4.1
0.7 fatty acid metabolic process 4.9
0.5 energy derivation by oxidation of organic compounds 3.9
2.0 chromatin organization 0.2
1.9 cellular response to DNA damage stimulus 0.3
1.9 mRNA metabolic process 0.4
1.8 DNA metabolic process 0.5
1.9 microtubule−based process 0.5
0.5 lipid droplet 7.3
0.7 mitochondrial envelope 2.3
0.7 mitochondrial membrane 2.3
0.6 collagen−containing extracellular matrix 2.4
0.6 extracellular matrix 2.2
2.4 transferase complex 0.4
1.6 ribonucleoprotein complex 0.2
1.8 nuclear body 0.6
2.0 cilium 0.7
1.5 Golgi membrane 0.5
0.7 oxidoreductase activity 3.5
0.2 electron transfer activity 5.4
0.6 organic acid binding 4.2
0.6 antioxidant activity 7.9
0.6 oxidoreductase activity, acting on the CH−OH group of donors, NAD or NADP as acceptor 5.4
2.7 catalytic activity, acting on RNA 0.3
1.8 transcription coregulator activity 0.3
1.7 chromatin binding 0.6
2.1 ATPase activity 0.7
2.5 histone binding 0.2
8 6 4 2
1 3 5 7
Odds Ratio

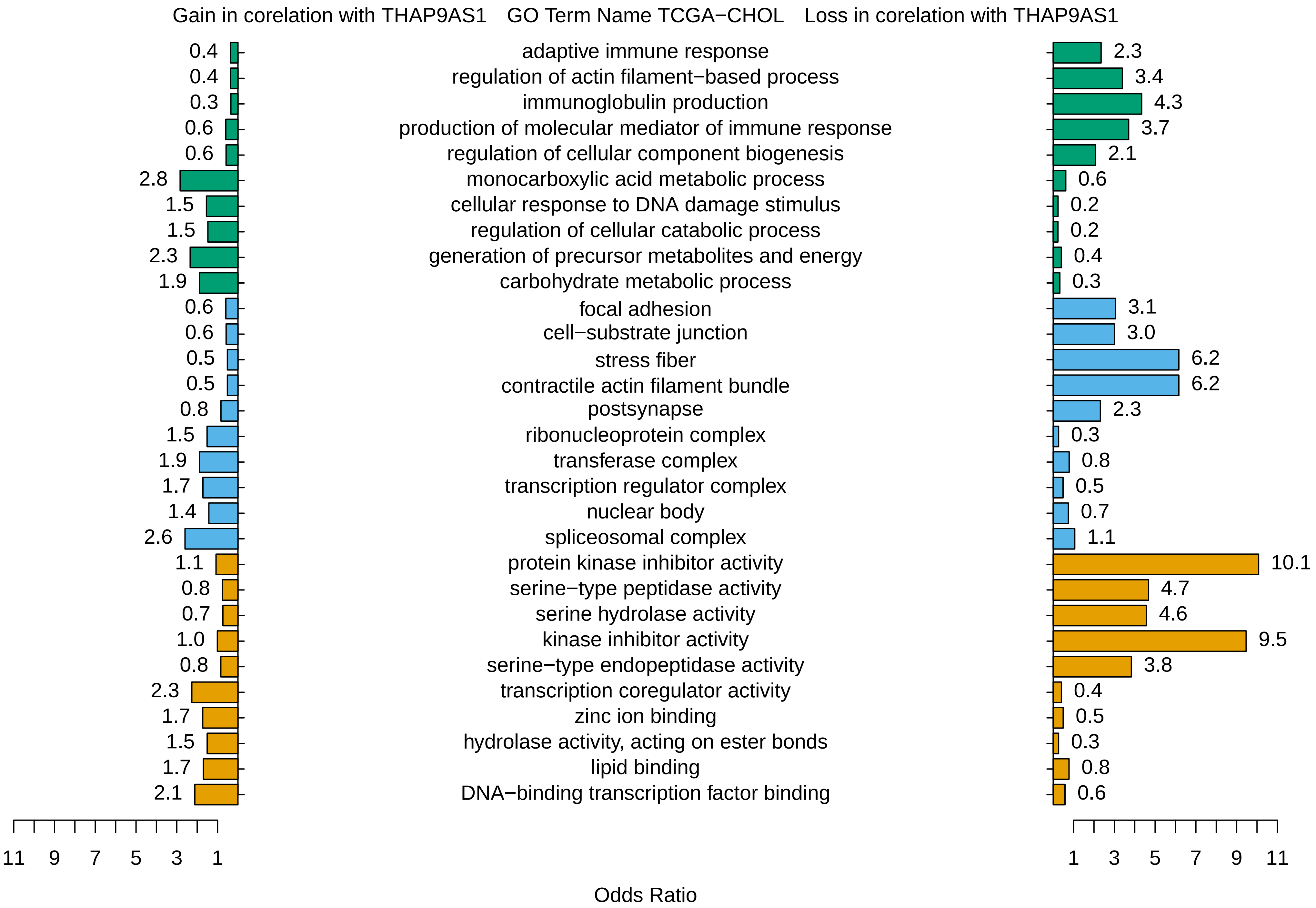

Gain in corelation with THAP9AS1
GO Term Name TCGA−CHOL
Loss in corelation with THAP9AS1
adaptive immune response 0.4 2.3
regulation of actin filament−based process 0.4 3.4
immunoglobulin production 0.3 4.3
production of molecular mediator of immune response 0.6 3.7
regulation of cellular component biogenesis 0.6 2.1
monocarboxylic acid metabolic process 2.8 0.6
cellular response to DNA damage stimulus 1.5 0.2
regulation of cellular catabolic process 1.5 0.2
generation of precursor metabolites and energy 2.3 0.4
carbohydrate metabolic process 1.9 0.3
focal adhesion 0.6 3.1
cell−substrate junction 0.6 3.0
stress fiber 0.5 6.2
contractile actin filament bundle 0.5 6.2
postsynapse 0.8 2.3
ribonucleoprotein complex 1.5 0.3
transferase complex 1.9 0.8
transcription regulator complex 1.7 0.5
nuclear body 1.4 0.7
spliceosomal complex 2.6 1.1
protein kinase inhibitor activity 1.1 10.1
serine−type peptidase activity 0.8 4.7
serine hydrolase activity 0.7 4.6
kinase inhibitor activity 1.0 9.5
serine−type endopeptidase activity 0.8 3.8
transcription coregulator activity 2.3 0.4
zinc ion binding 1.7 0.5
hydrolase activity, acting on ester bonds 1.5 0.3
lipid binding 1.7 0.8
DNA−binding transcription factor binding 2.1 0.6
11 9 7 5 3 1
1 3 5 7 9 11
Odds Ratio

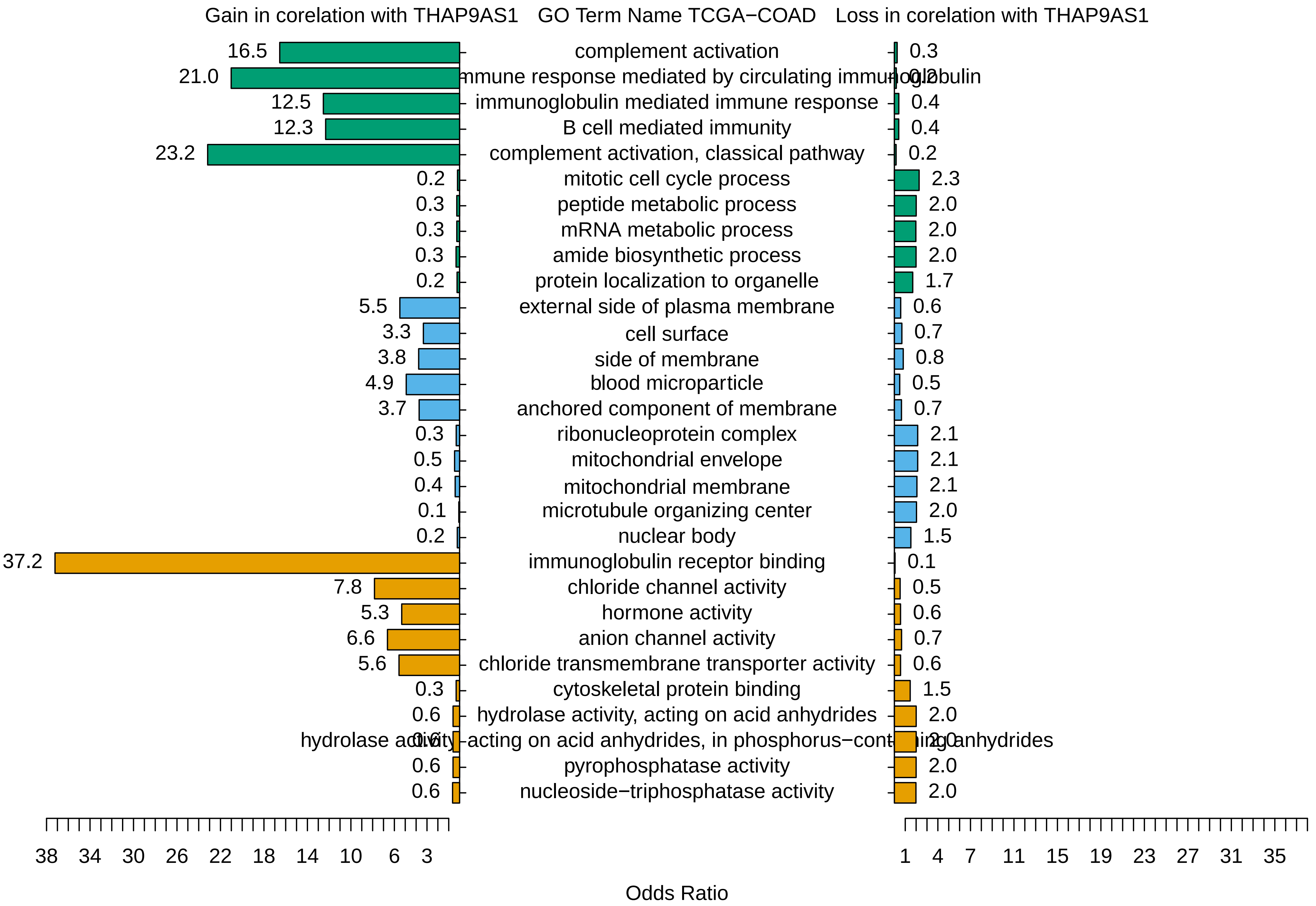

Gain in corelation with THAP9AS1
GO Term Name TCGA−COAD
Loss in corelation with THAP9AS1
complement activation
immune response mediated by circulating immunoglobulin
immunoglobulin mediated immune response
B cell mediated immunity
complement activation, classical pathway
mitotic cell cycle process
peptide metabolic process
mRNA metabolic process
amide biosynthetic process
protein localization to organelle
external side of plasma membrane
cell surface
side of membrane
blood microparticle
anchored component of membrane
ribonucleoprotein complex
mitochondrial envelope
mitochondrial membrane
microtubule organizing center
nuclear body
immunoglobulin receptor binding
chloride channel activity
hormone activity
anion channel activity
chloride transmembrane transporter activity
cytoskeletal protein binding
hydrolase activity, acting on acid anhydrides
hydrolase activity, acting on acid anhydrides, in phosphorus−containing anhydrides
pyrophosphatase activity
nucleoside−triphosphatase activity
16.5 21.0 12.5 12.3 23.2 0.2 0.3 0.3 0.3 0.2 5.5 3.3 3.8 4.9 3.7 0.3 0.5 0.4 0.1 0.2 37.2 7.8 5.3 6.6 5.6 0.3 0.6 0.6 0.6 0.6
0.3 0.2 0.4 0.4 0.2 2.3 2.0 2.0 2.0 1.7 0.6 0.7 0.8 0.5 0.7 2.1 2.1 2.1 2.0 1.5 0.1 0.5 0.6 0.7 0.6 1.5 2.0 2.0 2.0 2.0
38 34 30 26 22 18 14 10 6 3
1 4 7 11 15 19 23 27 31 35
Odds Ratio

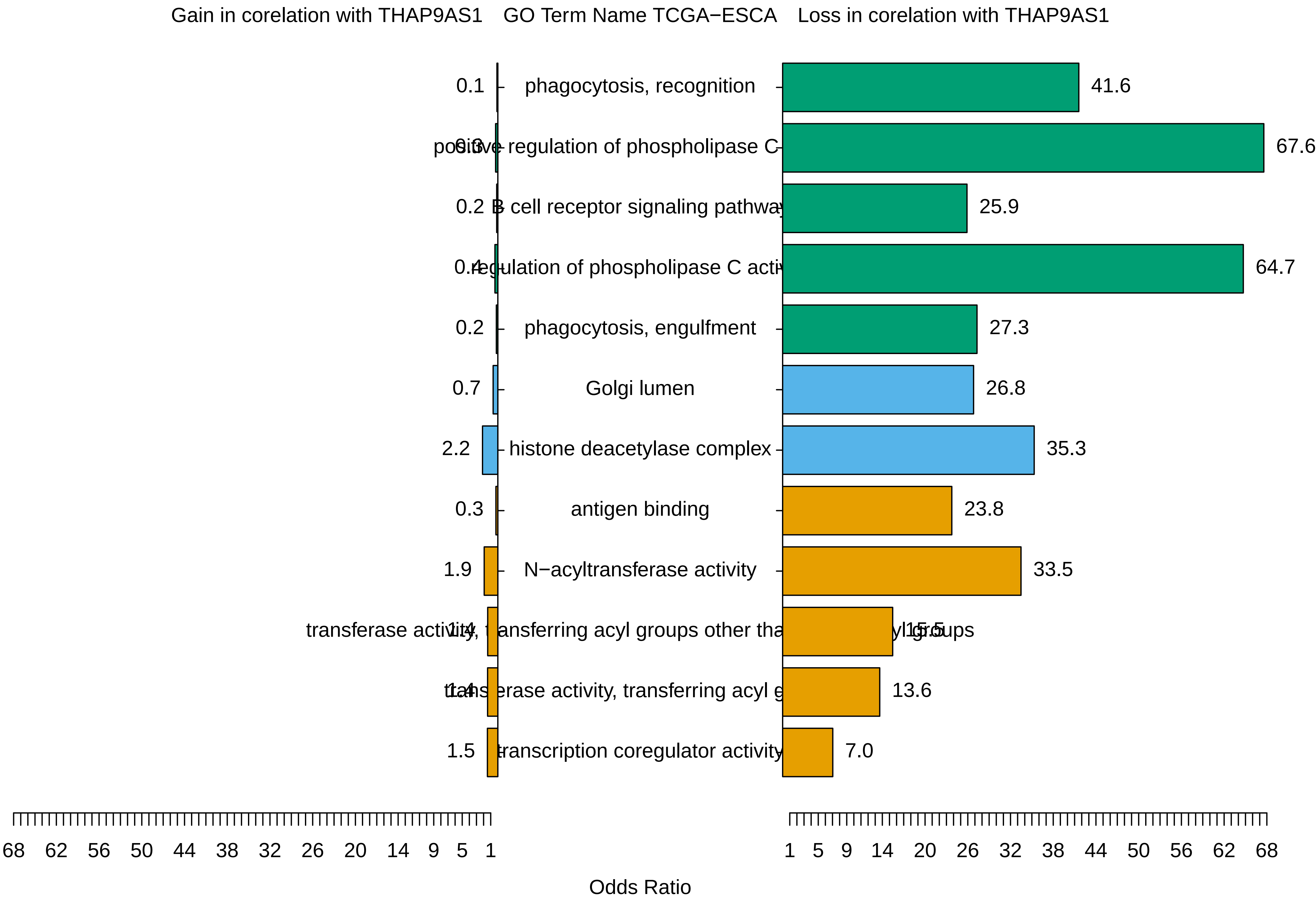

Gain in corelation with THAP9AS1
GO Term Name TCGA−ESCA
Loss in corelation with THAP9AS1
0.1
phagocytosis, recognition
41.6
positive regulation of phospholipase C
67.6
0.2
B cell receptor signaling pathwa
25.9
regulation of phospholipase C acti
64.7
0.2
phagocytosis, engulfment
27.3
0.7
Golgi lumen
26.8
2.2
histone deacetylase complex
35.3
0.3
antigen binding
23.8
1.9
N−acyltransferase activity
33.5
transferase activity, transferring acyl groups other tha
erase activity, transferring acyl g
1.4
13.6
1.5
transcription coregulator activity
7.0
68 62 56 50 44 38 32 26 20 14 9 5 1
1 5 9 14 20 26 32 38 44 50 56 62 68
Odds Ratio

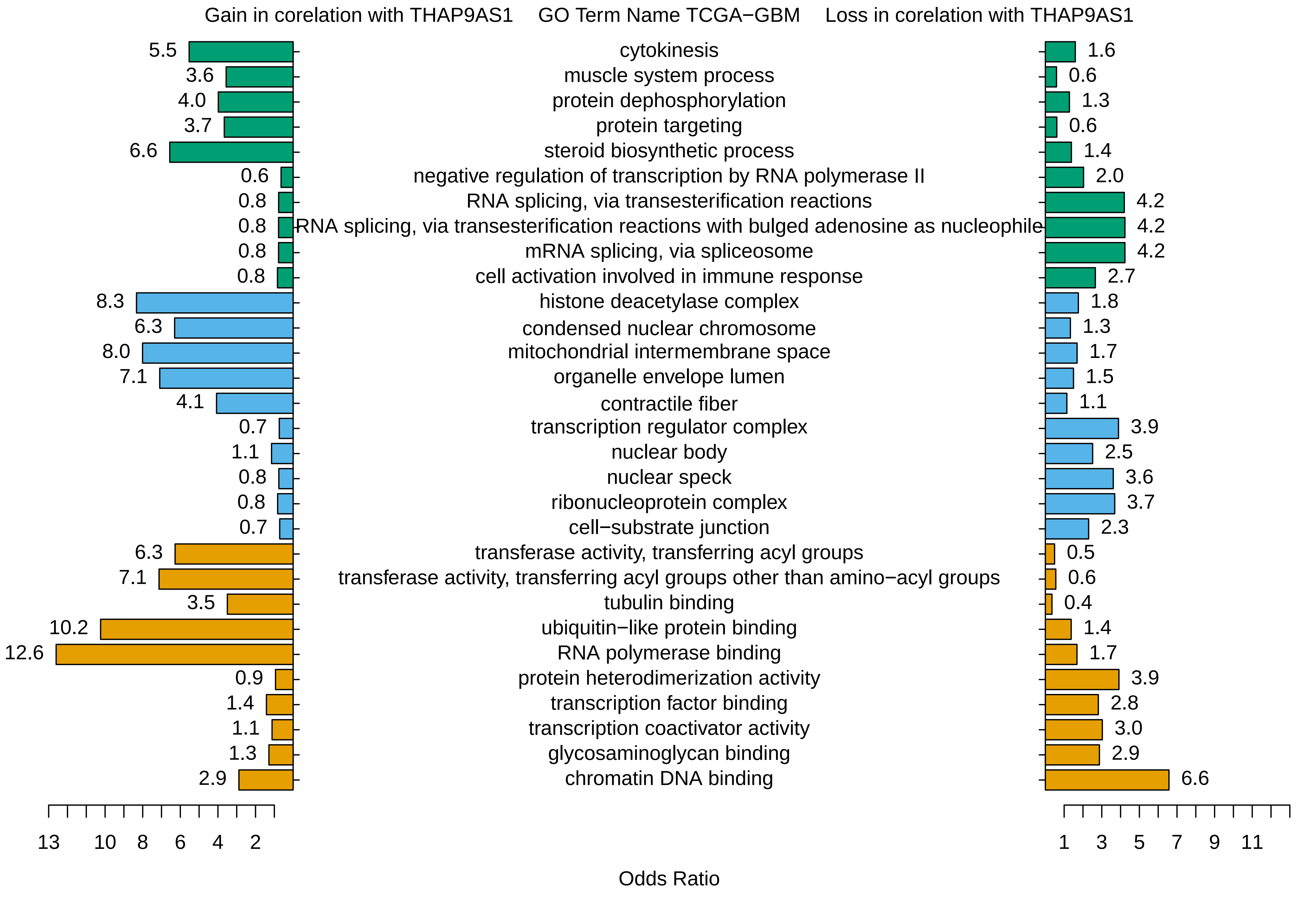
Gain in corelation with THAP9AS1
GO Term Name TCGA−GBM
Loss in corelation with THAP9AS1
5.5 cytokinesis 1.6
3.6 muscle system process 0.6
4.0 protein dephosphorylation 1.3
3.7 protein targeting 0.6
6.6 steroid biosynthetic process 1.4
0.6 negative regulation of transcription by RNA polymerase II 2.0
0.8 RNA splicing, via transesterification reactions 4.2
0.8 RNA splicing, via transesterification reactions with bulged adenosine as nucleophile 4.2
0.8 mRNA splicing, via spliceosome 4.2
0.8 cell activation involved in immune response 2.7
8.3 histone deacetylase complex 1.8
6.3 condensed nuclear chromosome 1.3
8.0 mitochondrial intermembrane space 1.7
7.1 organelle envelope lumen 1.5
4.1 contractile fiber 1.1
0.7 transcription regulator complex 3.9
1.1 nuclear body 2.5
0.8 nuclear speck 3.6
0.8 ribonucleoprotein complex 3.7
0.7 cell−substrate junction 2.3
6.3 transferase activity, transferring acyl groups 0.5
7.1 transferase activity, transferring acyl groups other than amino−acyl groups 0.6
3.5 tubulin binding 0.4
10.2 ubiquitin−like protein binding 1.4
12.6 RNA polymerase binding 1.7
0.9 protein heterodimerization activity 3.9
1.4 transcription factor binding 2.8
1.1 transcription coactivator activity 3.0
1.3 glycosaminoglycan binding 2.9
2.9 chromatin DNA binding 6.6
13 10 8 6 4 2
1 3 5 7 9 11
Odds Ratio

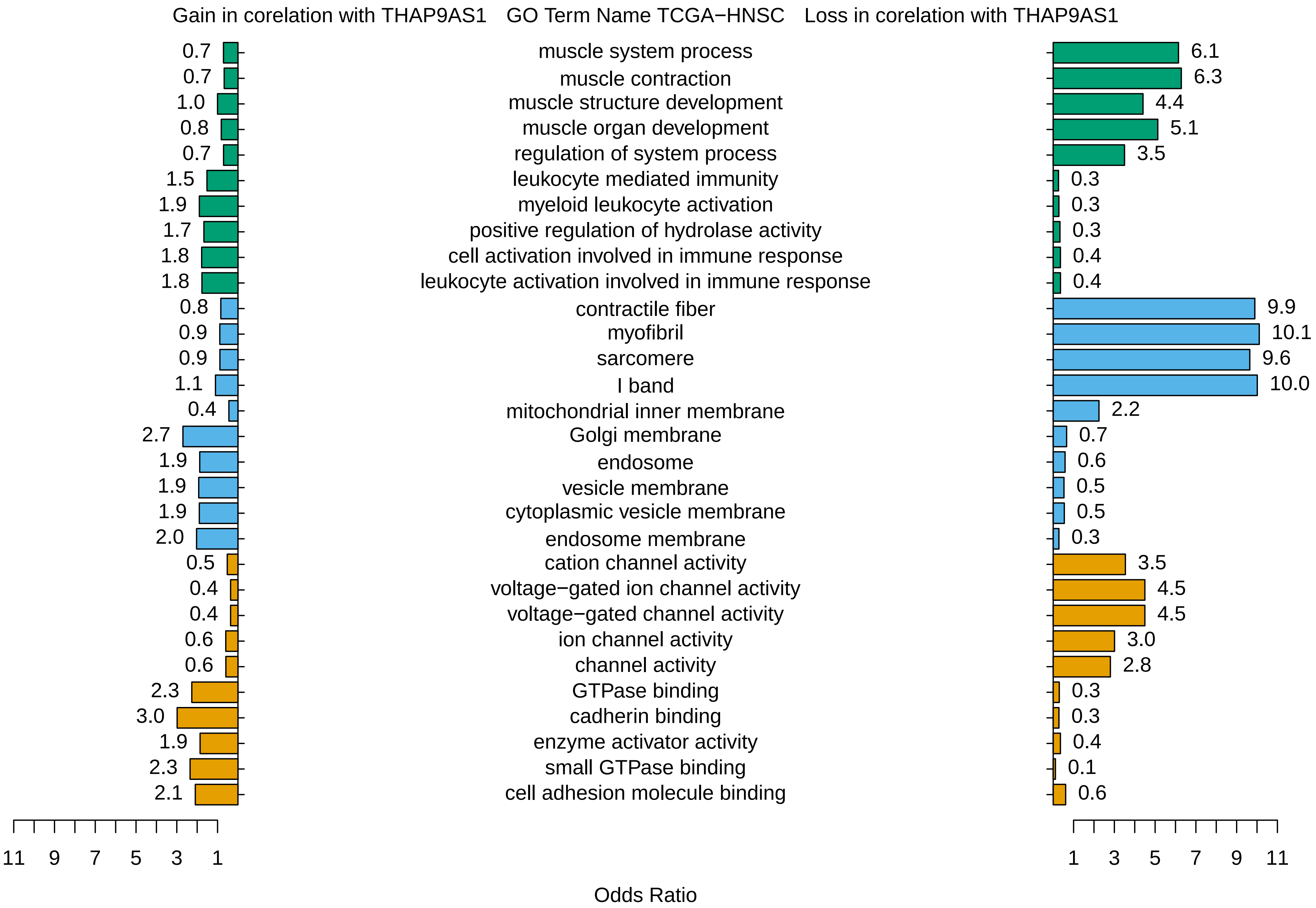
Gain in corelation with THAP9AS1
GO Term Name TCGA−HNSC
Loss in corelation with THAP9AS1
0.7 muscle system process 6.1
0.7 muscle contraction 6.3
1.0 muscle structure development 4.4
0.8 muscle organ development 5.1
0.7 regulation of system process 3.5
1.5 leukocyte mediated immunity 0.3
1.9 myeloid leukocyte activation 0.3
1.7 positive regulation of hydrolase activity 0.3
1.8 cell activation involved in immune response 0.4
1.8 leukocyte activation involved in immune response 0.4
0.8 contractile fiber 9.9
0.9 myofibril 10.1
0.9 sarcomere 9.6
1.1 I band 10.0
0.4 mitochondrial inner membrane 2.2
2.7 Golgi membrane 0.7
1.9 endosome 0.6
1.9 vesicle membrane 0.5
1.9 cytoplasmic vesicle membrane 0.5
2.0 endosome membrane 0.3
0.5 cation channel activity 3.5
0.4 voltage−gated ion channel activity 4.5
0.4 voltage−gated channel activity 4.5
0.6 ion channel activity 3.0
0.6 channel activity 2.8
2.3 GTPase binding 0.3
3.0 cadherin binding 0.3
1.9 enzyme activator activity 0.4
2.3 small GTPase binding 0.1
2.1 cell adhesion molecule binding 0.6
11 9 7 5 3 1
1 3 5 7 9 11
Odds Ratio

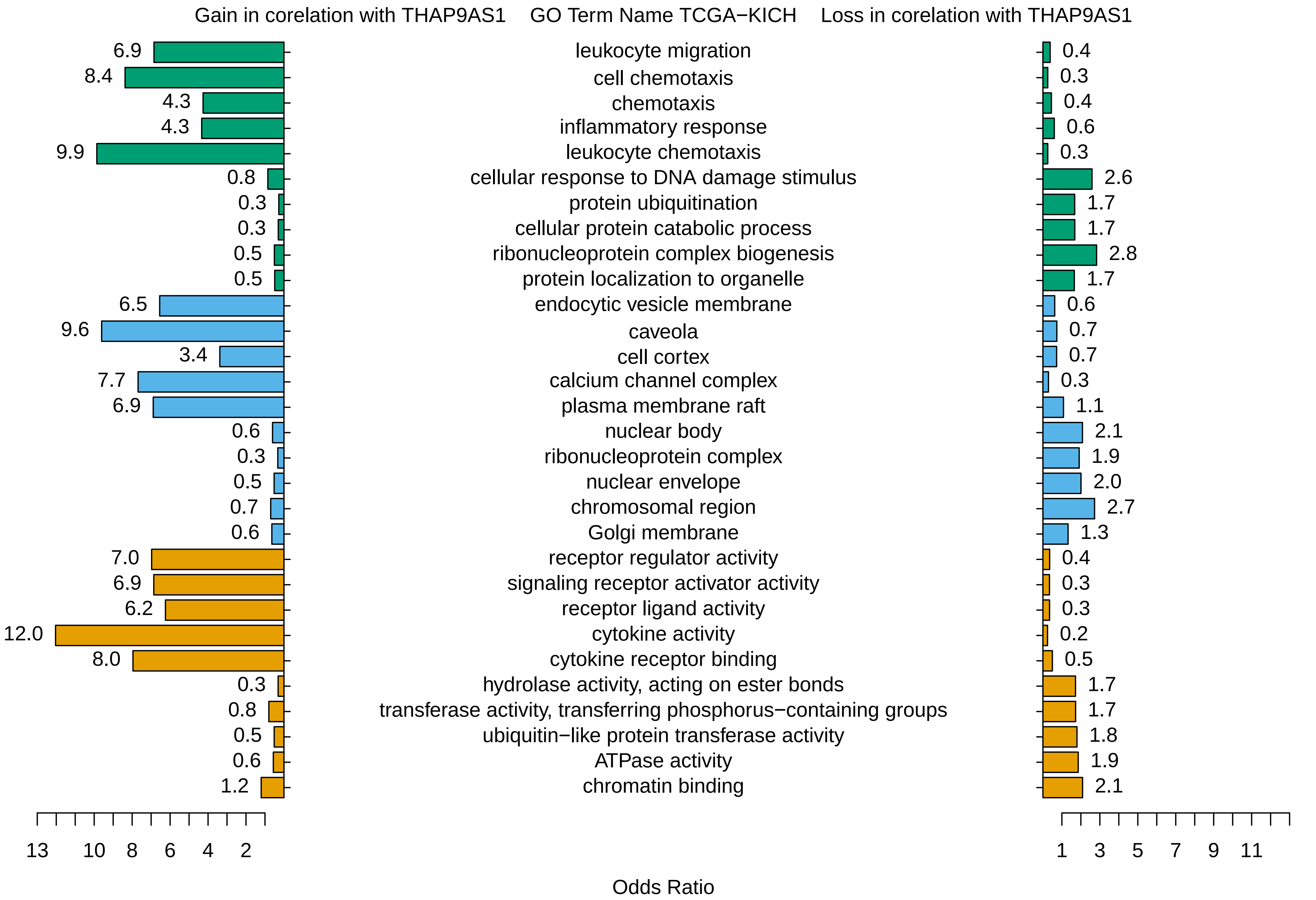

Gain in corelation with THAP9AS1
GO Term Name TCGA−KICH
Loss in corelation with THAP9AS1
6.9 leukocyte migration 0.4
8.4 cell chemotaxis 0.3
4.3 chemotaxis 0.4
4.3 inflammatory response 0.6
9.9 leukocyte chemotaxis 0.3
0.8 cellular response to DNA damage stimulus 2.6
0.3 protein ubiquitination 1.7
0.3 cellular protein catabolic process 1.7
0.5 ribonucleoprotein complex biogenesis 2.8
0.5 protein localization to organelle 1.7
6.5 endocytic vesicle membrane 0.6
9.6 caveola 0.7
3.4 cell cortex 0.7
7.7 calcium channel complex 0.3
6.9 plasma membrane raft 1.1
0.6 nuclear body 2.1
0.3 ribonucleoprotein complex 1.9
0.5 nuclear envelope 2.0
0.7 chromosomal region 2.7
0.6 Golgi membrane 1.3
7.0 receptor regulator activity 0.4
6.9 signaling receptor activator activity 0.3
6.2 receptor ligand activity 0.3
12.0 cytokine activity 0.2
8.0 cytokine receptor binding 0.5
0.3 hydrolase activity, acting on ester bonds 1.7
0.8 transferase activity, transferring phosphorus−containing groups 1.7
0.5 ubiquitin−like protein transferase activity 1.8
0.6 ATPase activity 1.9
1.2 chromatin binding 2.1
13 10 8 6 4 2
1 3 5 7 9 11
Odds Ratio

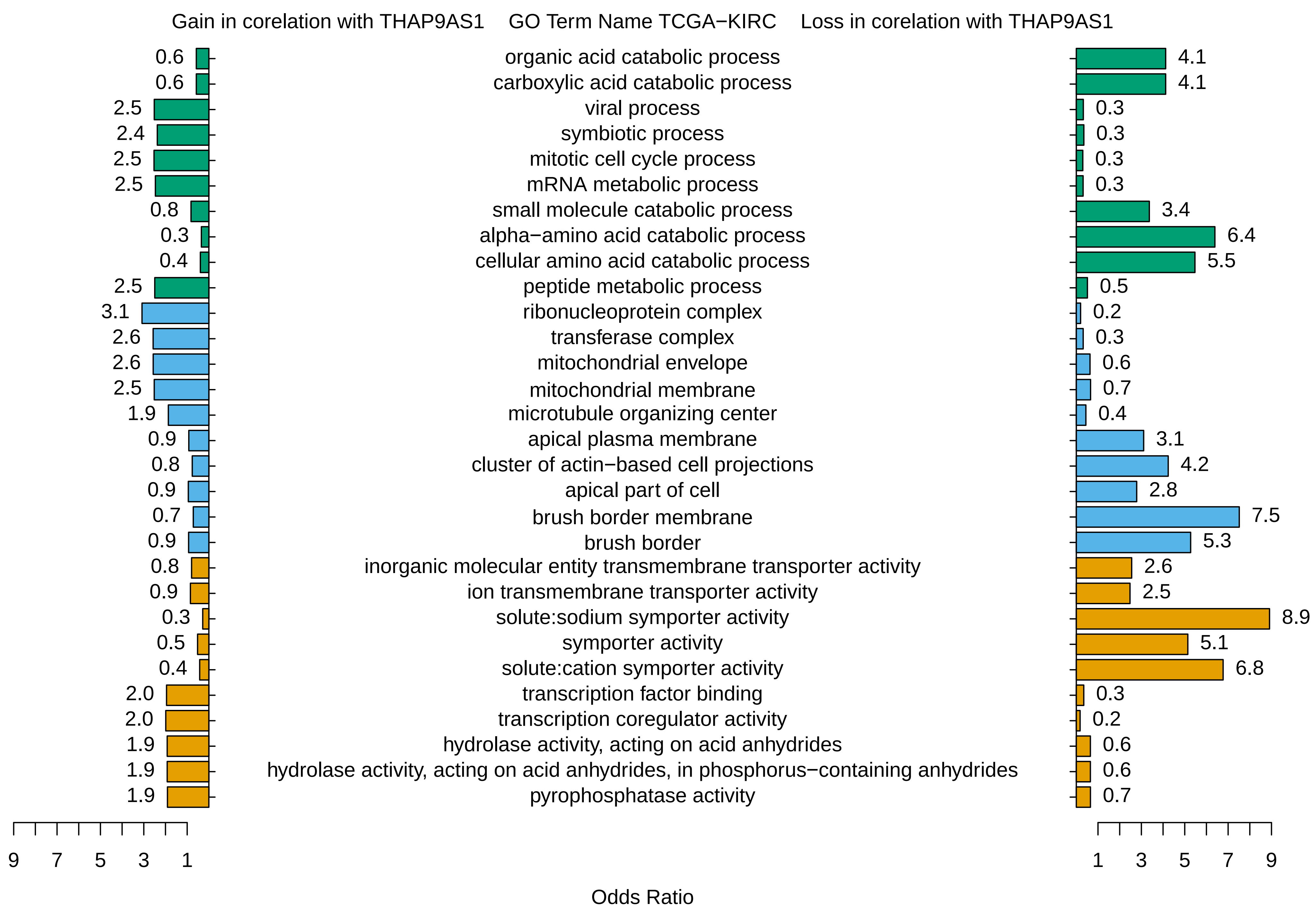

Gain in corelation with THAP9AS1
GO Term Name TCGA−KIRC
Loss in corelation with THAP9AS1
0.6 organic acid catabolic process 4.1
0.6 carboxylic acid catabolic process 4.1
2.5 viral process 0.3
2.4 symbiotic process 0.3
2.5 mitotic cell cycle process 0.3
2.5 mRNA metabolic process 0.3
0.8 small molecule catabolic process 3.4
0.3 alpha−amino acid catabolic process 6.4
0.4 cellular amino acid catabolic process 5.5
2.5 peptide metabolic process 0.5
3.1 ribonucleoprotein complex 0.2
2.6 transferase complex 0.3
2.6 mitochondrial envelope 0.6
2.5 mitochondrial membrane 0.7
1.9 microtubule organizing center 0.4
0.9 apical plasma membrane 3.1
0.8 cluster of actin−based cell projections 4.2
0.9 apical part of cell 2.8
0.7 brush border membrane 7.5
0.9 brush border 5.3
0.8 inorganic molecular entity transmembrane transporter activity 2.6
0.9 ion transmembrane transporter activity 2.5
0.3 solute:sodium symporter activity 8.9
0.5 symporter activity 5.1
0.4 solute:cation symporter activity 6.8
2.0 transcription factor binding 0.3
2.0 transcription coregulator activity 0.2
1.9 hydrolase activity, acting on acid anhydrides 0.6
1.9 hydrolase activity, acting on acid anhydrides, in phosphorus−containing anhydrides 0.6
1.9 pyrophosphatase activity 0.7
9 7 5 3 1
1 3 5 7 9
Odds Ratio

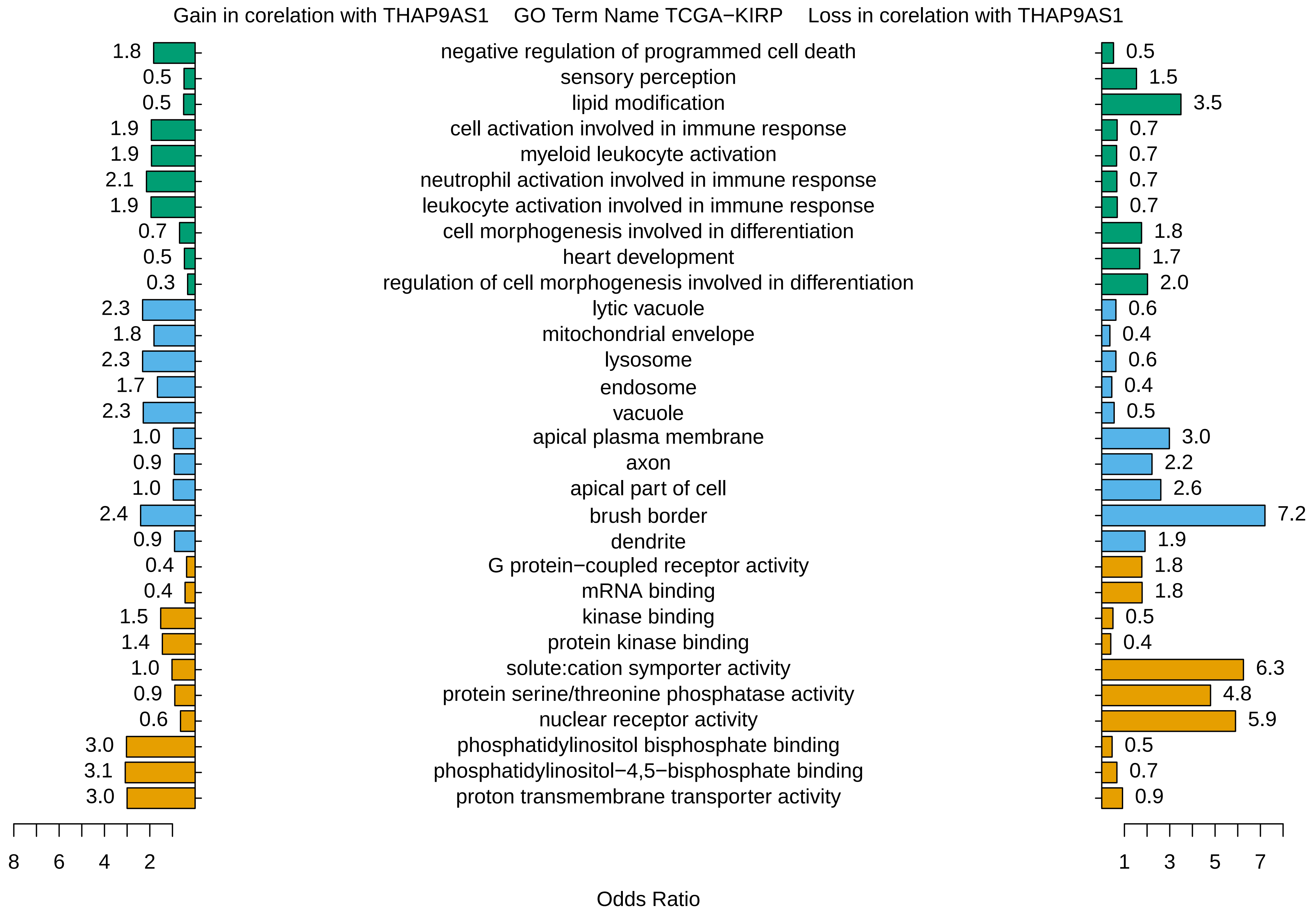
Gain in corelation with THAP9AS1
GO Term Name TCGA−KIRP
Loss in corelation with THAP9AS1
negative regulation of programmed cell death
sensory perception
lipid modification
cell activation involved in immune response
myeloid leukocyte activation
neutrophil activation involved in immune response
leukocyte activation involved in immune response
cell morphogenesis involved in differentiation
heart development
regulation of cell morphogenesis involved in differentiation
lytic vacuole
mitochondrial envelope
lysosome
endosome
vacuole
apical plasma membrane
axon
apical part of cell
brush border
dendrite
G protein−coupled receptor activity
mRNA binding
kinase binding
protein kinase binding
solute:cation symporter activity
protein serine/threonine phosphatase activity
nuclear receptor activity
phosphatidylinositol bisphosphate binding
phosphatidylinositol−4,5−bisphosphate binding
proton transmembrane transporter activity
1.8 0.5 0.5 1.9 1.9 2.1 1.9 0.7 0.5 0.3 2.3 1.8 2.3 1.7 2.3 1.0 0.9 1.0 2.4 0.9 0.4 0.4 1.5 1.4 1.0 0.9 0.6 3.0 3.1 3.0
0.5 1.5 3.5 0.7 0.7 0.7 0.7 1.8 1.7 2.0 0.6 0.4 0.6 0.4 0.5 3.0 2.2 2.6 7.2 1.9 1.8 1.8 0.5 0.4 6.3 4.8 5.9 0.5 0.7 0.9
8 6 4 2
1 3 5 7
Odds Ratio

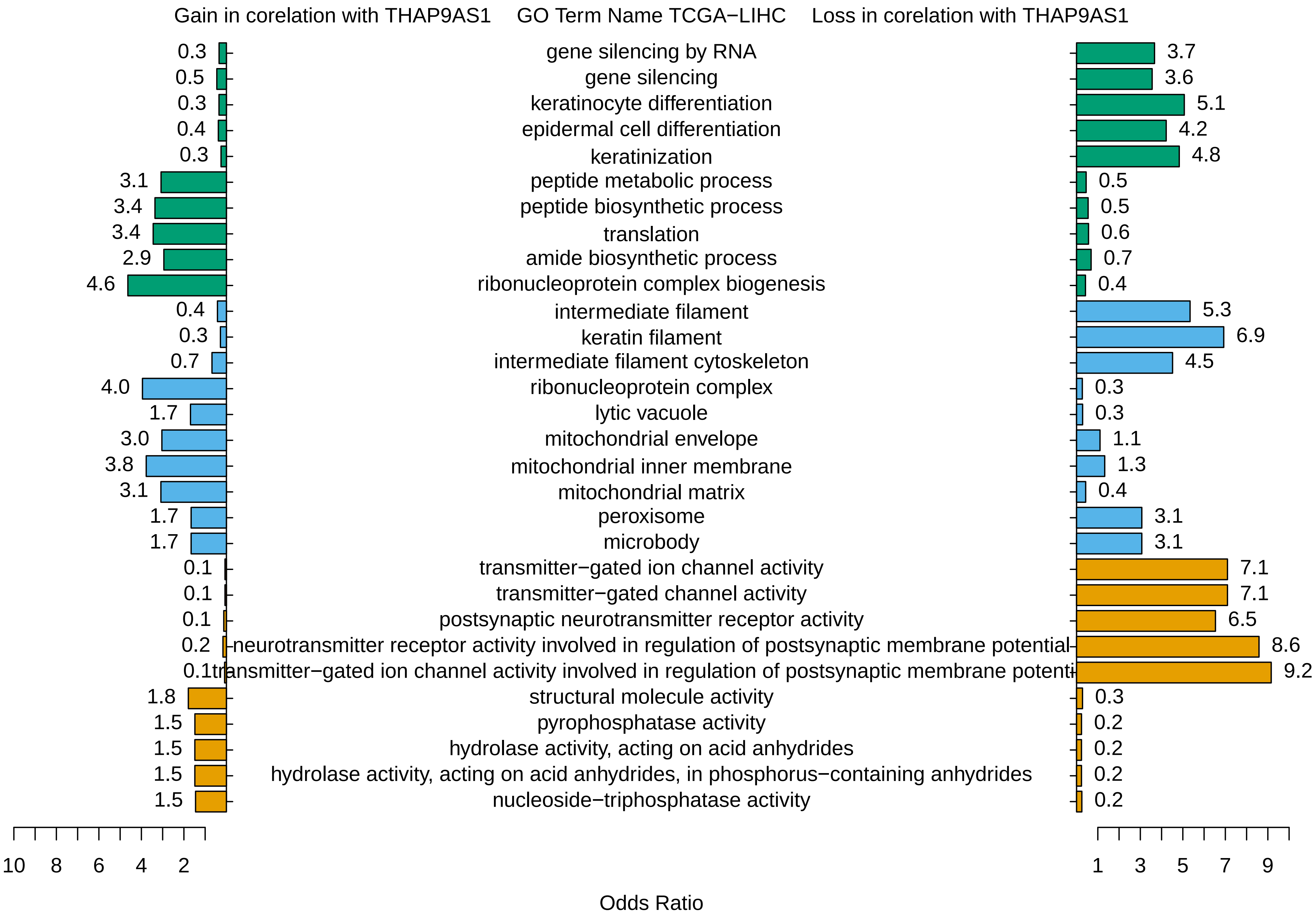

Gain in corelation with THAP9AS1
GO Term Name TCGA−LIHC
Loss in corelation with THAP9AS1
0.3 gene silencing by RNA 3.7
0.5 gene silencing 3.6
0.3 keratinocyte differentiation 5.1
0.4 epidermal cell differentiation 4.2
0.3 keratinization 4.8
3.1 peptide metabolic process 0.5
3.4 peptide biosynthetic process 0.5
3.4 translation 0.6
2.9 amide biosynthetic process 0.7
4.6 ribonucleoprotein complex biogenesis 0.4
0.4 intermediate filament 5.3
0.3 keratin filament 6.9
0.7 intermediate filament cytoskeleton 4.5
4.0 ribonucleoprotein complex 0.3
1.7 lytic vacuole 0.3
3.0 mitochondrial envelope 1.1
3.8 mitochondrial inner membrane 1.3
3.1 mitochondrial matrix 0.4
1.7 peroxisome 3.1
1.7 microbody 3.1
0.1 transmitter−gated ion channel activity 7.1
0.1 transmitter−gated channel activity 7.1
0.1 postsynaptic neurotransmitter receptor activity 6.5
0.2 neurotransmitter receptor activity involved in regulation of postsynaptic membrane potential 8.6
0.1 transmitter−gated ion channel activity involved in regulation of postsynaptic membrane potenti 9.2
1.8 structural molecule activity 0.3
1.5 pyrophosphatase activity 0.2
1.5 hydrolase activity, acting on acid anhydrides 0.2
1.5 hydrolase activity, acting on acid anhydrides, in phosphorus−containing anhydrides 0.2
1.5 nucleoside−triphosphatase activity 0.2
10 8 6 4 2
1 3 5 7 9
Odds Ratio

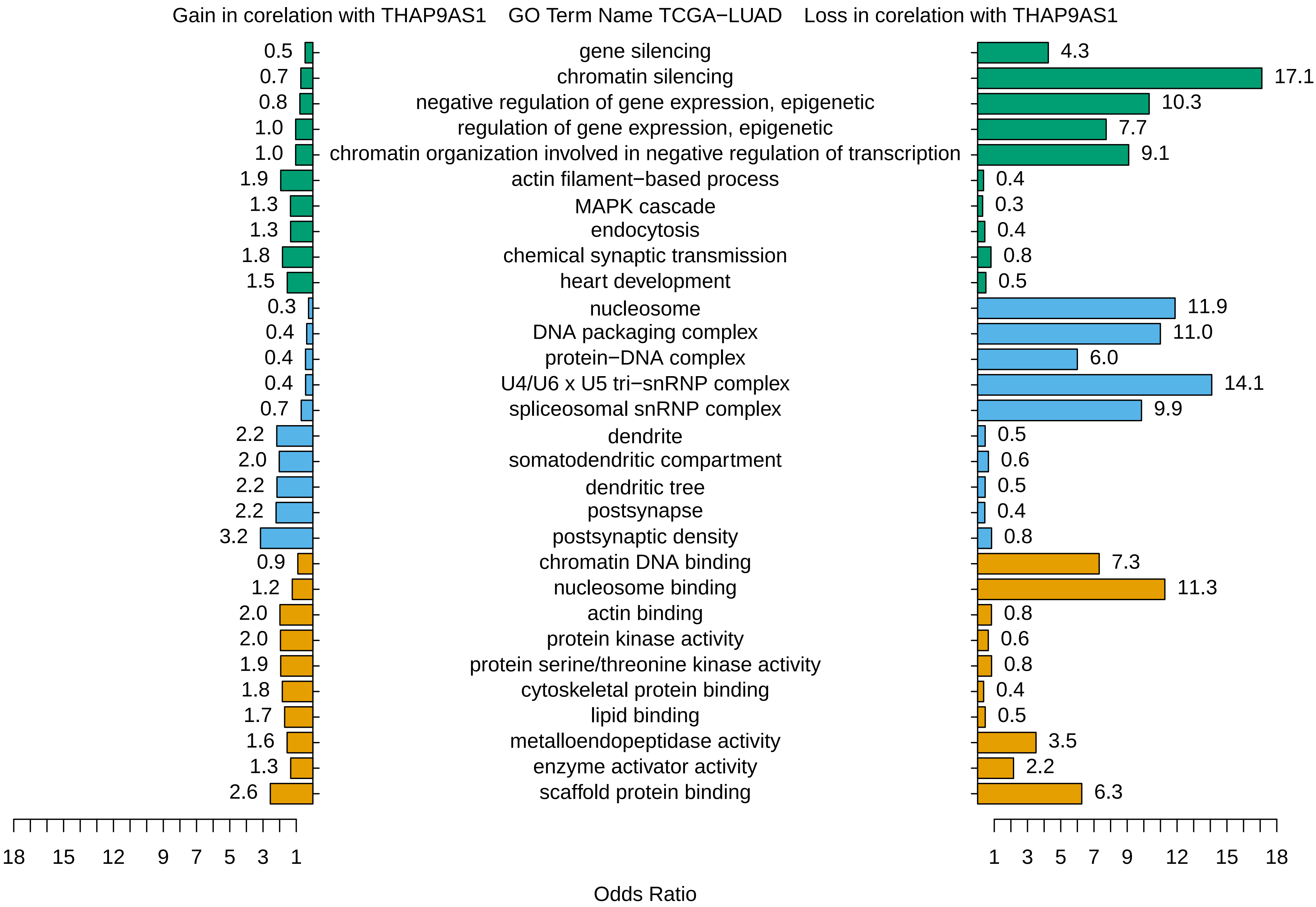

Gain in corelation with THAP9AS1
GO Term Name TCGA−LUAD
Loss in corelation with THAP9AS1
0.5 gene silencing 4.3
0.7 chromatin silencing 17.1
0.8 negative regulation of gene expression, epigenetic 10.3
1.0 regulation of gene expression, epigenetic 7.7
1.0 chromatin organization involved in negative regulation of transcription 9.1
1.9 actin filament−based process 0.4
1.3 MAPK cascade 0.3
1.3 endocytosis 0.4
1.8 chemical synaptic transmission 0.8
1.5 heart development 0.5
0.3 nucleosome 11.9
0.4 DNA packaging complex 11.0
0.4 protein−DNA complex 6.0
0.4 U4/U6 x U5 tri−snRNP complex 14.1
0.7 spliceosomal snRNP complex 9.9
2.2 dendrite 0.5
2.0 somatodendritic compartment 0.6
2.2 dendritic tree 0.5
2.2 postsynapse 0.4
3.2 postsynaptic density 0.8
0.9 chromatin DNA binding 7.3
1.2 nucleosome binding 11.3
2.0 actin binding 0.8
2.0 protein kinase activity 0.6
1.9 protein serine/threonine kinase activity 0.8
1.8 cytoskeletal protein binding 0.4
1.7 lipid binding 0.5
1.6 metalloendopeptidase activity 3.5
1.3 enzyme activator activity 2.2
2.6 scaffold protein binding 6.3
18 15 12 9 7 5 3 1
1 3 5 7 9 12 15 18
Odds Ratio

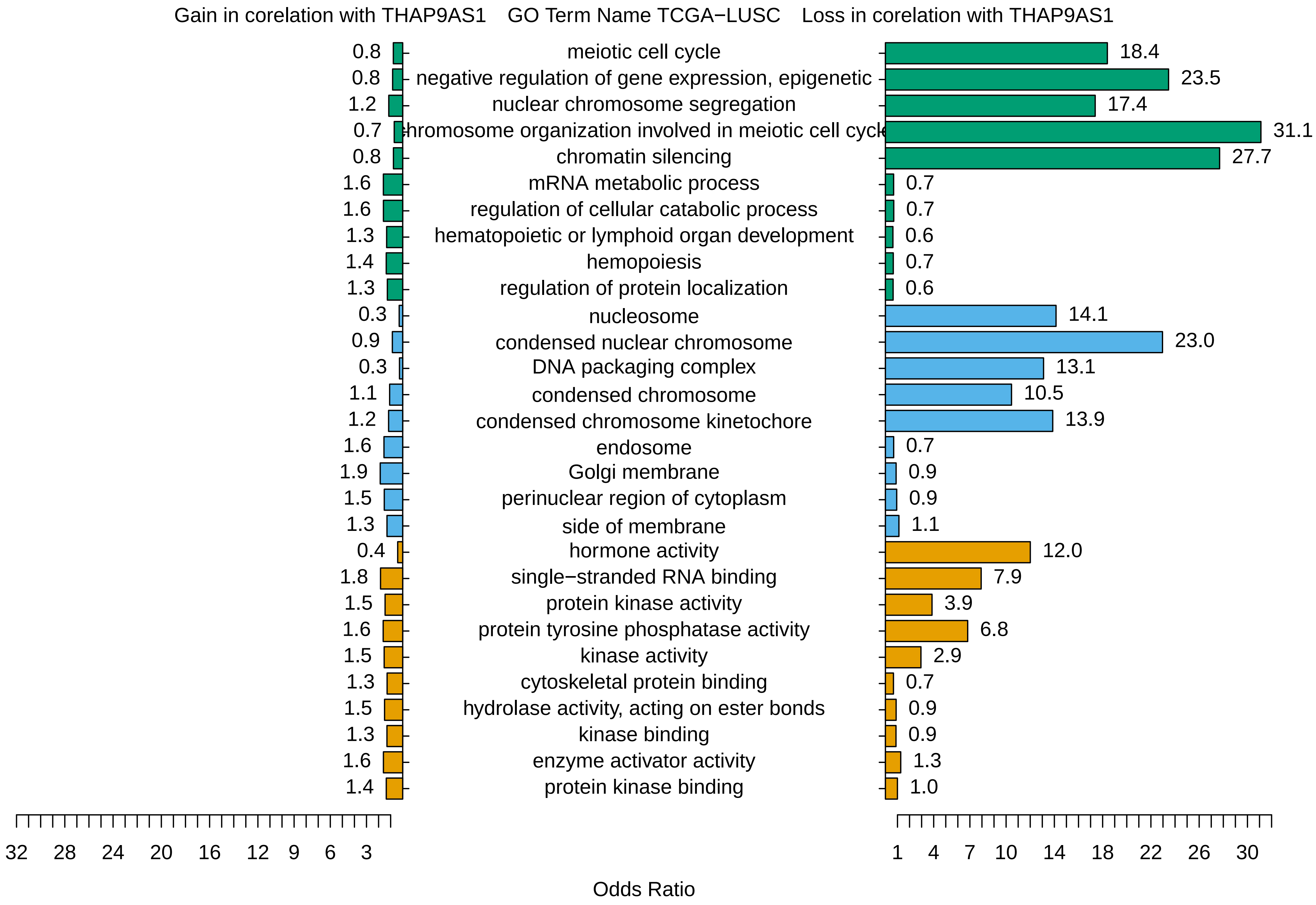

Gain in corelation with THAP9AS1
GO Term Name TCGA−LUSC
Loss in corelation with THAP9AS1
0.8 meiotic cell cycle 18.4
0.8 negative regulation of gene expression, epigenetic 23.5
1.2 nuclear chromosome segregation 17.4
0.7 hromosome organization involved in meiotic cell cycl 31.1
0.8 chromatin silencing 27.7
1.6 mRNA metabolic process 0.7
1.6 regulation of cellular catabolic process 0.7
1.3 hematopoietic or lymphoid organ development 0.6
1.4 hemopoiesis 0.7
1.3 regulation of protein localization 0.6
0.3 nucleosome 14.1
0.9 condensed nuclear chromosome 23.0
0.3 DNA packaging complex 13.1
1.1 condensed chromosome 10.5
1.2 condensed chromosome kinetochore 13.9
1.6 endosome 0.7
1.9 Golgi membrane 0.9
1.5 perinuclear region of cytoplasm 0.9
1.3 side of membrane 1.1
0.4 hormone activity 12.0
1.8 single−stranded RNA binding 7.9
1.5 protein kinase activity 3.9
1.6 protein tyrosine phosphatase activity 6.8
1.5 kinase activity 2.9
1.3 cytoskeletal protein binding 0.7
1.5 hydrolase activity, acting on ester bonds 0.9
1.3 kinase binding 0.9
1.6 enzyme activator activity 1.3
1.4 protein kinase binding 1.0
32 28 24 20 16 12 9 6 3
1 4 7 10 14 18 22 26 30
Odds Ratio

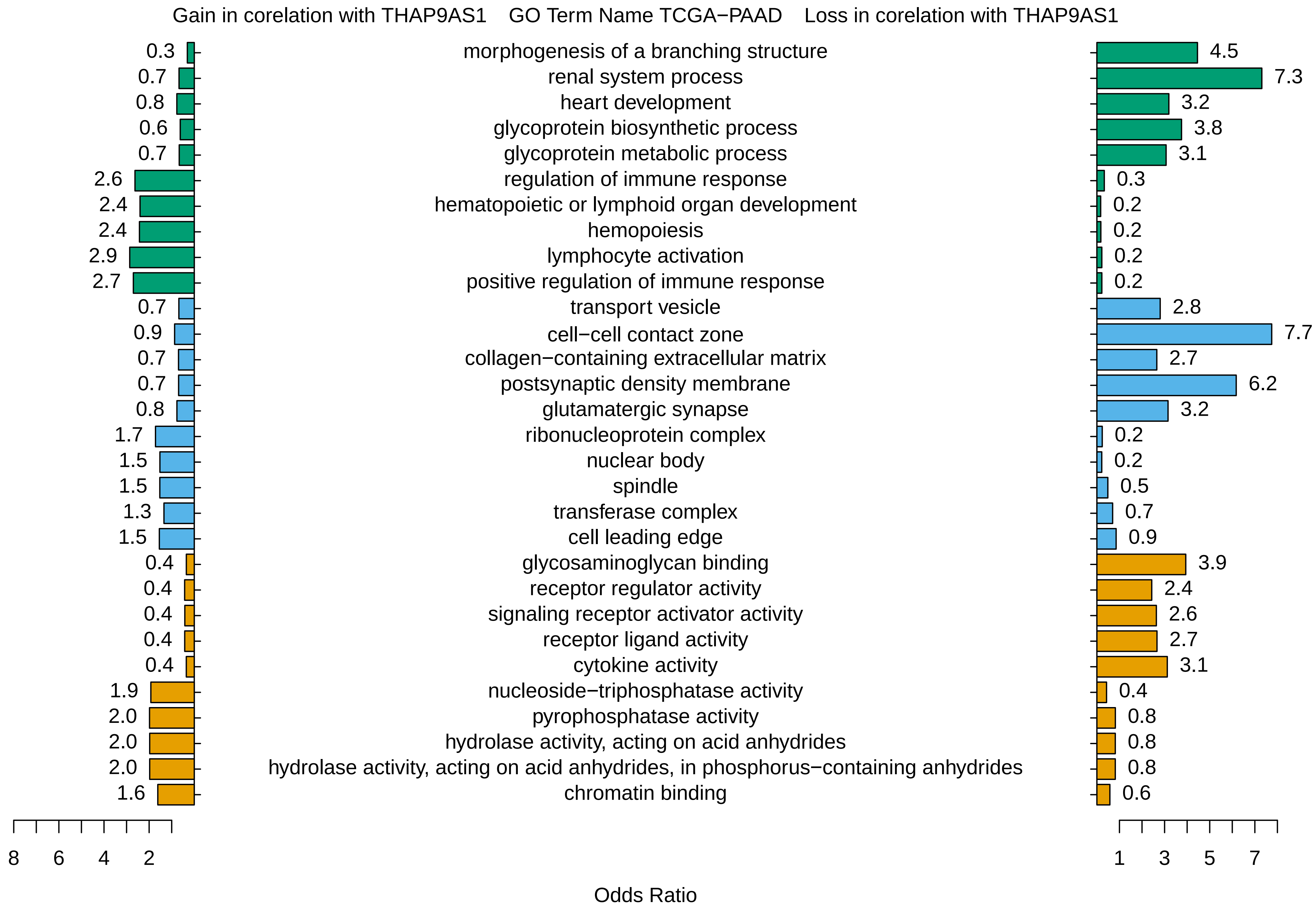

Gain in corelation with THAP9AS1
GO Term Name TCGA−PAAD
Loss in corelation with THAP9AS1
morphogenesis of a branching structure
renal system process
heart development
glycoprotein biosynthetic process
glycoprotein metabolic process
regulation of immune response
hematopoietic or lymphoid organ development
hemopoiesis
lymphocyte activation
positive regulation of immune response
transport vesicle
cell−cell contact zone
collagen−containing extracellular matrix
postsynaptic density membrane
glutamatergic synapse
ribonucleoprotein complex
nuclear body
spindle
transferase complex
cell leading edge
glycosaminoglycan binding
receptor regulator activity
signaling receptor activator activity
receptor ligand activity
cytokine activity
nucleoside−triphosphatase activity
pyrophosphatase activity
hydrolase activity, acting on acid anhydrides
hydrolase activity, acting on acid anhydrides, in phosphorus−containing anhydrides
chromatin binding
0.3 4.5
0.7 7.3
0.8 3.2
0.6 3.8
0.7 3.1
2.6 0.3
2.4 0.2
2.4 0.2
2.9 0.2
2.7 0.2
0.7 2.8
0.9 7.7
0.7 2.7
0.7 6.2
0.8 3.2
1.7 0.2
1.5 0.2
1.5 0.5
1.3 0.7
1.5 0.9
0.4 3.9
0.4 2.4
0.4 2.6
0.4 2.7
0.4 3.1
1.9 0.4
2.0 0.8
2.0 0.8
2.0 0.8
1.6 0.6
8 6 4 2
1 3 5 7
Odds Ratio

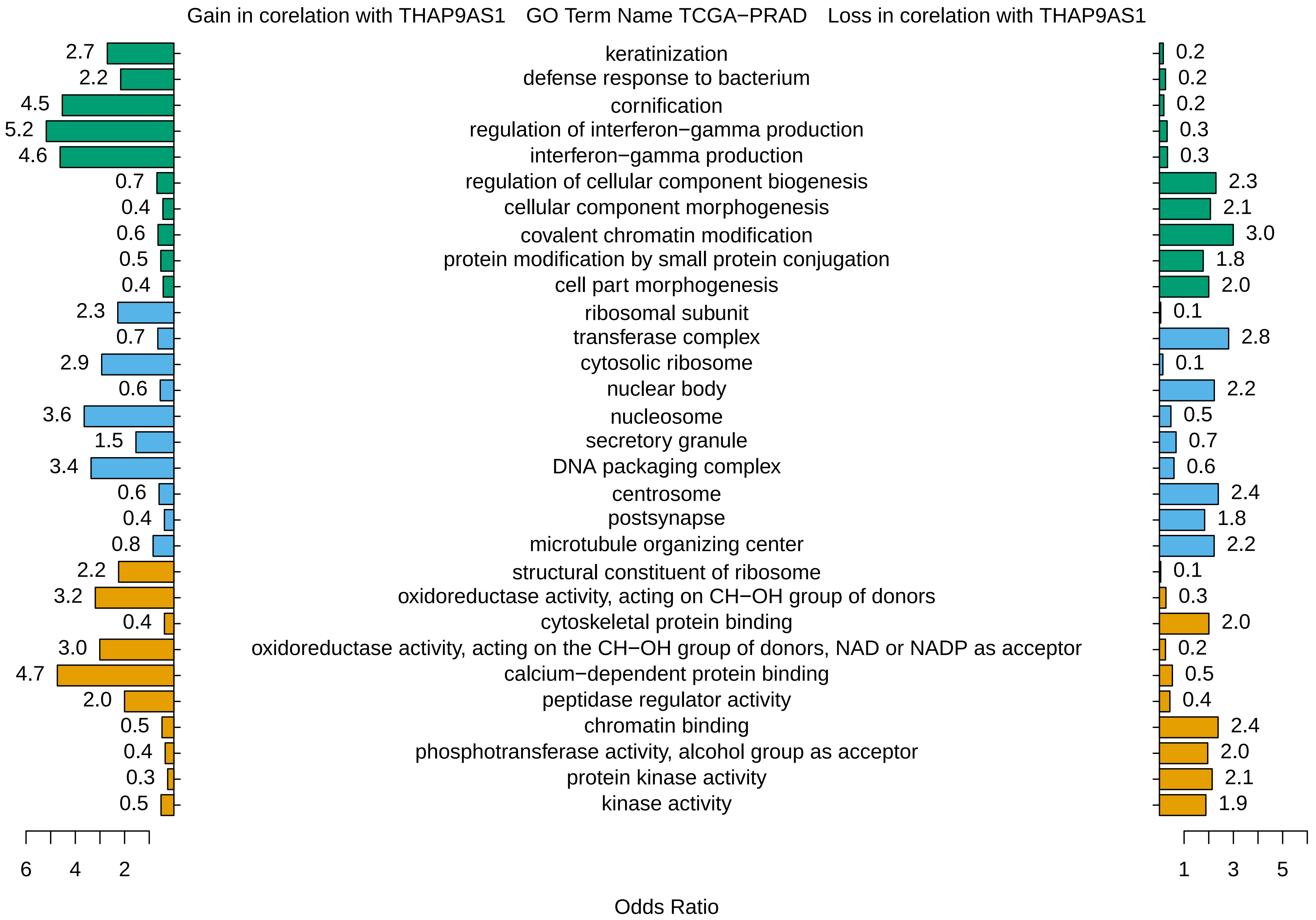

Gain in corelation with THAP9AS1
GO Term Name TCGA−PRAD
Loss in corelation with THAP9AS1
keratinization 2.7 0.2
defense response to bacterium 2.2 0.2
cornification 4.5 0.2
regulation of interferon−gamma production 5.2 0.3
interferon−gamma production 4.6 0.3
regulation of cellular component biogenesis 0.7 2.3
cellular component morphogenesis 0.4 2.1
covalent chromatin modification 0.6 3.0
protein modification by small protein conjugation 0.5 1.8
cell part morphogenesis 0.4 2.0
ribosomal subunit 2.3 0.1
transferase complex 0.7 2.8
cytosolic ribosome 2.9 0.1
nuclear body 0.6 2.2
nucleosome 3.6 0.5
secretory granule 1.5 0.7
DNA packaging complex 3.4 0.6
centrosome 0.6 2.4
postsynapse 0.4 1.8
microtubule organizing center 0.8 2.2
structural constituent of ribosome 2.2 0.1
oxidoreductase activity, acting on CH−OH group of donors 3.2 0.3
cytoskeletal protein binding 0.4 2.0
oxidoreductase activity, acting on the CH−OH group of donors, NAD or NADP as acceptor 3.0 0.2
calcium−dependent protein binding 4.7 0.5
peptidase regulator activity 2.0 0.4
chromatin binding 0.5 2.4
phosphotransferase activity, alcohol group as acceptor 0.4 2.0
protein kinase activity 0.3 2.1
kinase activity 0.5 1.9
6 4 2
1 3 5
Odds Ratio

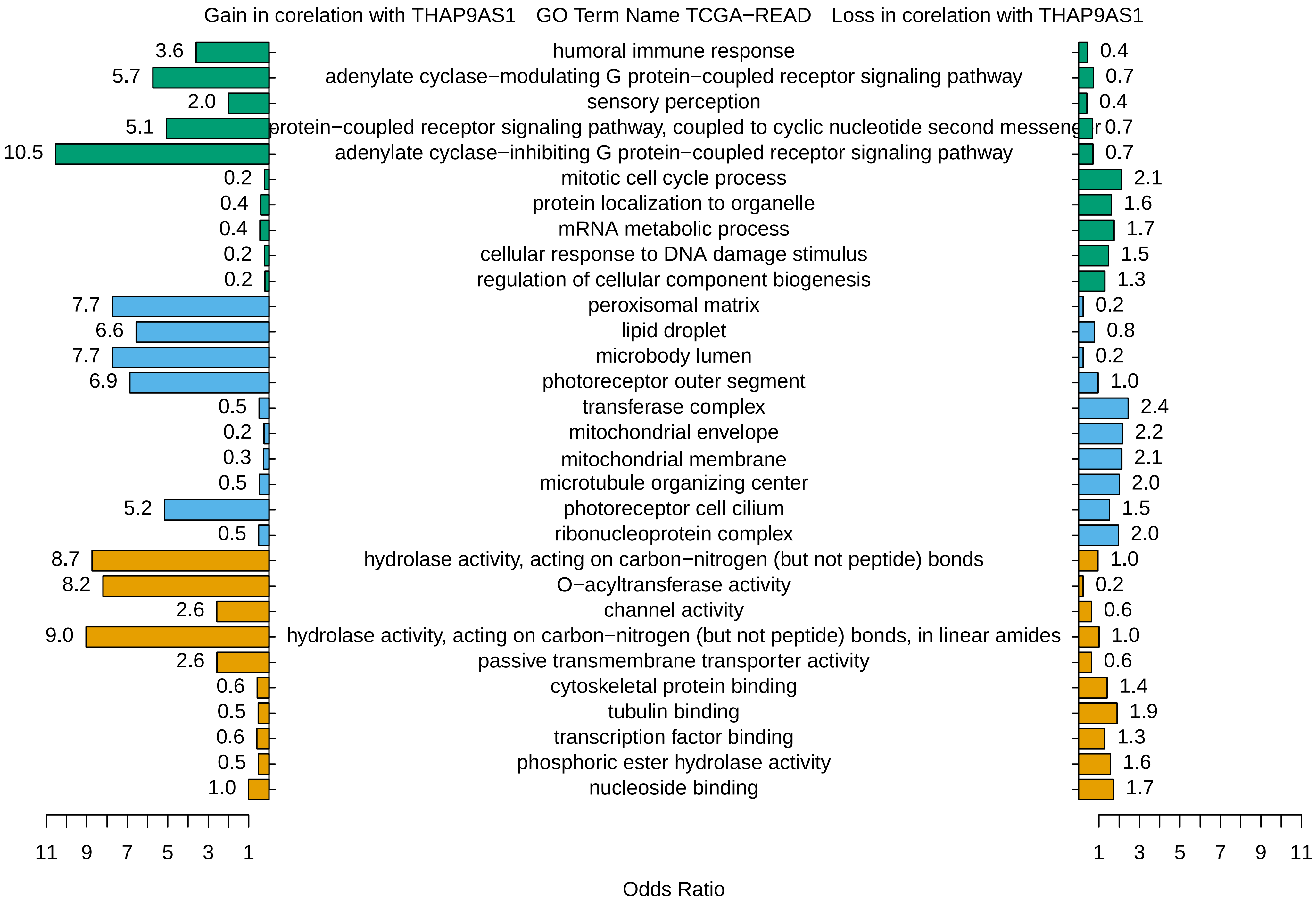
Gain in corelation with THAP9AS1
GO Term Name TCGA−READ
Loss in corelation with THAP9AS1
3.6 humoral immune response 0.4
5.7 adenylate cyclase−modulating G protein−coupled receptor signaling pathway 0.7
2.0 sensory perception 0.4
5.1 protein−coupled receptor signaling pathway, coupled to cyclic nucleotide second messeng r 0.7
10.5 adenylate cyclase−inhibiting G protein−coupled receptor signaling pathway 0.7
0.2 mitotic cell cycle process 2.1
0.4 protein localization to organelle 1.6
0.4 mRNA metabolic process 1.7
0.2 cellular response to DNA damage stimulus 1.5
0.2 regulation of cellular component biogenesis 1.3
7.7 peroxisomal matrix 0.2
6.6 lipid droplet 0.8
7.7 microbody lumen 0.2
6.9 photoreceptor outer segment 1.0
0.5 transferase complex 2.4
0.2 mitochondrial envelope 2.2
0.3 mitochondrial membrane 2.1
0.5 microtubule organizing center 2.0
5.2 photoreceptor cell cilium 1.5
0.5 ribonucleoprotein complex 2.0
8.7 hydrolase activity, acting on carbon−nitrogen (but not peptide) bonds 1.0
8.2 O−acyltransferase activity 0.2
2.6 channel activity 0.6
9.0 hydrolase activity, acting on carbon−nitrogen (but not peptide) bonds, in linear amides 1.0
2.6 passive transmembrane transporter activity 0.6
0.6 cytoskeletal protein binding 1.4
0.5 tubulin binding 1.9
0.6 transcription factor binding 1.3
0.5 phosphoric ester hydrolase activity 1.6
1.0 nucleoside binding 1.7
11 9 7 5 3 1
1 3 5 7 9 11
Odds Ratio

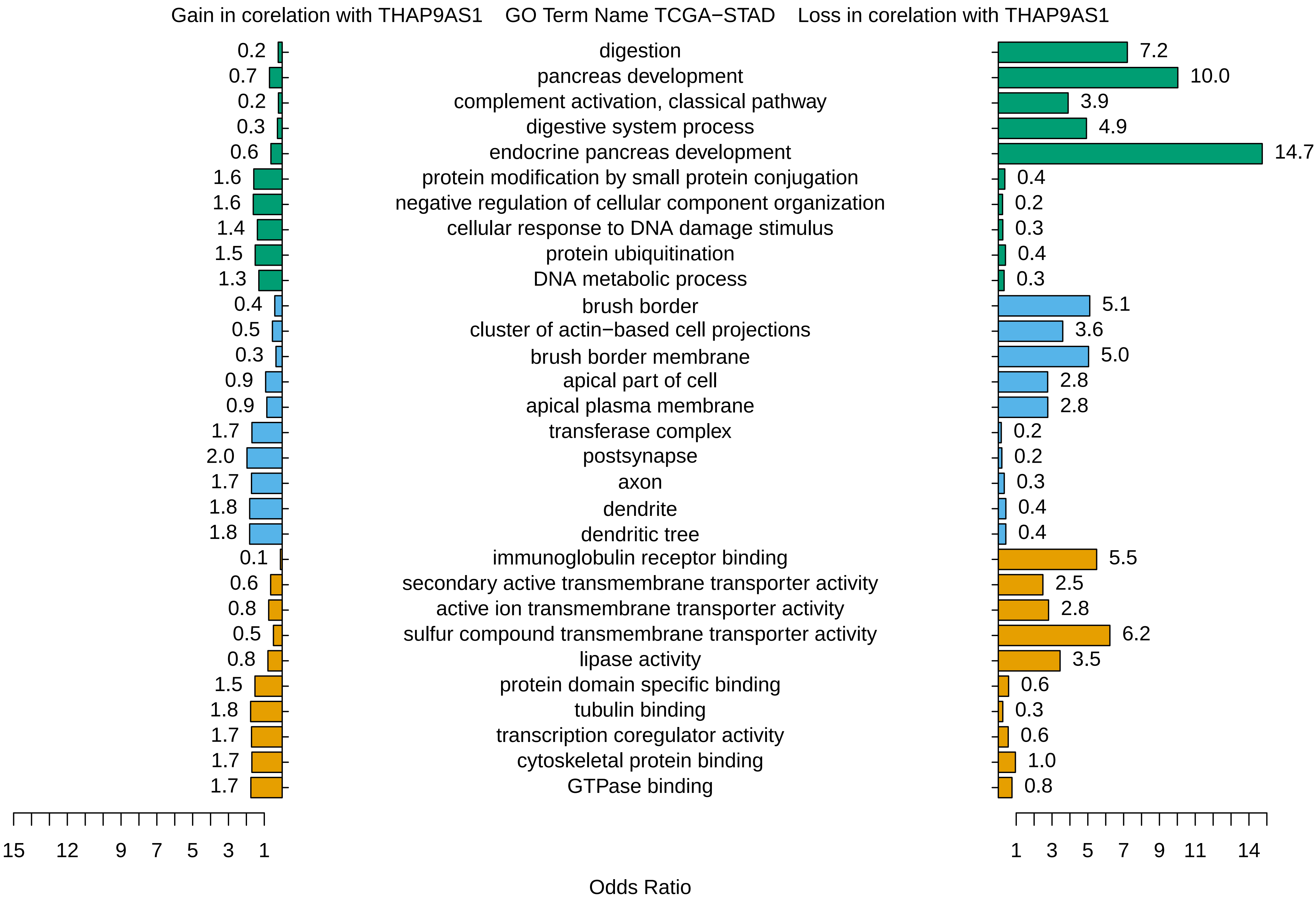

Gain in corelation with THAP9AS1
GO Term Name TCGA−STAD
Loss in corelation with THAP9AS1
0.2 digestion 7.2
0.7 pancreas development 10.0
0.2 complement activation, classical pathway 3.9
0.3 digestive system process 4.9
0.6 endocrine pancreas development 14.7
1.6 protein modification by small protein conjugation 0.4
1.6 negative regulation of cellular component organization 0.2
1.4 cellular response to DNA damage stimulus 0.3
1.5 protein ubiquitination 0.4
1.3 DNA metabolic process 0.3
0.4 brush border 5.1
0.5 cluster of actin−based cell projections 3.6
0.3 brush border membrane 5.0
0.9 apical part of cell 2.8
0.9 apical plasma membrane 2.8
1.7 transferase complex 0.2
2.0 postsynapse 0.2
1.7 axon 0.3
1.8 dendrite 0.4
1.8 dendritic tree 0.4
0.1 immunoglobulin receptor binding 5.5
0.6 secondary active transmembrane transporter activity 2.5
0.8 active ion transmembrane transporter activity 2.8
0.5 sulfur compound transmembrane transporter activity 6.2
0.8 lipase activity 3.5
1.5 protein domain specific binding 0.6
1.8 tubulin binding 0.3
1.7 transcription coregulator activity 0.6
1.7 cytoskeletal protein binding 1.0
1.7 GTPase binding 0.8
15 12 9 7 5 3 1
1 3 5 7 9 11 14
Odds Ratio

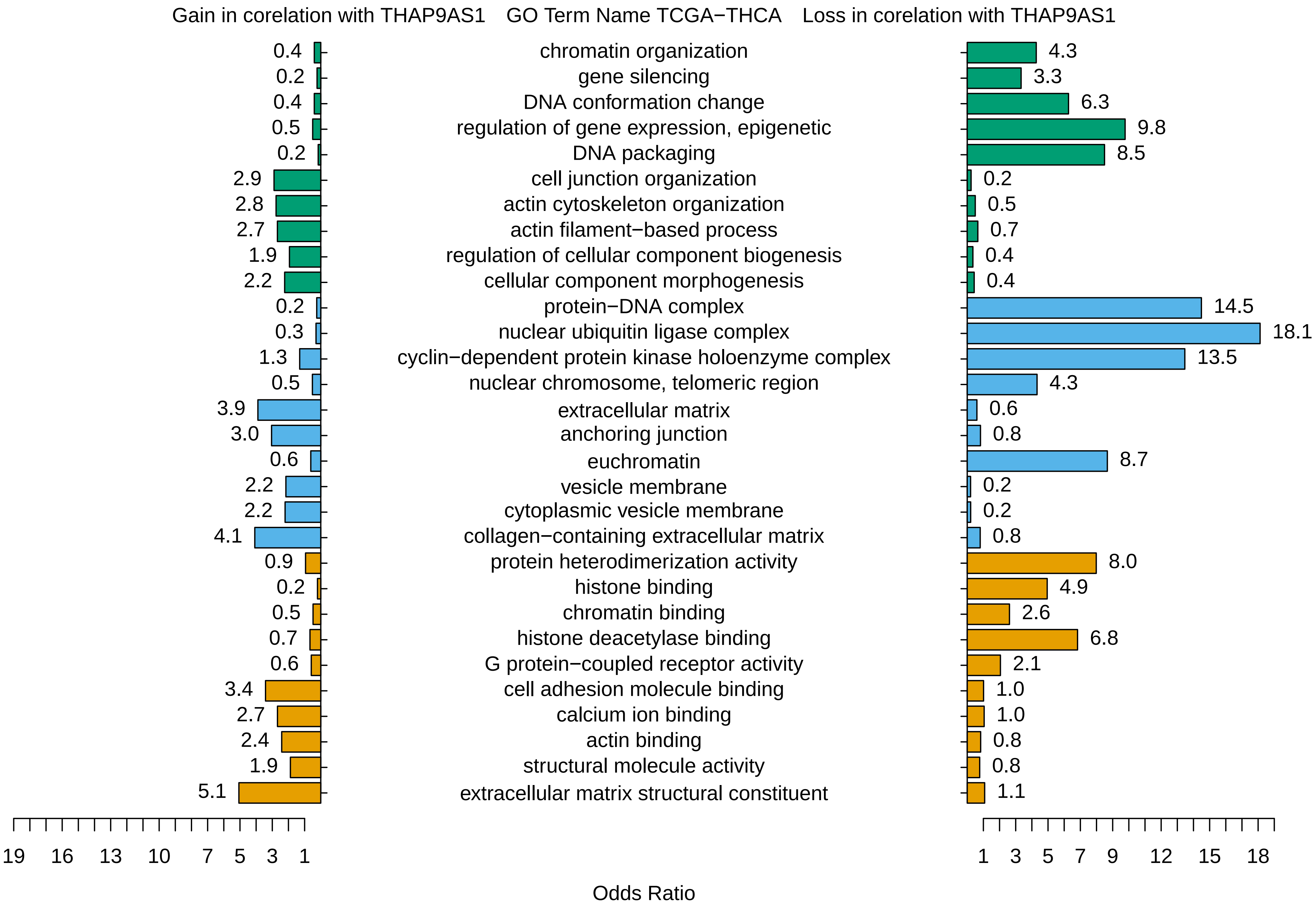

Gain in corelation with THAP9AS1
GO Term Name TCGA−THCA
Loss in corelation with THAP9AS1
0.4 chromatin organization 4.3
0.2 gene silencing 3.3
0.4 DNA conformation change 6.3
0.5 regulation of gene expression, epigenetic 9.8
0.2 DNA packaging 8.5
2.9 cell junction organization 0.2
2.8 actin cytoskeleton organization 0.5
2.7 actin filament−based process 0.7
1.9 regulation of cellular component biogenesis 0.4
2.2 cellular component morphogenesis 0.4
0.2 protein−DNA complex 14.5
0.3 nuclear ubiquitin ligase complex 18.1
1.3 cyclin−dependent protein kinase holoenzyme complex 13.5
0.5 nuclear chromosome, telomeric region 4.3
3.9 extracellular matrix 0.6
3.0 anchoring junction 0.8
0.6 euchromatin 8.7
2.2 vesicle membrane 0.2
2.2 cytoplasmic vesicle membrane 0.2
4.1 collagen−containing extracellular matrix 0.8
0.9 protein heterodimerization activity 8.0
0.2 histone binding 4.9
0.5 chromatin binding 2.6
0.7 histone deacetylase binding 6.8
0.6 G protein−coupled receptor activity 2.1
3.4 cell adhesion molecule binding 1.0
2.7 calcium ion binding 1.0
2.4 actin binding 0.8
1.9 structural molecule activity 0.8
5.1 extracellular matrix structural constituent 1.1
19 16 13 10 7 5 3 1
1 3 5 7 9 12 15 18
Odds Ratio

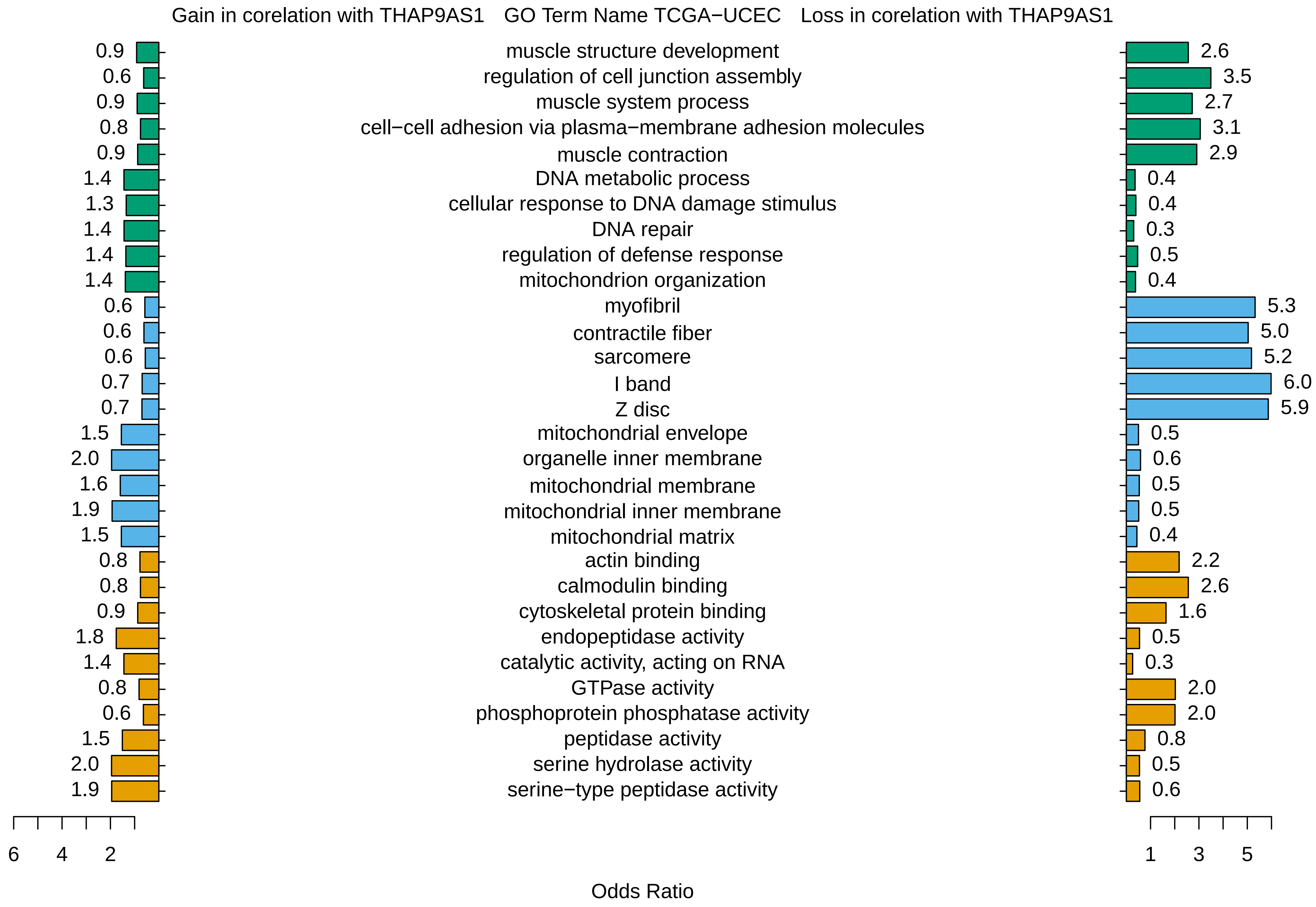

Gain in corelation with THAP9AS1
GO Term Name TCGA−UCEC
Loss in corelation with THAP9AS1
0.9 muscle structure development 2.6
0.6 regulation of cell junction assembly 3.5
0.9 muscle system process 2.7
0.8 cell−cell adhesion via plasma−membrane adhesion molecules 3.1
0.9 muscle contraction 2.9
1.4 DNA metabolic process 0.4
1.3 cellular response to DNA damage stimulus 0.4
1.4 DNA repair 0.3
1.4 regulation of defense response 0.5
1.4 mitochondrion organization 0.4
0.6 myofibril 5.3
0.6 contractile fiber 5.0
0.6 sarcomere 5.2
0.7 I band 6.0
0.7 Z disc 5.9
1.5 mitochondrial envelope 0.5
2.0 organelle inner membrane 0.6
1.6 mitochondrial membrane 0.5
1.9 mitochondrial inner membrane 0.5
1.5 mitochondrial matrix 0.4
0.8 actin binding 2.2
0.8 calmodulin binding 2.6
0.9 cytoskeletal protein binding 1.6
1.8 endopeptidase activity 0.5
1.4 catalytic activity, acting on RNA 0.3
0.8 GTPase activity 2.0
0.6 phosphoprotein phosphatase activity 2.0
1.5 peptidase activity 0.8
2.0 serine hydrolase activity 0.5
1.9 serine−type peptidase activity 0.6
6
4
2
1
3
5
Odds Ratio
