# Supplementary figures and images for: Pan-Cancer Analysis Reveals the Prognostic Potential of the THAP9/THAP9-AS1 Sense–Antisense Gene Pair in Human Cancers
Source: Noncoding RNA. 2022 Jul 8;8(4):51. doi: 10.3390/ncrna8040051 (PMC9326536; doi:10.3390/ncrna8040051)

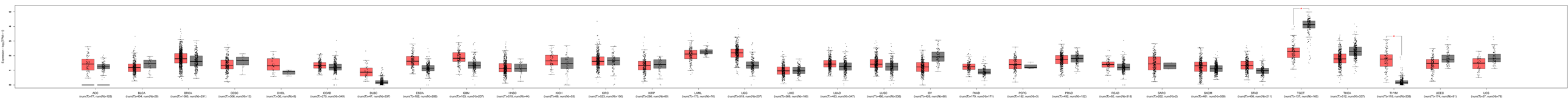

Supplement: Supplementary file 1 [file ncrna-08-00051-s001.zip › Supplementary Figure S2.pdf]

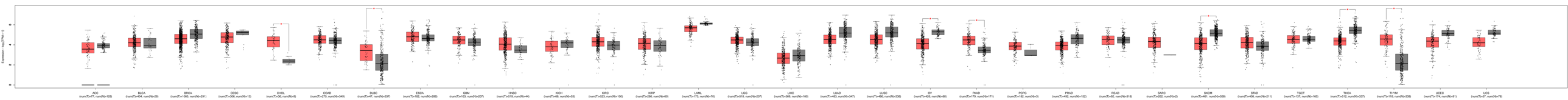

Supplement: Supplementary file 1 [file ncrna-08-00051-s001.zip › Supplementary Figure S3.pdf]

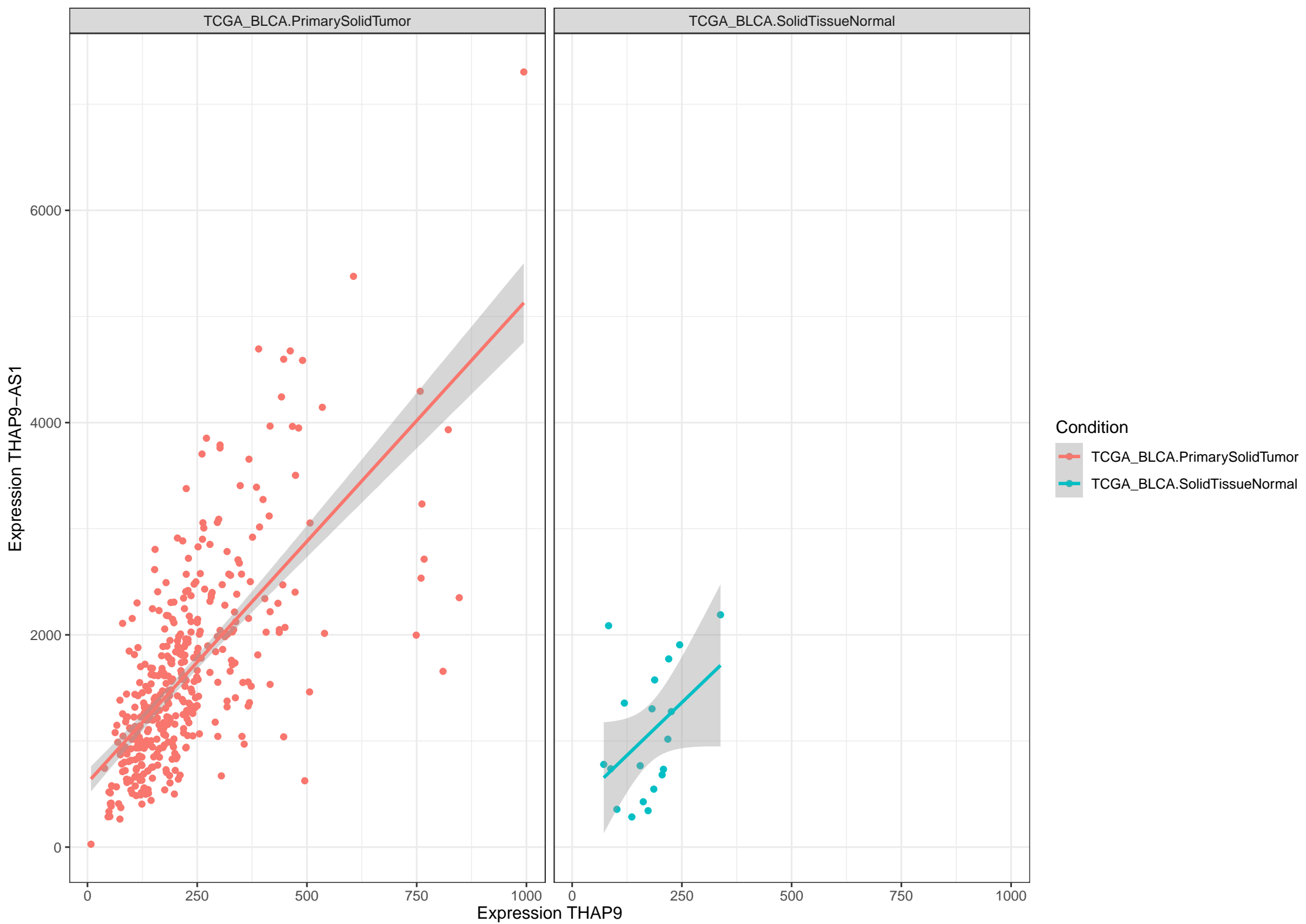

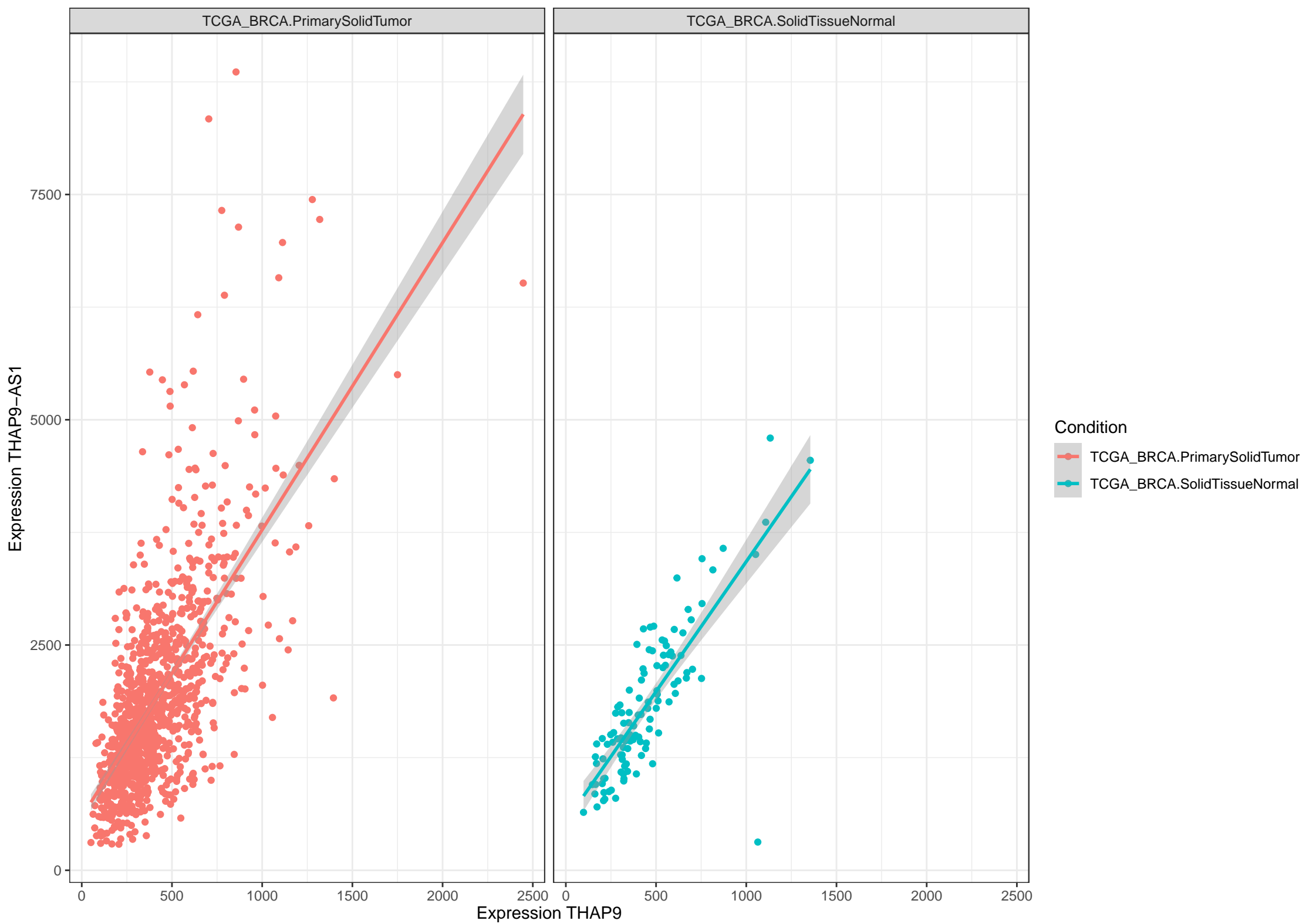

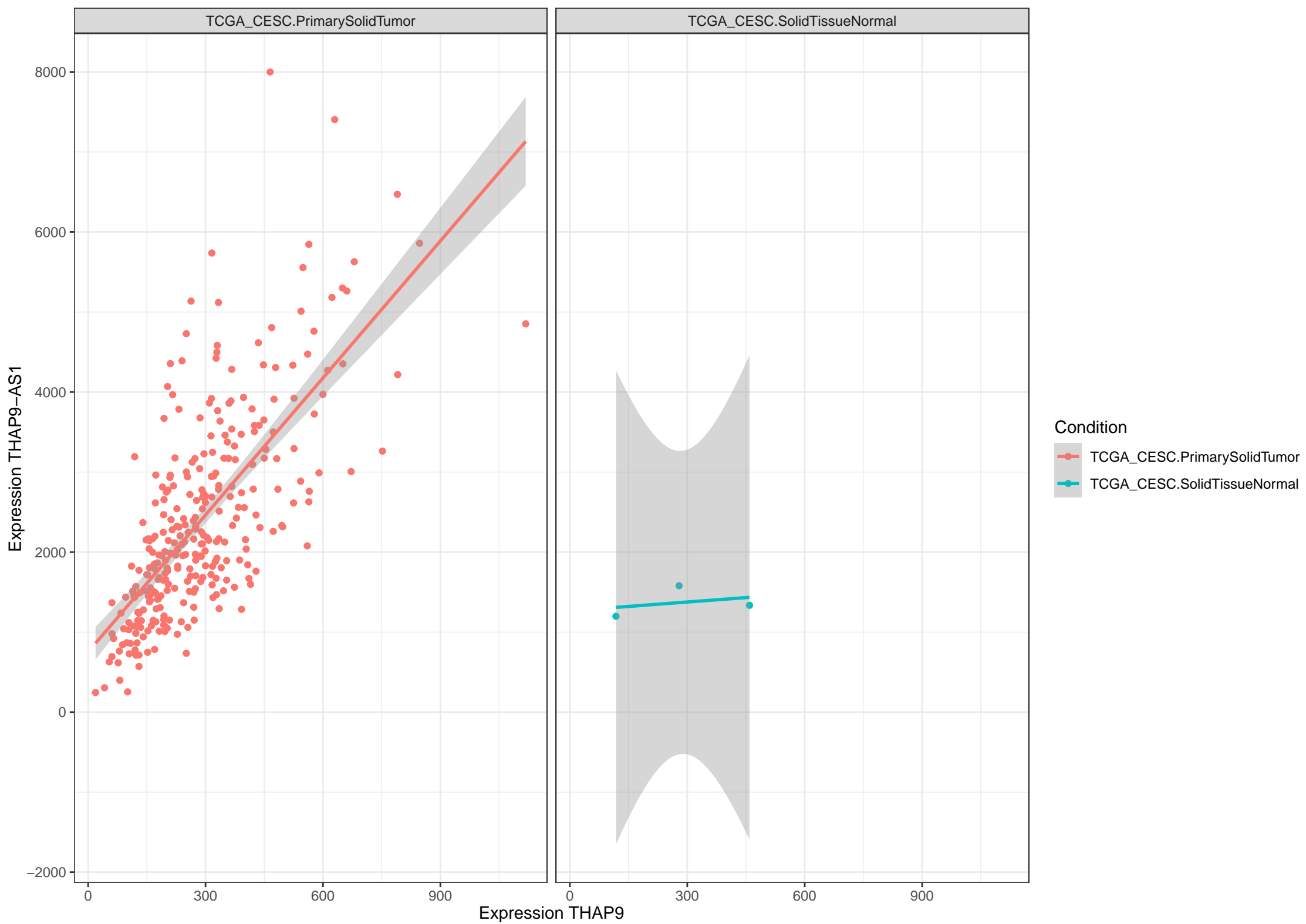

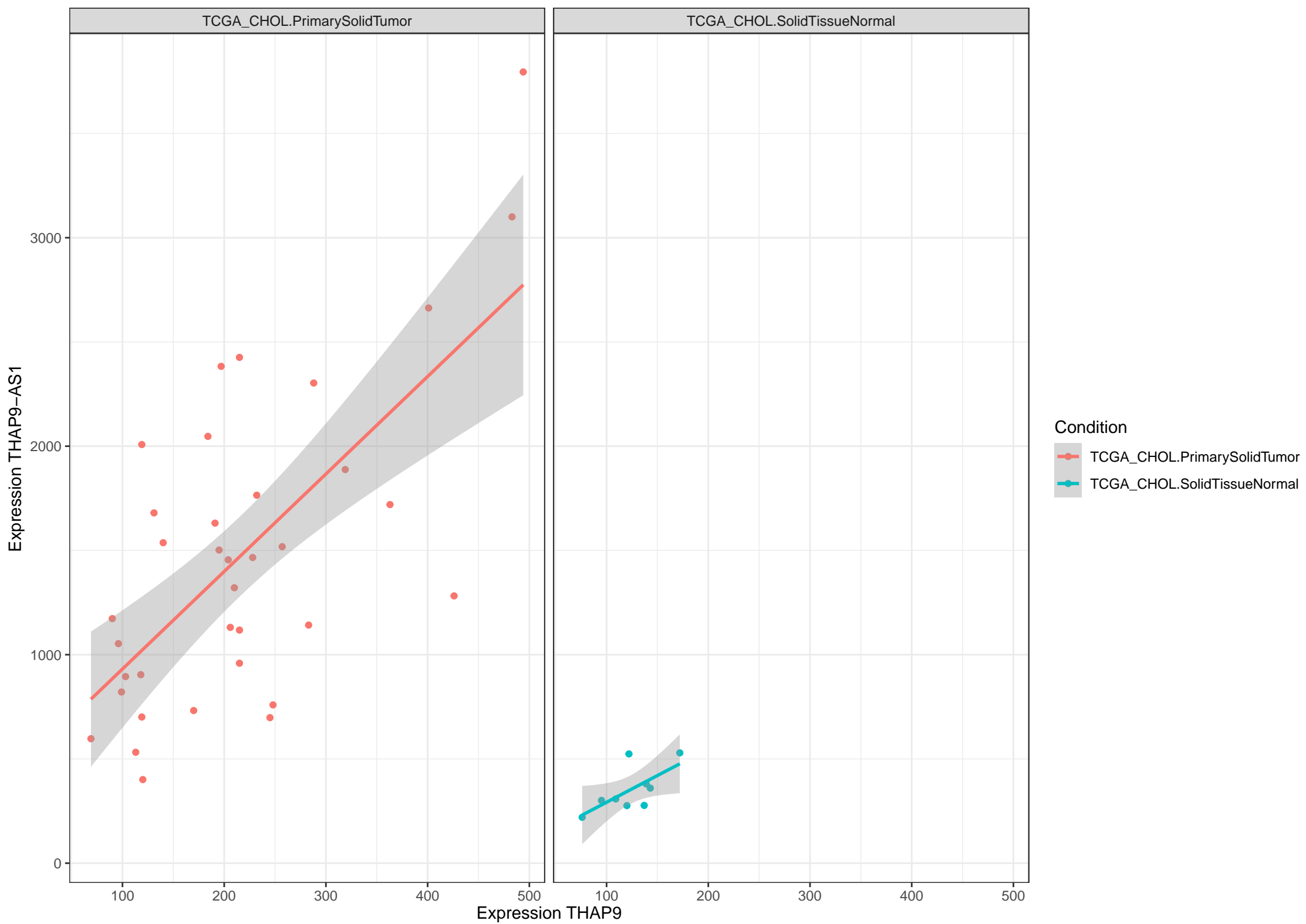

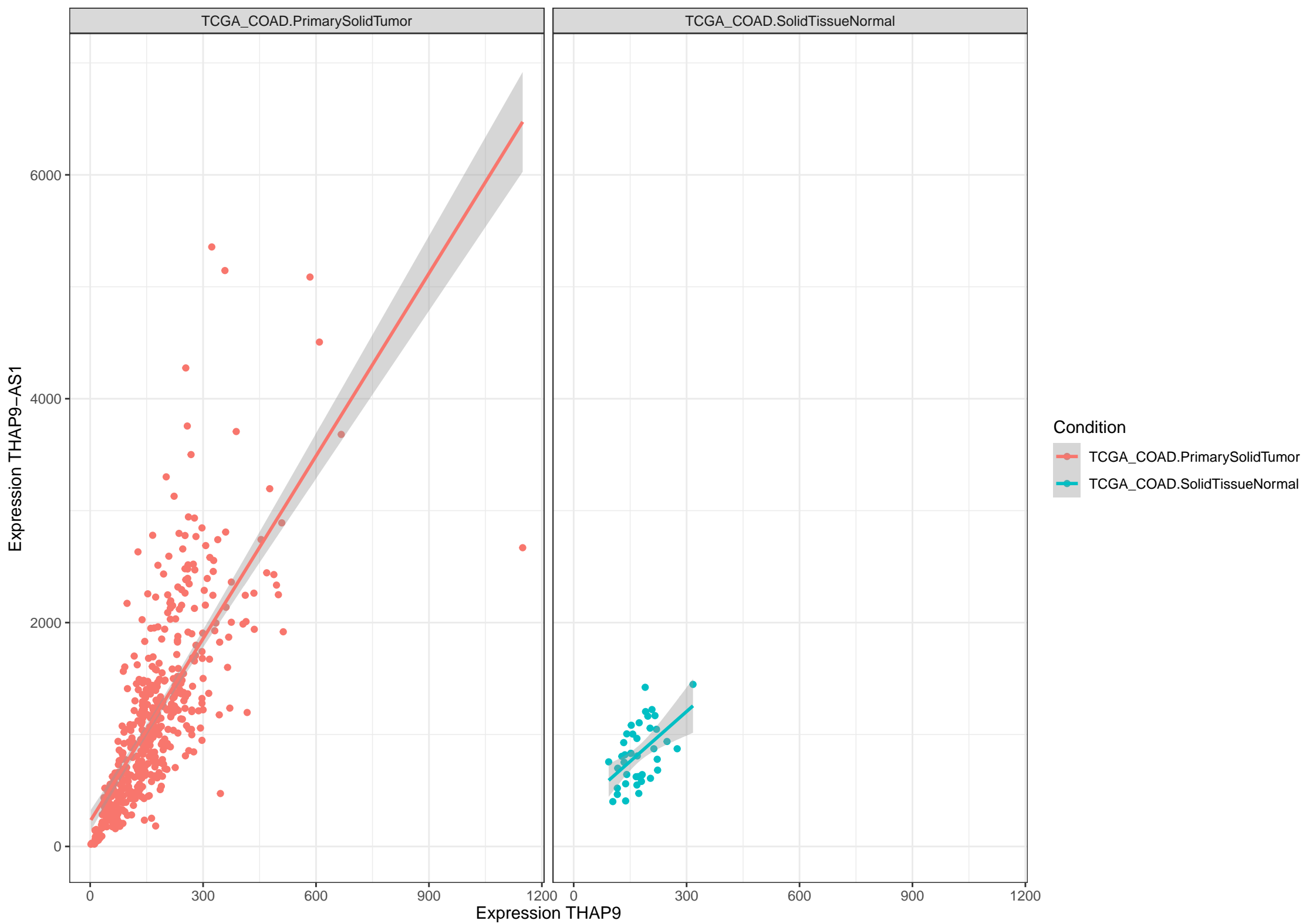

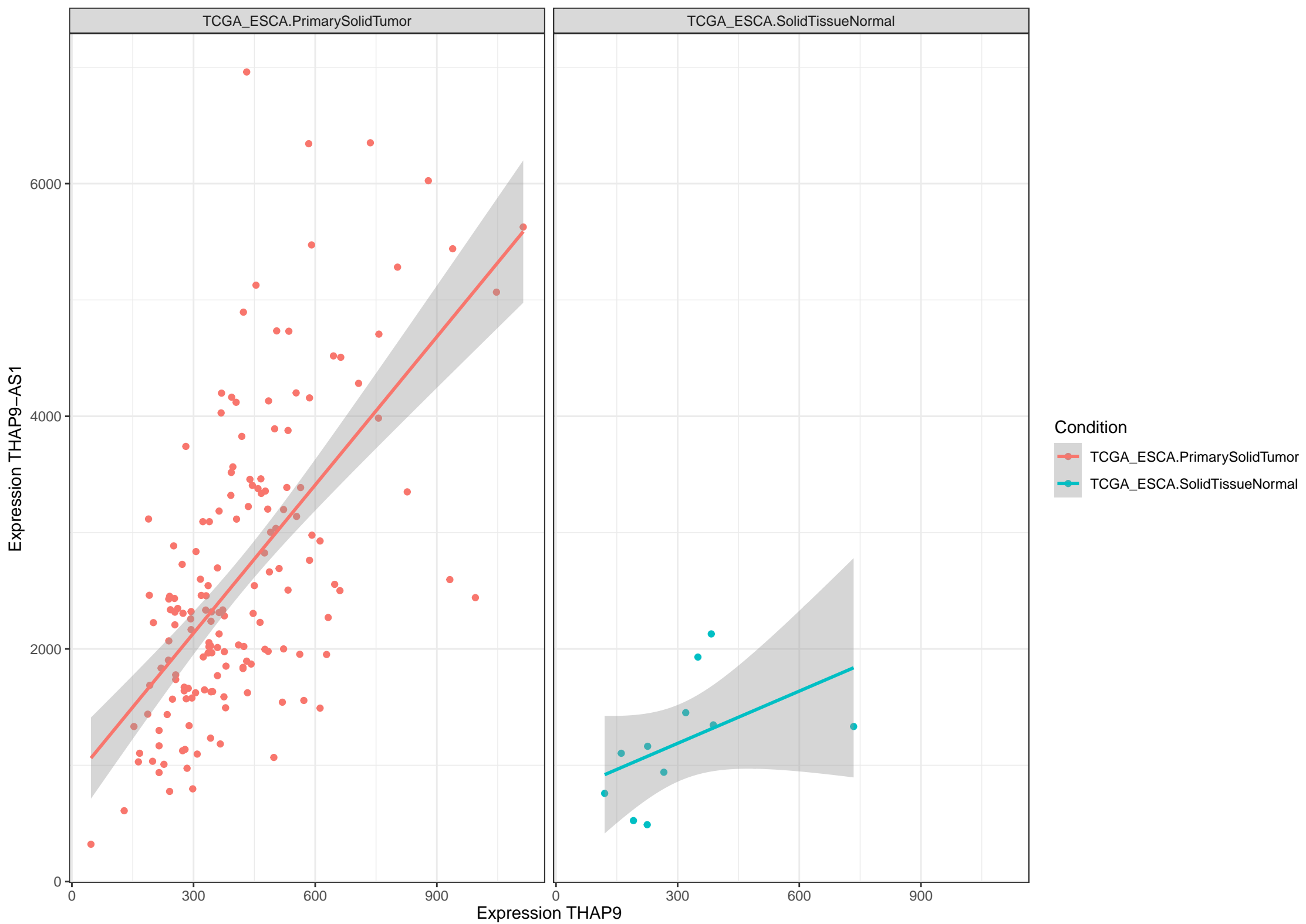

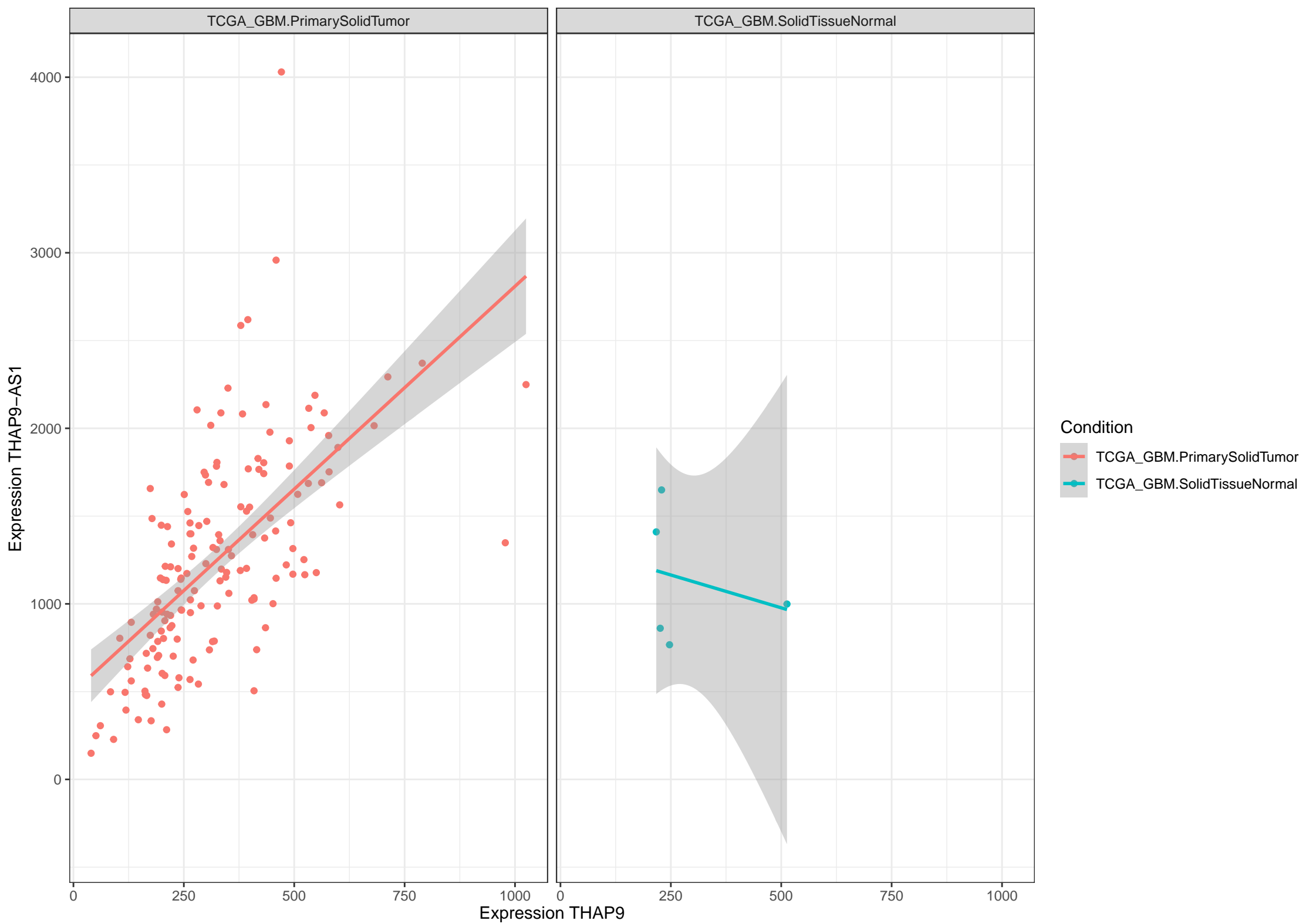

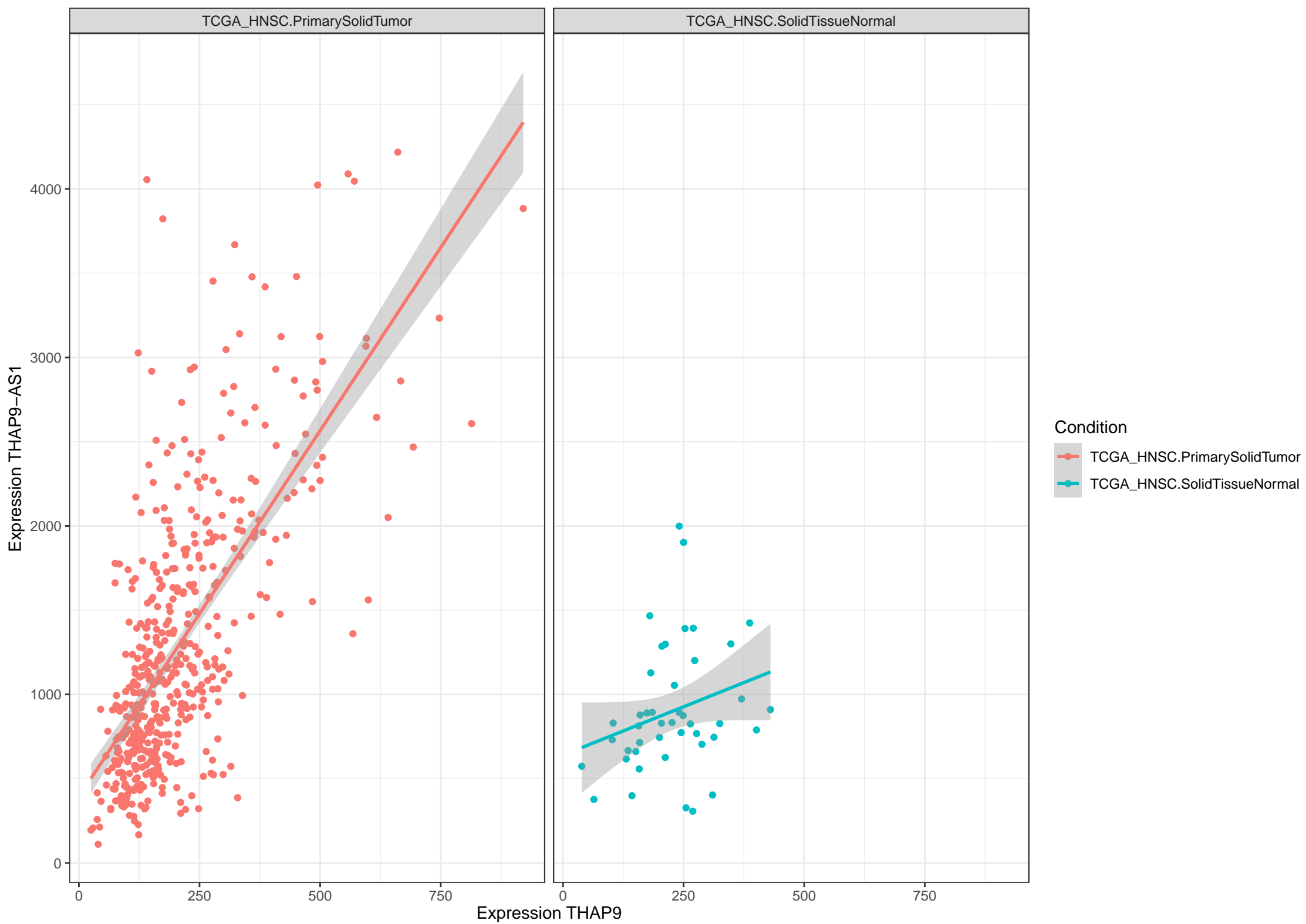

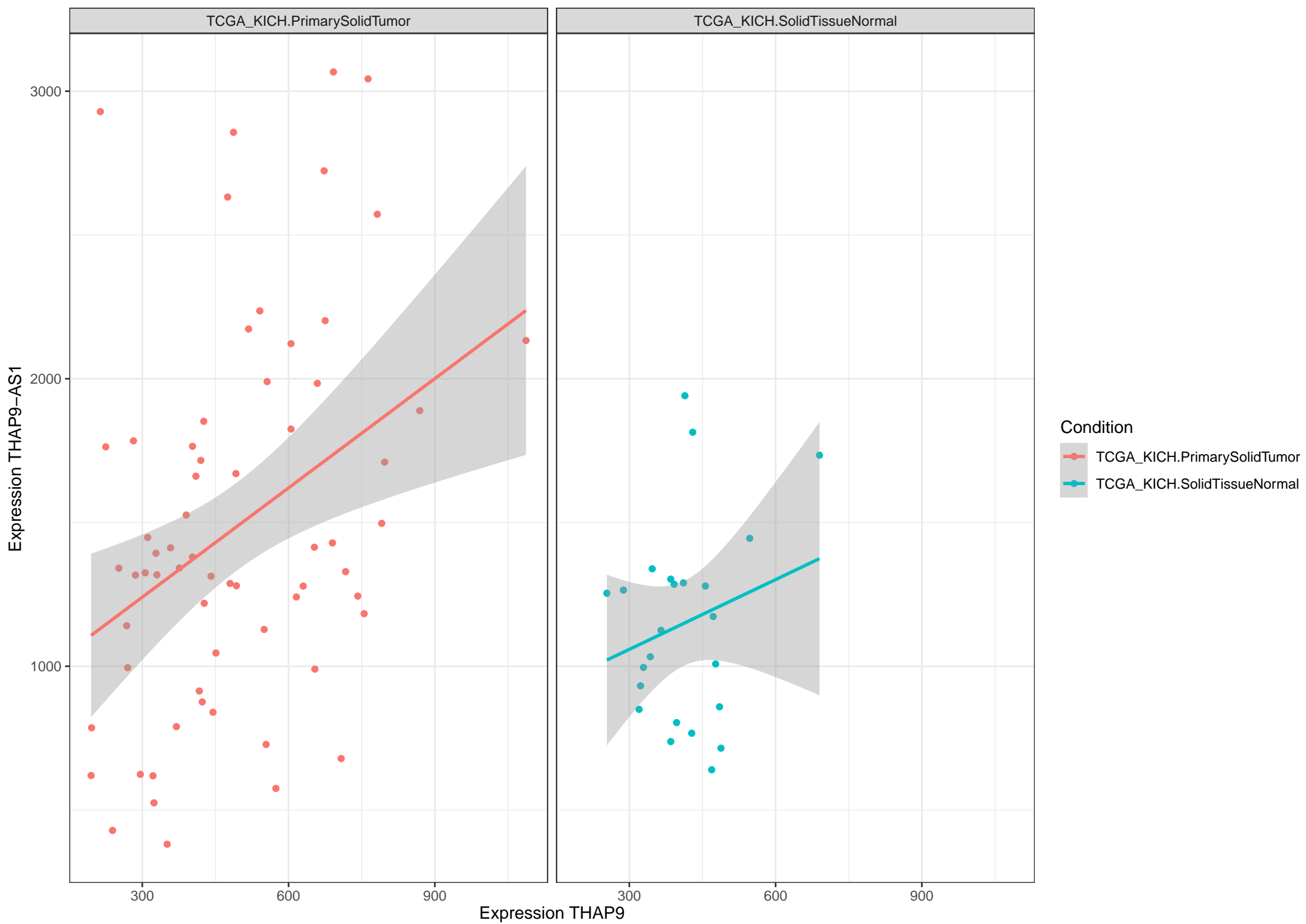

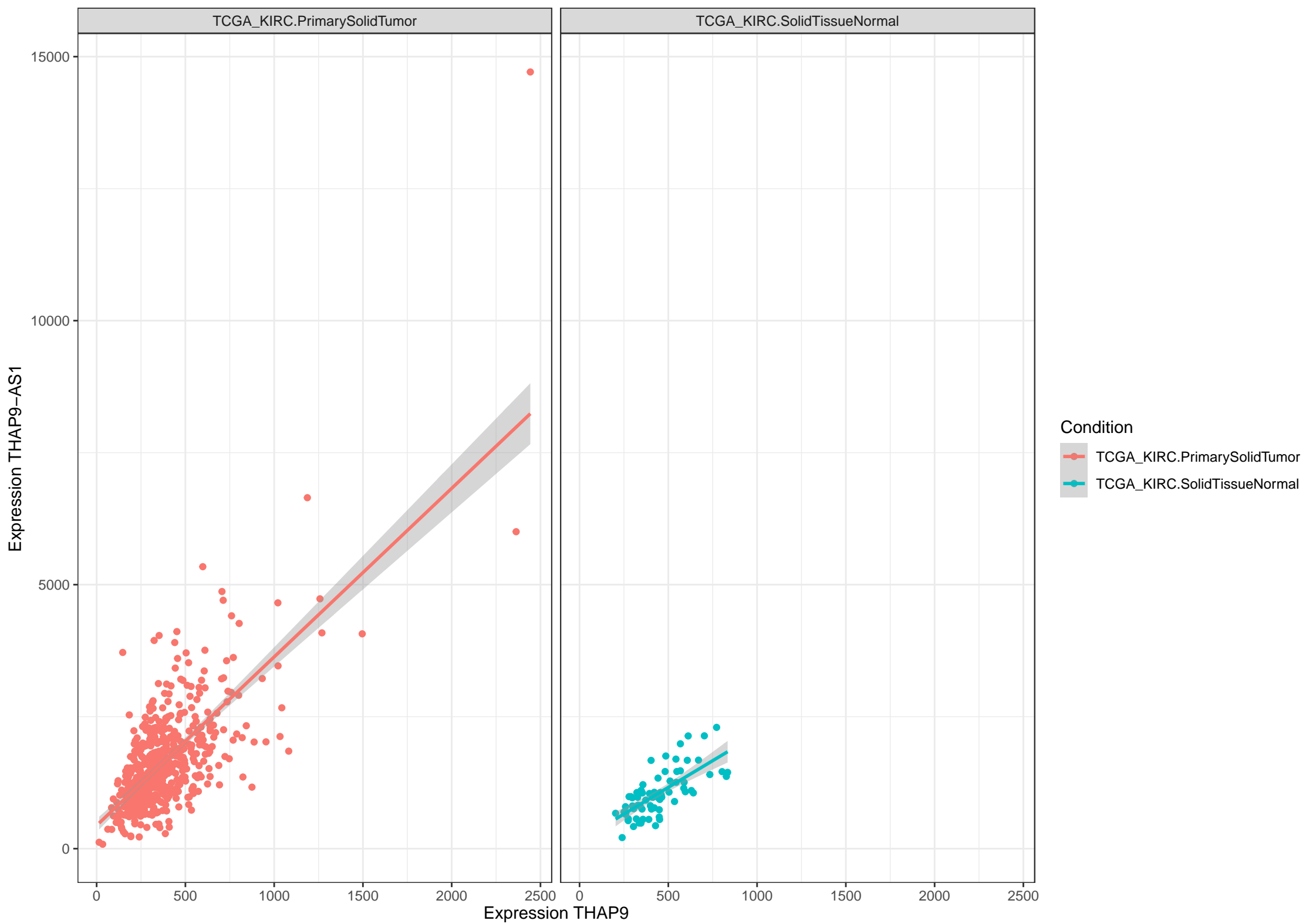

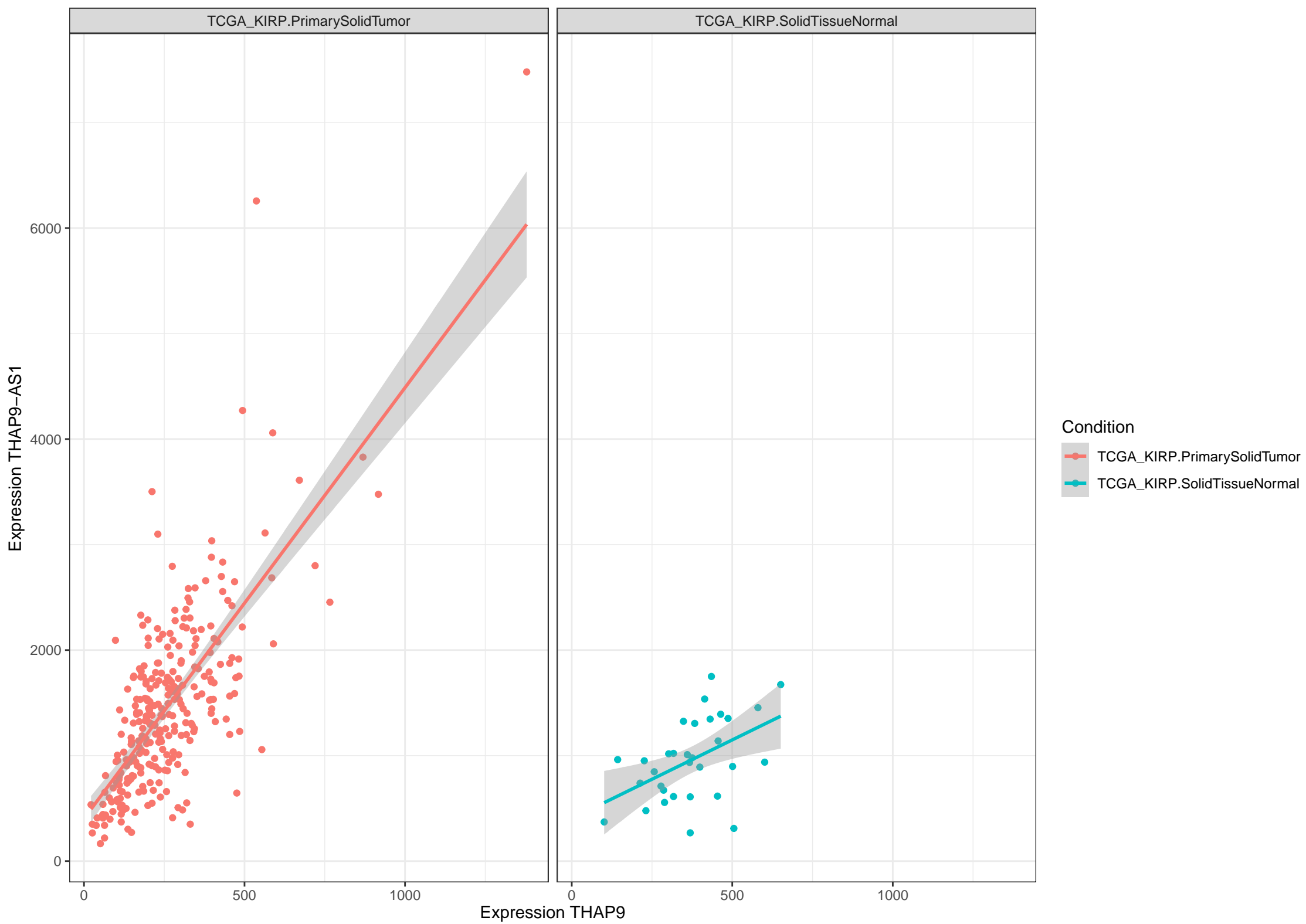

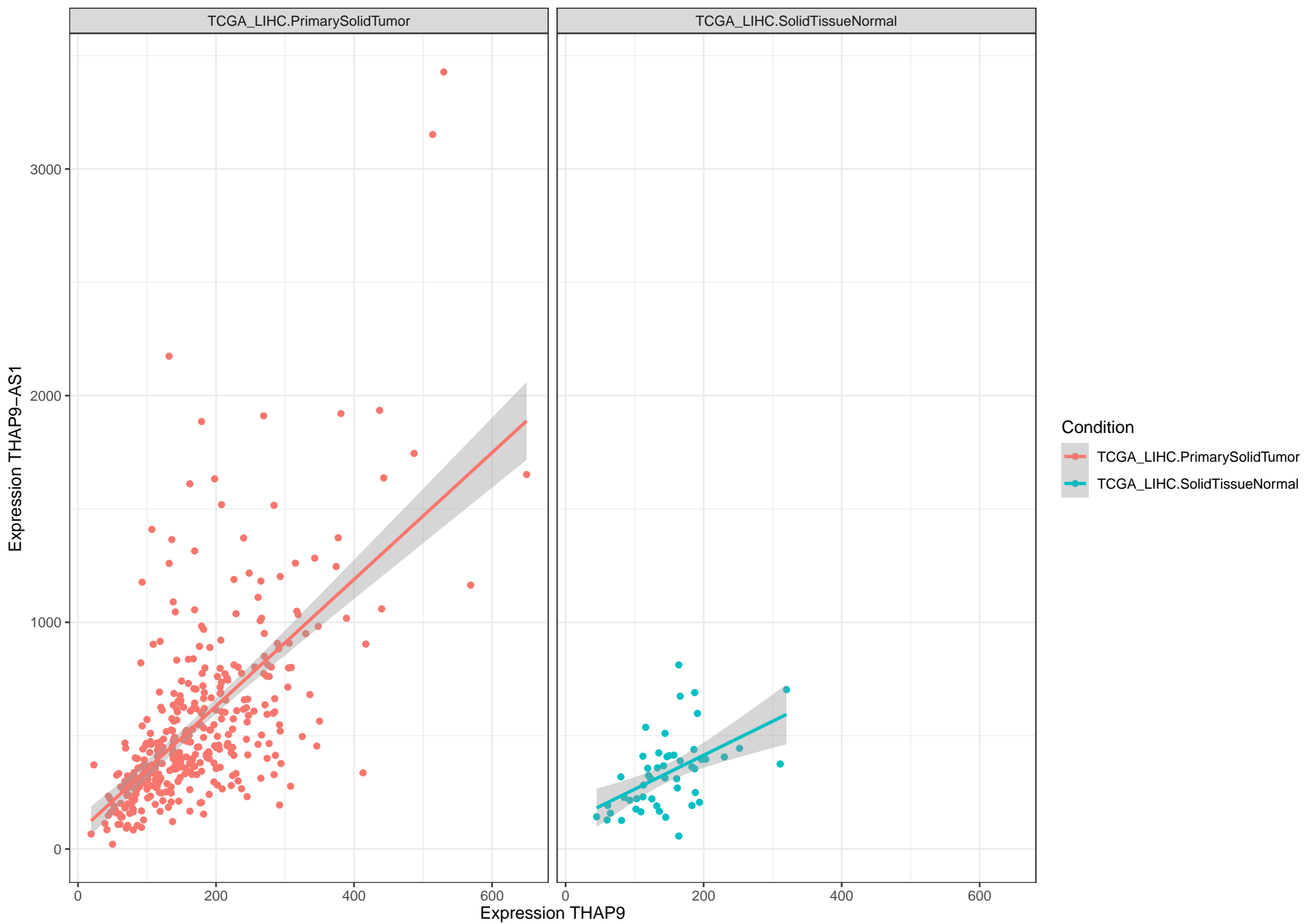

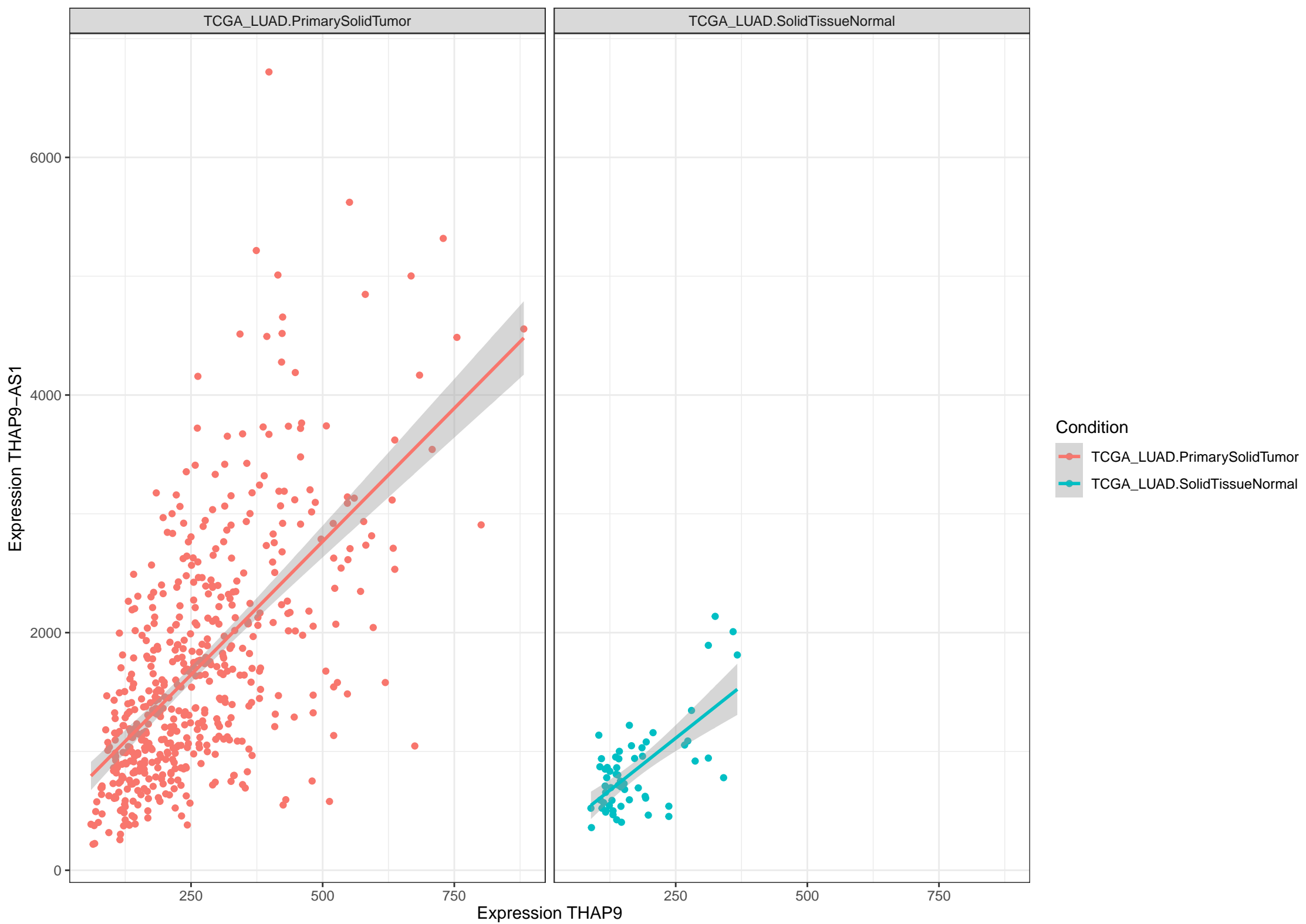

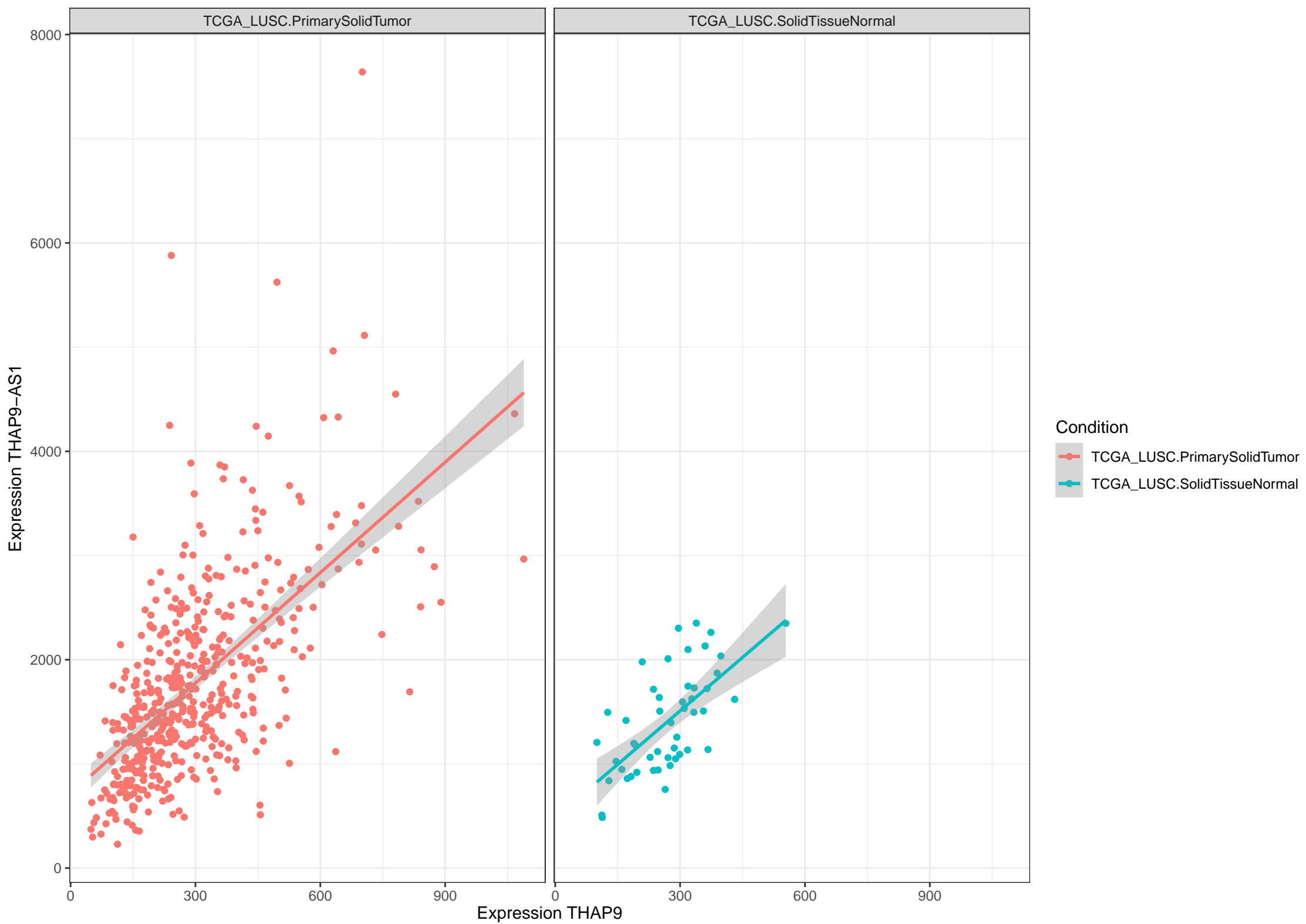

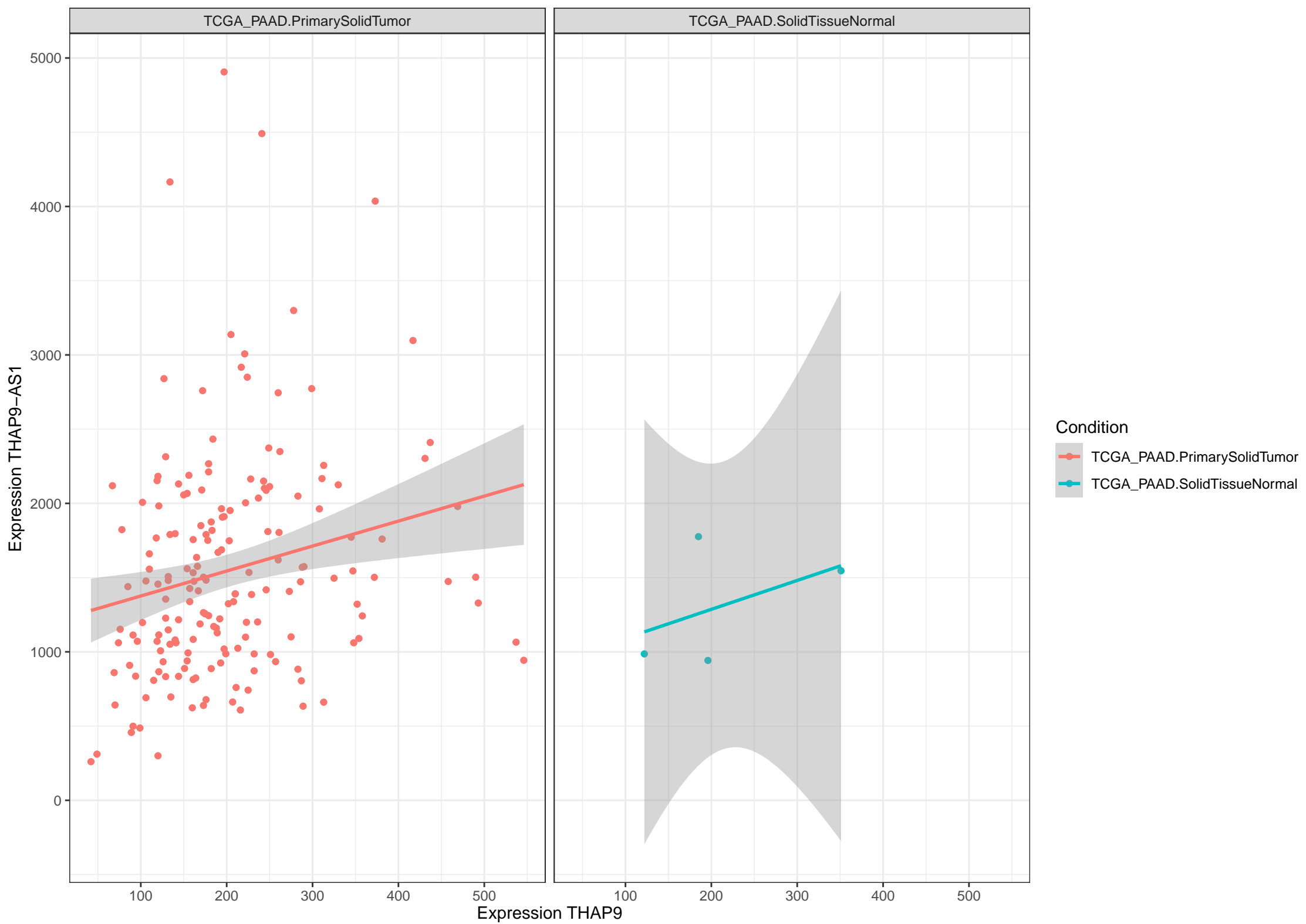

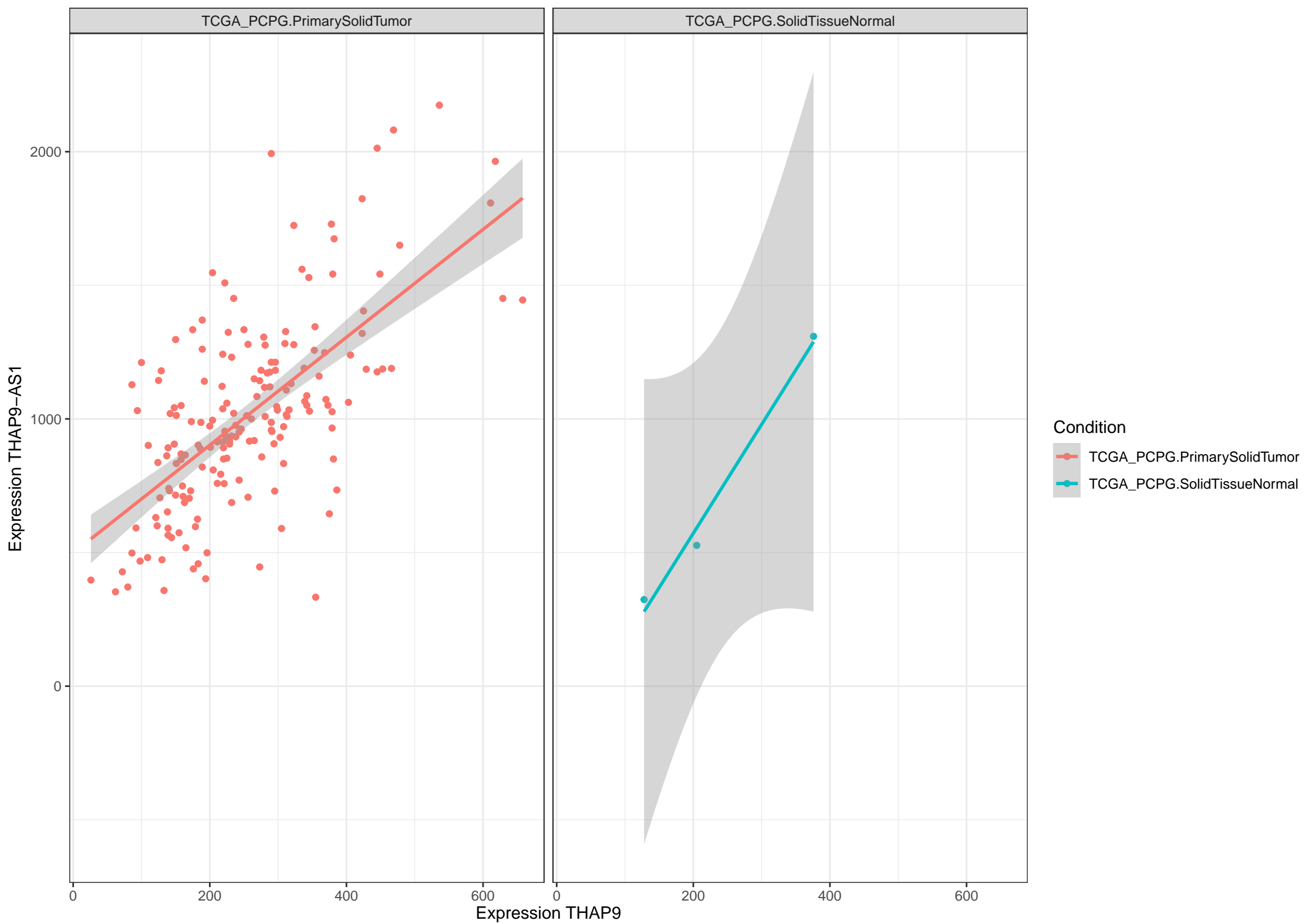

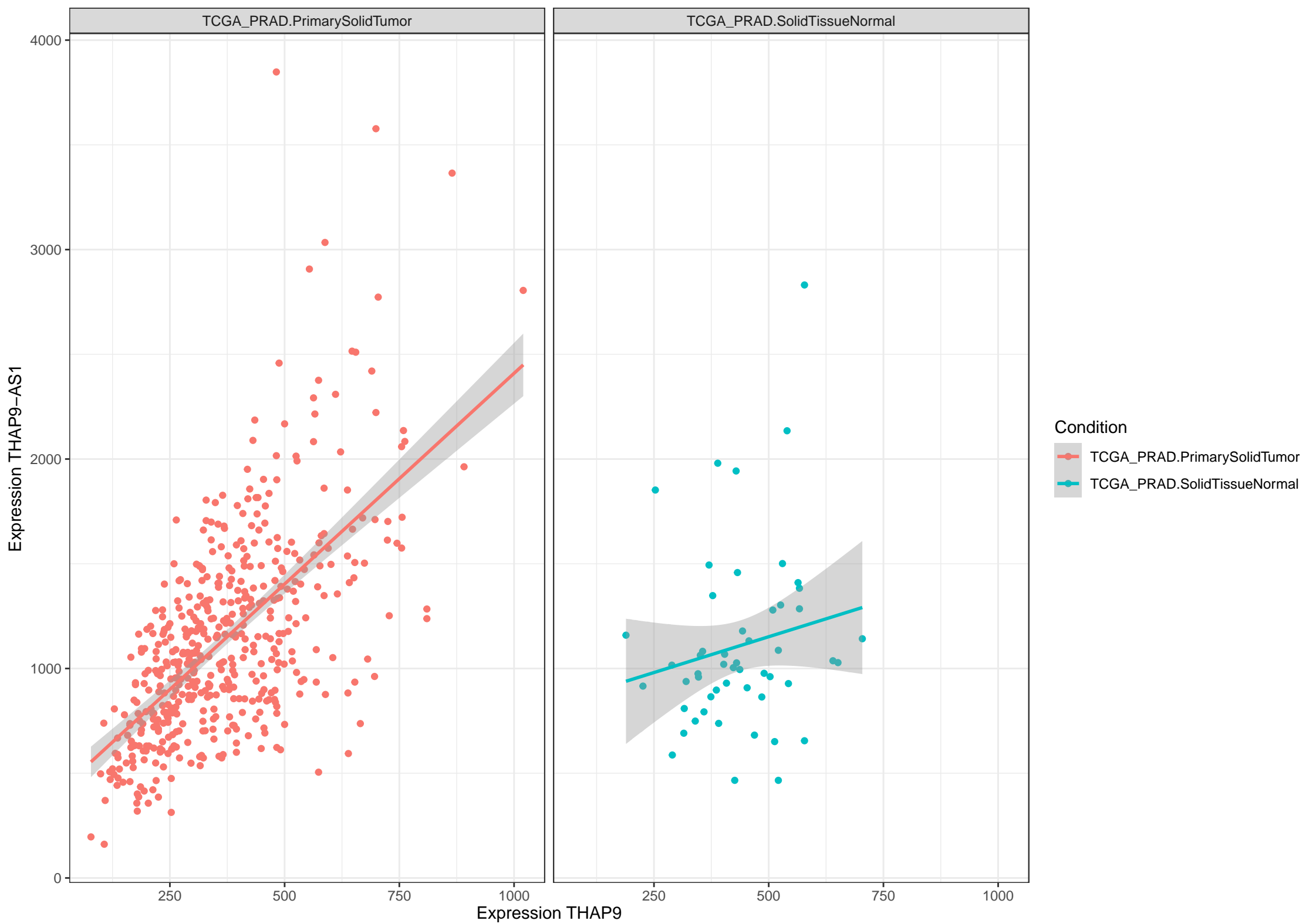

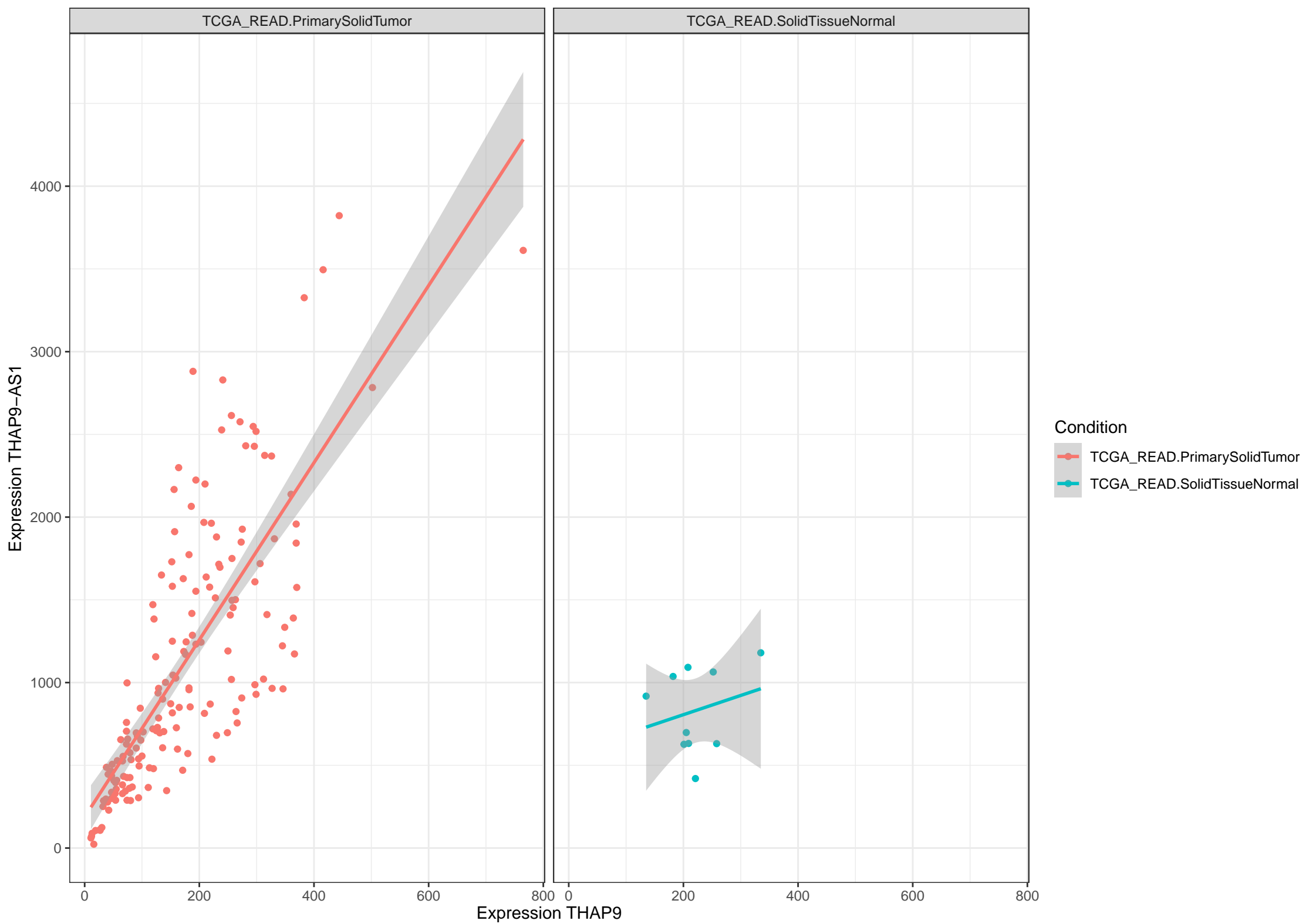

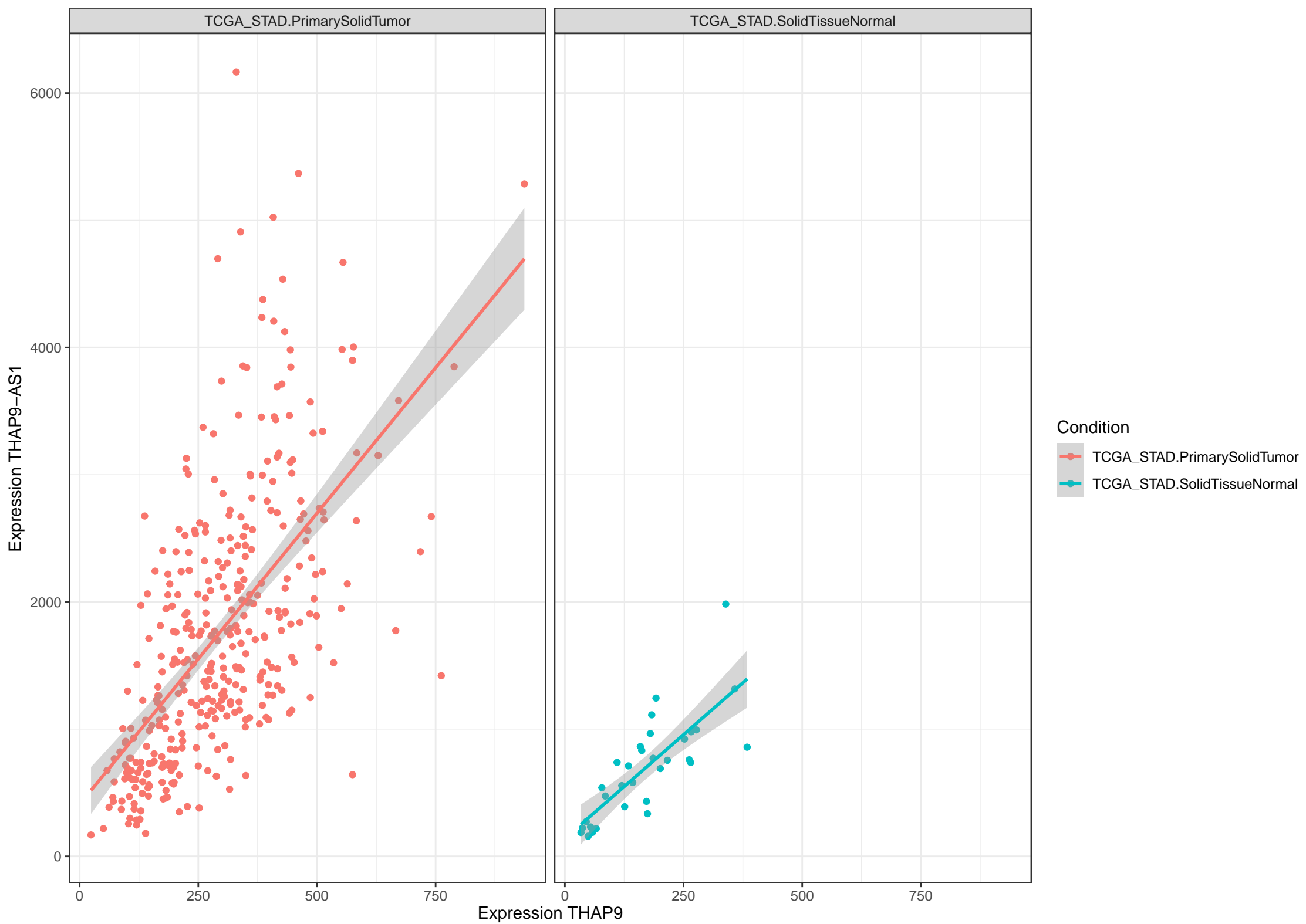

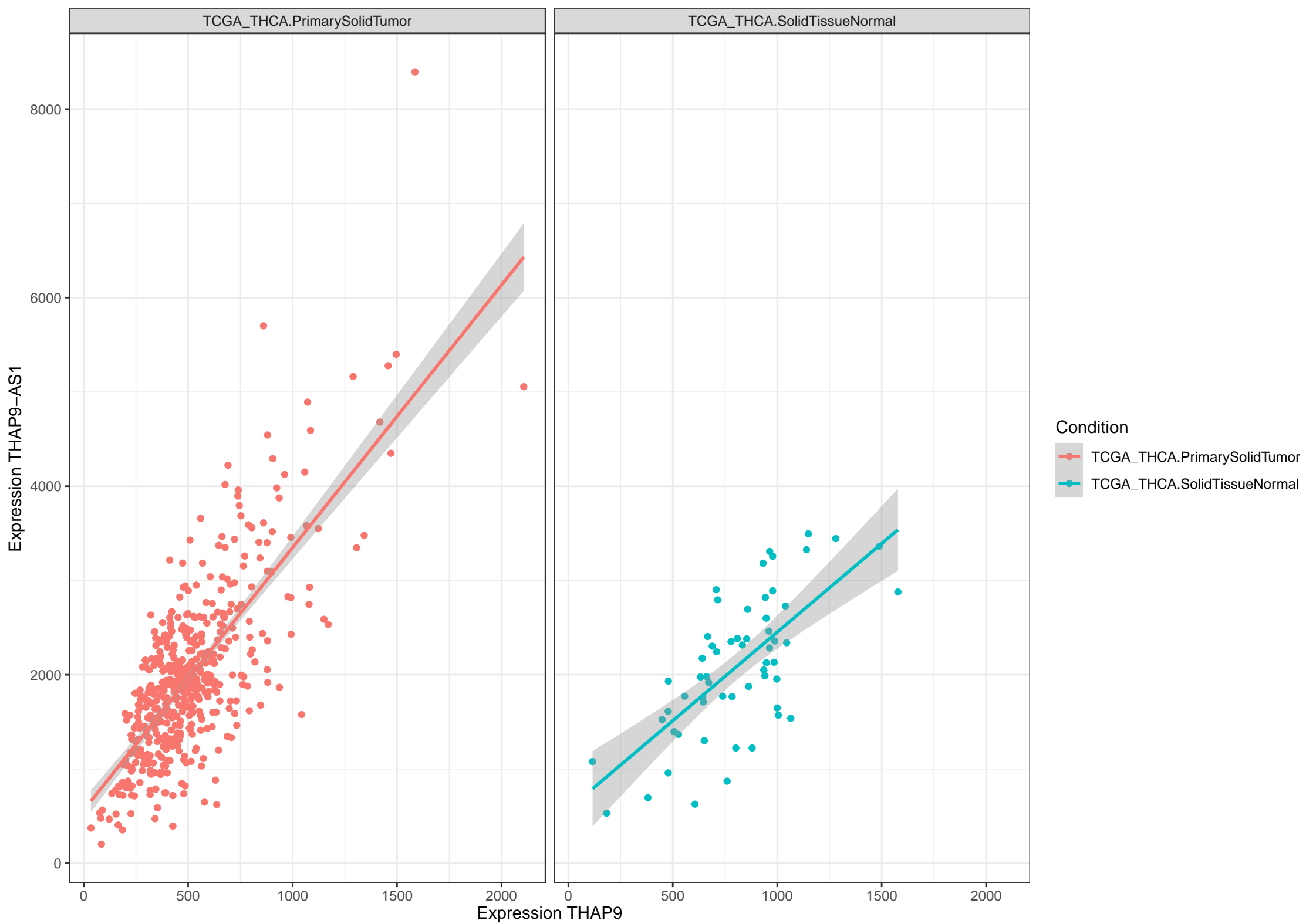

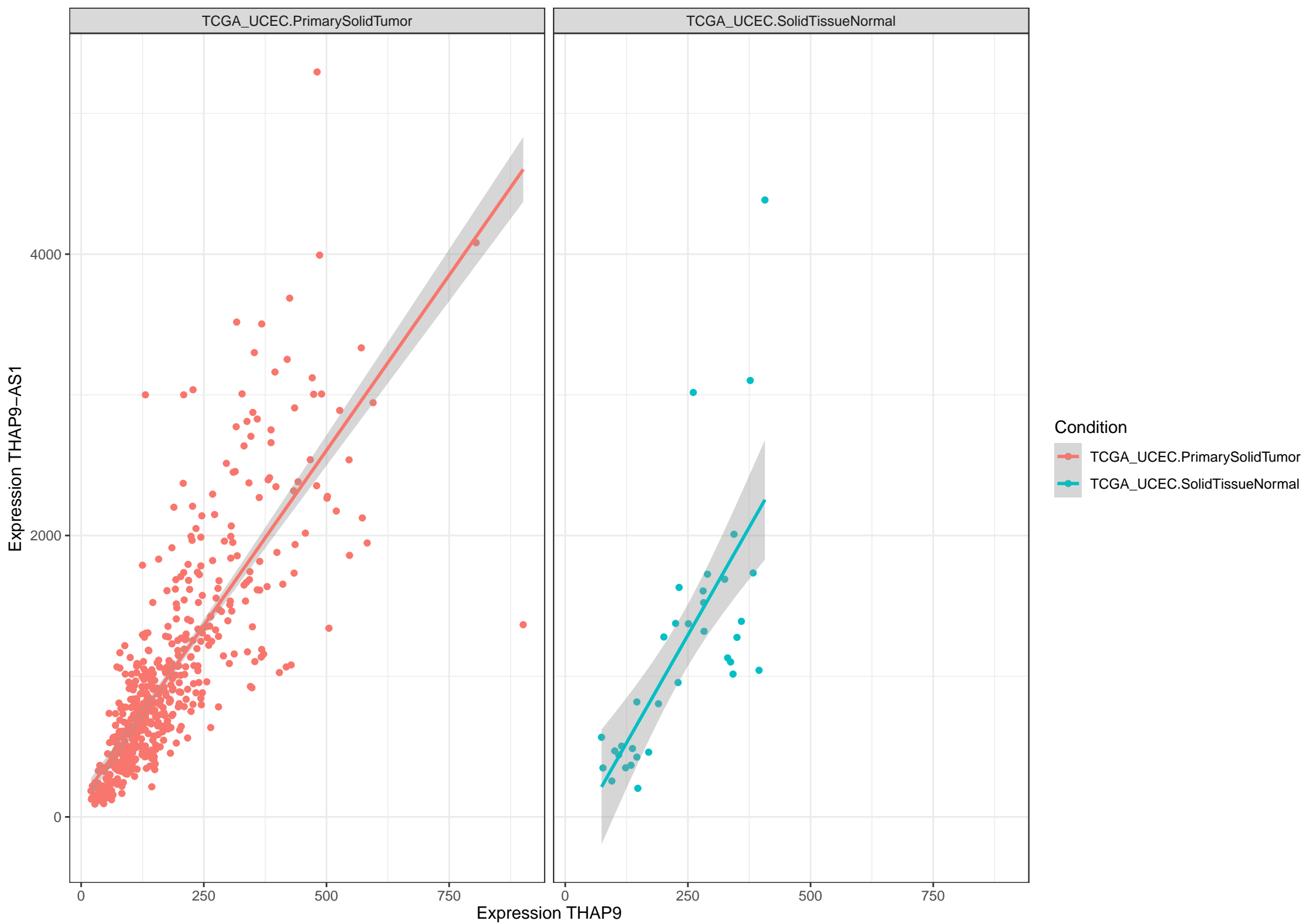

Supplement: Supplementary file 1 [file ncrna-08-00051-s001.zip › Supplementary Figure S4.pdf]
